# Apolipocrustacein, formerly vitellogenin, is the major egg yolk precursor protein in decapod crustaceans and is homologous to insect apolipophorin II/I and vertebrate apolipoprotein B

**DOI:** 10.1186/1471-2148-7-3

**Published:** 2007-01-22

**Authors:** Jean-Christophe Avarre, Esther Lubzens, Patrick J Babin

**Affiliations:** 1 Israel Oceanographic and Limnological Research, P.O. Box 8030, Haifa 31080, Israel; 2 Genewave XTEC, Ecole Polytechnique, 91128 Palaiseau, France; 3 Génomique et Physiologie des Poissons, Université Bordeaux 1, UMR NuAGe, 33405 Talence cedex, France

## Abstract

**Background:**

In animals, the biogenesis of some lipoprotein classes requires members of the ancient large lipid transfer protein (LLTP) superfamily, including the cytosolic large subunit of microsomal triglyceride transfer protein (MTP), vertebrate apolipoprotein B (apoB), vitellogenin (Vtg), and insect apolipophorin II/I precursor (apoLp-II/I). In most oviparous species, Vtg, a large glycolipoprotein, is the main egg yolk precursor protein.

**Results:**

This report clarifies the phylogenetic relationships of LLTP superfamily members and classifies them into three families and their related subfamilies. This means that the generic term Vtg is no longer a functional term, but is rather based on phylogenetic/structural criteria. In addition, we determined that the main egg yolk precursor protein of decapod crustaceans show an overall greater sequence similarity with apoLp-II/I than other LLTP, including Vtgs. This close association is supported by the phylogenetic analysis, i.e. neighbor-joining, maximum likelihood and Bayesian inference methods, of conserved sequence motifs and the presence of three common conserved domains: an N-terminal large lipid transfer module marker for LLTP, a DUF1081 domain of unknown function in their central region exclusively shared with apoLp-II/I and apoB, and a von Willebrand-factor type D domain at their C-terminal end. Additionally, they share a conserved functional subtilisin-like endoprotease cleavage site with apoLp-II/I, in a similar location.

**Conclusion:**

The structural and phylogenetic data presented indicate that the major egg yolk precursor protein of decapod crustaceans is surprisingly closely related to insect apoLp-II/I and vertebrate apoB and should be known as apolipocrustacein (apoCr) rather than Vtg. These LLTP may arise from an ancient duplication event leading to paralogs of Vtg sequences. The presence of LLTP homologs in one genome may facilitate redundancy, e.g. involvement in lipid metabolism and as egg yolk precursor protein, and neofunctionalization and subfunctionalization, e.g. involvement in clotting cascade and immune response, of extracellular LLTP members. These protein-coding nuclear genes may be used to resolve phylogenetic relationships among the major arthropod groups, especially the Pancrustacea-major splits.

## Background

In 1967, Wallace *et al*. [1] characterized a high-density lipoprotein from decapod crustaceans ovaries with similar biochemical properties to lipoproteins isolated from vertebrate eggs, and proposed the generic term "lipovitellin" for this abundant lipoprotein. Two years later, Kerr [2] identified a blood-borne protein present only in female blue crabs *Callinectes sapidus *with developing oocytes. This lipoprotein turned out to be serologically identical to oocyte lipovitellin. The term "vitellogenin" (Vtg) was proposed over thirty-five years ago [3] to describe female-specific insect hemolymph protein precursors of egg yolk, regardless of their amino acid sequences or structures. This term, based on a functional criterion, was later adopted in other egg-laying animals, including crustaceans [4], and is widely used in the scientific community and sequence databases. Molecular characterization of Vtg in numerous oviparous species has revealed that this high molecular weight glycolipoprotein is conserved among species, suggesting derivation from a common ancestor [5-8]. However, molecular data obtained in some species has revealed that the main egg yolk precursor proteins are unrelated to the Vtg protein family. For example, major egg yolk precursor protein is related to transferrin in sea urchins [9,10] and lipase in higher *Diptera *[11]. Multiple alignments of vertebrate and non-vertebrate Vtg sequences revealed five relatively well-conserved regions [6,12]. Regions I to III, located in the N-terminal part, correspond to the lipovitellin 1 subunit of vertebrate Vtg, while regions IV and V, located in the C-terminal part, correspond to the lipovitellin 2 subunit. Sequence and deduced structural homologies indicated an evolutionary relationship of Vtg with three mammalian proteins, apolipoprotein B100 (apoB), the large subunit of microsomal triglyceride transfer protein (MTP), and the von Willebrand factor [13,14]. The identification of conserved amino acid sequence motifs and ancestral exon boundaries in apoB, MTP, non-vertebrate and vertebrate Vtg, and insect apolipophorin II/I (apoLp-II/I) indicated that large lipid transfer proteins (LLTP) are members of the same multigene superfamily and have emerged from a common ancestral molecule designed to play a pivotal role in the intracellular and extracellular transfer of lipids and liposoluble substances [15,16].

Knowledge of molecular structure and expression of Vtg in oviparous animals has increased impressively over the past two decades [6,17]. Recent molecular characterization and expression studies of the main egg yolk precursor protein, referred to as Vtg, in over ten decapod crustacean species suggests that this precursor protein is atypical in regard to Vtg from other oviparous animals [18-29]. In addition, it has been shown that the crustacean clotting protein (CP), a very high density lipoprotein (VHDL) responsible for hemolymph clot formation, is also a Vtg-related protein [30,31]. The aim of this study was therefore to clarify the phylogenetic relationship of these crustacean Vtg-related proteins with other LLTP superfamily members. The results presented here led us to call apolipocrustacein (apoCr) rather than Vtg the major decapod crustacean egg yolk precursor protein.

## Results and discussion

### Crustacean apoCr sequences

Full-length apoCr cDNA sequences, annotated as Vtg in the GenBank™/EBI Data Bank (see the Methods section), are currently available for eleven decapod species: *Penaeus semisulcatus*, *Penaeus monodon*, *Metapenaeus ensis*, *Marsupenaeus japonicus*, *Litopenaeus vannamei*, *Feneropenaeus merguiensis*, *Cherax quadricarinatus*, *Machrobrachium rosenbergii*, *Charybdis feriatus, Pandalus hypsinotus*. and *Portunus trituberculatus*. In *Penaeus semisulcatus *and *Marsupenaeus japonicus*, the same apoCr cDNA was isolated from ovary and hepatopancreas tissues [23,32]. In *Metapenaeus ensis*, two apoCr cDNAs, apoCr1 and apoCr2, were isolated from these two tissues [24,25]. They shared 56% sequence identity and showed a tissue-specific expression pattern. These two apoCr may result from a gene duplication event in the *Metapenaeus *lineage. A recent phylogeny of penaeid shrimps, performed on two mitochondrial genes, indicated that *Metapenaeus *may be representative of the ancient *Penaeus *genus but distant from the other penaeid species [33]. Alignment of the deduced amino acid sequence of *Penaeus semisulcatus *revealed a sequence identity ranging from 92% with *Feneropenaeus merguiensis *to 34% with *Charybdis feriatus *[see Additional file 1]. The phylogenetic analysis resulting from this alignment (data not shown) indicated that these deduced precursor proteins may be confidently grouped according to the species tree; i.e. the penaeid shrimps of *Dendrobranchiata *suborder were grouped together and the *Pleocyemata *suborder species, crab (*Charybdis feriatus*), crayfish (*Cherax quadricarinatus*) and prawn (*Machrobrachium rosenbergii*, and *Pandalus hypsinotus*) were outside this monophyletic group. It is interesting to note that phylogenetic results correlated with the expression pattern of apoCr transcripts. *Dendrobranchiata *suborder species express apoCr transcripts in both ovaries and hepatopancreas [18,19,23,24,28,29], while the expression of paralogous apoCr2 from *Metapenaeus ensis *[24,25] and apoCr of *Pleocyemata *suborder species is apparently restricted to the hepatopancreas [20,21,26,27].

### Global sequence alignment of crustacean apoCr with other LLTP

A BLASTP [34] search of the nonredundant GenBank™ database using *Penaeus semisulcatus *apoCr as a target sequence revealed high BLAST scores and expected (E) values with other decapod apoCr, from the full-length sequence of *Feneropenaeus merguiensis *(score 4530, E value 0.0) to *Charybdis feriatus *(score 1134, E value 0.0). There were also similarities with other extracellular LLTP: insect apoLp-II/I, from *Locusta migratoria *(score 296, E value 7e-78) to *Anopheles gambiae *(score 173, E value 9e-41), and vertebrate apoB, from *Gallus gallus *(score 115, E value 3e-23) to *Danio rerio *(score 105, E value 4e-20), including *Homo sapiens *apoB-100 precursor protein (score 105, E value 2e-20). Lower scores and E values were retrieved with Vtg of oviparous animals, ranging from *Crassostrea gigas *mollusks (score 100, E value 1e-18) to *Samia cynthia ricini *insects (score 40, E value 1.2). BLAST scores and E values of intracellular MTP family members overlapped with Vtg family members, as shown with *Strongylocentrotus purpuratus *echinoderms (score 48.9, E value 0.003) or *Homo sapiens *(score 38.9, E value 3.4). Even if BLAST analysis represents an over simplification of reality, the results obtained by this method, i.e. so-called crustacean Vtg are more closely-related to insect apoLp-II/I than to insect Vtg, as indicated by BLAST scores, was not seriously taken into account, as the term Vtg was already used for these sequences.

Crustacean apoCr (~2,600 amino acids) is of intermediate length between insect, nematode, mollusk, and vertebrate Vtg, which are generally shorter (~1,600–1,800 amino acids), and insect apoLp-II/I, which are larger (~3,300 amino acids). Consistent with BLAST scores, crustacean apoCrs confidently align with insect apoLp-II/Is [see Additional file 1]. ApoLp-II/I is the high molecular weight apolipophorin of lipophorin found at high concentrations in insect hemolymph [35-37]. The best local alignments between these sequences occurred along the first 1,000 amino acid residues and in their C-terminal parts [see Additional file 1].

### Domain architecture, conserved sequence motifs, and consensus cleavage site of crustacean apoCr

**Figure 1:**
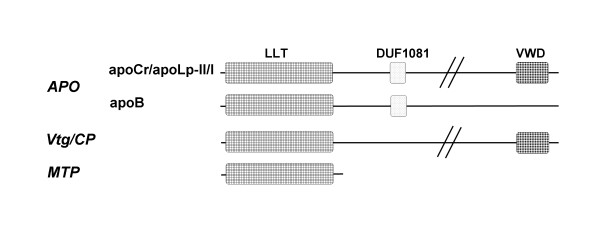
**Domain architecture of large lipid transfer protein (LLTP) superfamily members**. Large lipid transfer (LLT) module [15] also referred to as Vitellogenin_N and LPD-N domain, DUF1081, and VWD domains are indicated from the N-terminal to the C-terminal ends of the proteins. LLTP families are 1) APO family including vertebrate apolipoprotein B (apoB), decapod crustacean apolipocrustacein (apoCr), and insect apolipophorin II/I (apoLp-II/I) subfamilies, 2) Vtg/CP family including vitellogenin (Vtg) and crustacean clotting protein (CP) subfamilies, and the large subunit of microsomal triglyceride transfer protein (MTP) family.

A CD-Search [38] revealed that the amino acid sequence of *Penaeus semisulcatus *and other crustacean apoCr include three conserved structural domains (Figure 1). A lipoprotein N-terminal domain (LPD-N, SMART accession number SM00638), also known as vitellogenin_N (Pfam accession number PF01347) and enclosed in the large lipid transfer (LLT) module [15] was identified in the N-terminal part of apoCr. A DUF1081 domain (Pfam accession number PF06448) of unknown function was identified in the central region, and a von Willebrand-factor type-D domain (VWD) (Pfam accession number PF00094) at the C-terminal end. While the VWD domain, initially identified in the human von Willebrand factor (vWF), is distributed over a wide range of proteins, the LLT module/LPD-N domain, part of the lipovitellin 1 subunit of vertebrate Vtg [6-12], contains twenty-two N-terminal conserved amino acid sequence motifs (N1 to N22) as a common denominator of LLTP [15]. The alignment of these conserved sequence motifs, including those retrieved from apoCr is published as supporting information [see Additional file 2]. Similarly to apoLp-II/I, crustacean apoCr lacks polyserine tracks that correspond to the phosvitin domain in the Vtg of vertebrates and some non-vertebrate species.

In insects, the apoLp-II/I is cleaved before its secretion into apoLp-II and apoLp-I (the subunits were indicated from the N-terminal to the C-terminal ends of the precursor protein) at a consensus cleavage site, RX(R/K)R, for dibasic endoprotease processing [35,37], and this cleavage is mediated by furin [39]. Likewise, crustacean apoCr is also cleaved at a similar dibasic site for subtilisin-like endoprotease processing [18,21,23,24]. This consensus site occurs at amino acid position 725/728 in *Penaeus semisulcatus *(RTRR), between conserved motifs N19 and N20, and aligns very well between crustacean apoCrs and insect apoLp-II/Is [see Additional file 1]. The N-terminal apoCr ~74-kDa subunit in *Penaeus semisulcatus *[23] and other decapod species, apoCr-II, displays high amino acid sequence similarities with the ~80-kDa apoLp-II [35,37]. For example, the *Penaeus semisulcatus *74-kDa apoCr-II subunit displays 22%–43% identity-similarity with *Locusta migratoria *apoLp-II, while *Locusta migratoria *apoLp-II displays no more than 33%–55% and 28%–47% identity-similarity with *Manduca sexta *and *Drosophila melanogaster *apoLp-II, respectively. A similar consensus cleavage site is present in most insect Vtgs, but at a different location, in the extended region between motifs N6 and N7 [15]. However, a potential subtilisin-like convertase site is present between motifs N19 and N20 of nematode and *Crassostrea gigas *Vtgs.

### Phylogenetic analysis and correlation with LLTP domain architecture and protein function

**Figure 2:**
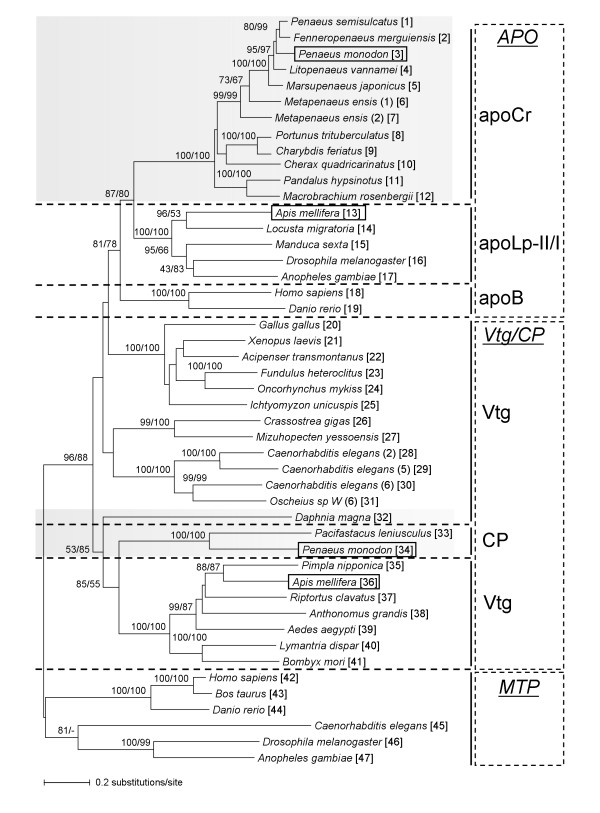
**Neighbor-joining (NJ) and maximum likelihood (ML) phylogenetic reconstruction of the LLTP superfamily**. Tree topology was based on the alignment of the conserved amino acid sequence motifs of the LLT module [see Additional file 2]. Horizontal lengths of branches are proportional to the estimated numbers of amino acid substitutions produced by the NJ algorithm. Numerals at each node show local bootstrap confidence levels estimated by bootstrap pseudoreplicates in NJ and ML, respectively. Numbers are indicated when at least one bootstrap confidence level value at each node is ≥75. The ML inference yielded a similar topology as the NJ tree at the level of significance used. However (-) indicates that this cluster was not formed in the ML analysis. Crustacean sequences are shaded in gray. *Penaeus monodon *apoCr and CP and *Apis melliphera *apoLp-II/I and Vtg are framed as an indication of the presence in one species of homologous extracellular proteins from two different LLTP families. A number in parentheses, corresponding to the extension number used in sequence databases, is attached to the species name, when more than one characterized protein was found in a defined LLTP family. The number in bracket after the species name refers to the code number used for species designation in Figure 3. Abbreviations of LLTP families and subfamilies are as in the legend to Figure 1.

**Figure 3:**
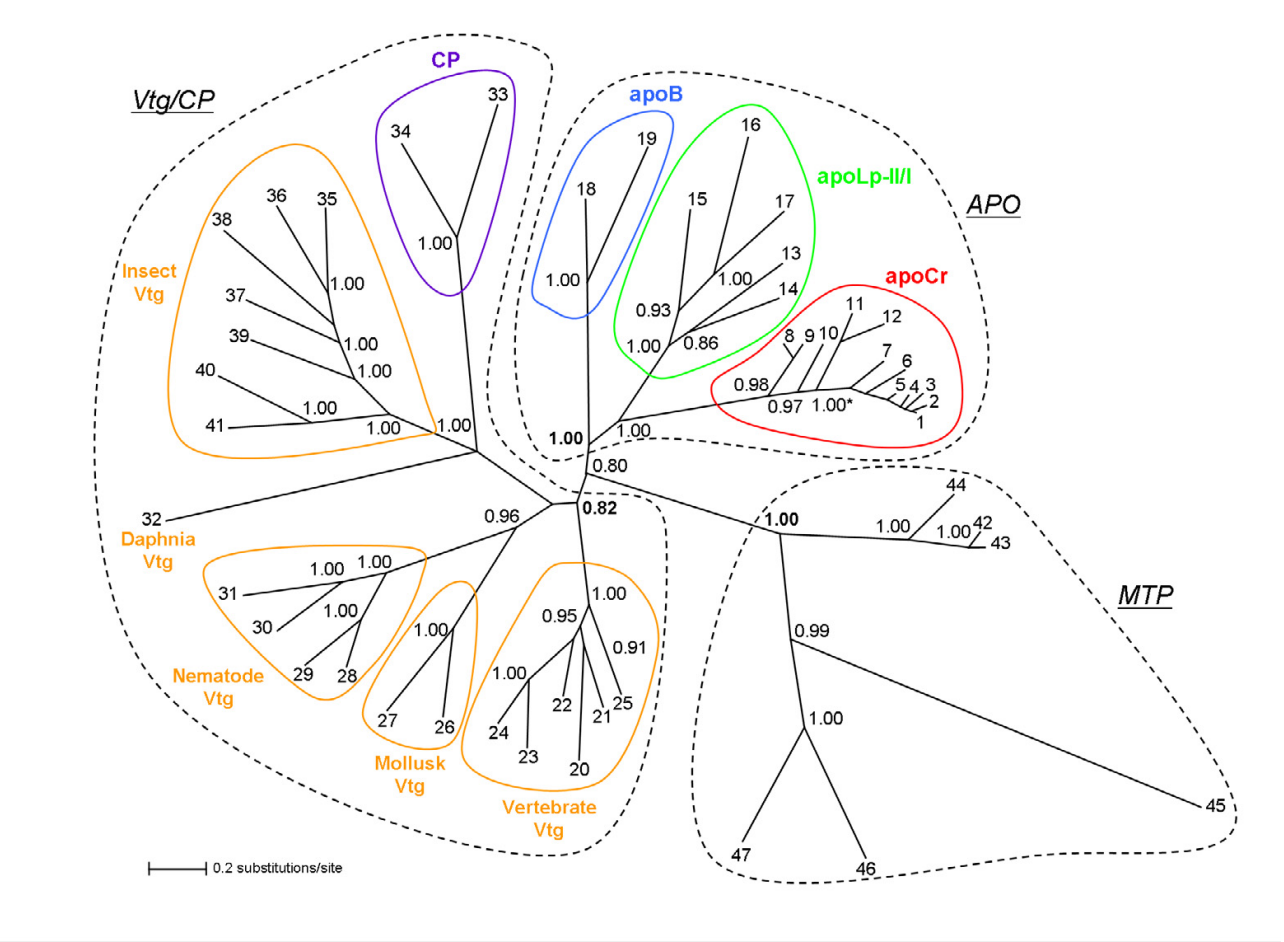
**Bayesian inference (BI) phylogenetic reconstruction of the LLTP superfamily**. The tree was based on the alignment of the conserved amino acid sequence motifs of the LLT module [see Additional file 2]. Species are indicated as numbers and are noted as in Figure 2. Branch lengths are proportionate to BI estimates of numbers of amino acid substitutions and numerals at each node indicate the posterior probability estimated by MrBayes under the model, summed over 9,000 tree samples. Posterior probabilities ≥0.80 are shown. Due to graphical space limitation, 1.00* indicates that the posterior probabilities values of apoCr terminal nodes were 1.00. Abbreviations of LLTP families and subfamilies are as in the legend to Figure 1. The support posterior probability values for LLTP families are bolded.

The evolutionary relationship of genes in the LLTP superfamily was evaluated after aligning the twenty-two conserved N-terminal sequence motifs of the LLT module of selected LLTP sequences [see Additional file 2] and the phylogenetic analyses were conducted by using the neighbor-joining (NJ) and the maximum likelihood (ML) (Figure 2) or the Bayesian inference (BI) (Figure 3) methods. The phylogenetic tree separated extracellular (apoCr, apoLp-II/I, apoB, Vtg, and CP) from intracellular (MTP family) LLTP sequences with confidence into separate clusters, suggesting that these extracellular proteins arose from a common ancestor (hereafter, support bootstrap confidence level values of NJ: 96 and ML: 88, and posterior probability of BI: 0.80). Significant internal branches defined two sequence groups. The first group, named the APO family, suggested that apoCr, apoLp-II/I, and apoB formed a monophyletic branch (NJ: 81; ML: 78; BI: 1.00), with two subclusters defined by highly significant internal branches, corresponding to crustacean decapod apoCr and insect apoLp-II/I subfamilies (NJ: 87; ML: 80; BI: 1.00) on one side and to vertebrate apoB subfamily (NJ: 100; ML: 100; BI: 1.00) on the other side. Internal sub-branching of the apoCr/apoLp-II/I subcluster was consistent with the species tree, i.e. crustacean and insect sequences were separated in two groups, each one with high support values (NJ: 100; ML: 100; BI: 0.98 and NJ: 100; ML: 100; BI: 1.00, respectively). Duplicated *Metapenaeus ensis *apoCr sequences were clustered together and therefore both belonged to the apoCr subfamily. The second group of sequences named the Vtg/CP family supported by a BI posterior probability of 0.82 grouped with confidence in separate clusters: vertebrate, mollusk, nematode, and insect Vtg, and crustacean CP (NJ: ≥ 99; ML: 100; BI: ≥ 96).

With regard to other crustacean sequences, one point must be highlighted. Vtg from *Daphnia magna*, a species belonging to the class of *Branchiopoda*, and CP from decapod crustaceans, belonging to the class of *Malacostraca*, tend to be grouped with insect Vtg (NJ: 85; ML: 55; BI: 1.00). It should be noted that *Daphnia magna *Vtg is fused with a superoxide dismutase module at the N-terminal end [40]. Therefore, these crustacean proteins are more closely related to *Arthropoda *Vtg than decapod apoCr. Vtg, CP, apoCr, and apoLp-II/I members contain a VWD domain at their C-terminal end (Figure 1) that may be implicated in the clotting cascade and multimerization, as demonstrated with human vWF [41,42]. The functional significance of the VWD domain in Vtg requires additional elucidation. Recent data demonstrated that teleost fish Vtg exhibits an agglutinin activity and may be involved in defense reactions [43,44], and also that insect apoLp-II/I is involved in the immune response [45,46], suggesting a new meaning for the conservation of this domain during evolution. The fact that the phylogenies presented in Figures 2 and 3 were performed using sequences of the LLT module/LPD-N domain reinforces the hypothetical dual-functional nature of crustacean CP. This constitutes the only protein fraction of crustacean VHDL, and its involvement in lipid transport was suggested in earlier studies [31,47,48]. It should be noted that, in crustaceans, the most prominent lipoprotein in the hemolymph, LP1 or BGBP, is not a member of the LLTP superfamily but is also associated with defense reactions [49], supporting a link between lipid transport and immune systems in these animals.

It has been demonstrated that the LPD-N domain found in LLTP forms a "lipid pocket", enabling lipid loading of Vtg [50-53]. By comparative analysis, it was suggested that, in apoB, this domain may similarly form a lipovitellin-like "proteolipid" intermediate containing a lipid pocket that requires MTP for assembly [54]. The presence of apoCr and CP, the latter structurally related to Vtg, in the same shrimp species (*Penaeus monodon*), and of both apoLp-II/I and Vtg in honey bees (*Apis mellifera*), from separate clusters according to our phylogenetic analysis (Figures 2 and 3), strongly suggest that these sequences diverged after an ancient duplication event leading to the separation of an APO paralogous group from Vtg/CP sequences. Through their LLT module/LPD-N domain, these homologous sequences retained the capacity to bind and transport lipids, a function shared with MTP family members that is probably the ancestral function of LLTP [15,55]. The use of truncated forms of apoB in cell cultures showed that the percentage of lipid associated with apoB-100 truncated near the C-terminal end of the LPD-N domain, approached zero. Furthermore, there appeared to be a threshold in apoB size, between 1050 and 1250 amino acids from the N-terminal end, below which the polypeptide may not form a lipoprotein particle [reviewed in ref. [56]]. However, apoB needs to reach a minimum critical length of 884 amino acids for lipoprotein assembly, an MTP-dependent process relying on a portion of apoB located between amino acids 834 and 1134 [57]. Interestingly, apoCr, apoLp-II/I and apoB sequences were found to have a common DUF1081 domain, of unknown function (Figure 1). Protein database screening revealed that this domain was specifically associated with APO sequences (data not shown), and spanned amino acids 957–1072 of human apoB. *Drosophila *MTP promotes the assembly and secretion of human apoB-41 [58], and human MTP enhances the secretion of *Xenopus laevis *Vtg [55], which lacks the DUF1081 domain. A mutant form of human MTP was still able to promote *Xenopus *Vtg synthesis, whereas secretion of human apoB was abolished, suggesting that requirement of apoB for interacting with MTP is more stringent than that of Vtg. This may be related to the presence of the DUF1081 domain in apoB. This domain may be acquired in the APO ancestor through domain accretion and neofunctionalization potentially resulting in better biogenesis of neutral lipid-rich lipoproteins.

Gene duplication facilitates functional divergence but functional constraints enable the retention of genes with overlapping or redundant functions. In addition to their role in plasma and hemolymph lipid transport between somatic tissues, extracellular LLTP, mainly Vtg and apoCr, facilitate the massive deposition of yolk reserves inside the oocytes of most oviparous species. ApoB, apoLp-II/I, and possibly apoCr containing lipoproteins are involved in neutral lipid deposition in the oocyte after receptor-mediated endocytosis on the same or similar oocyte-specific receptor used by Vtg, which belongs to the LDL receptor superfamily [59-61]. Ovarian lipolysis of these circulating lipoproteins may also be a main source of lipids for the growing oocyte [reviewed in ref. [62]]. The low lipid load of crustacean CP and its abundance in shrimp hemolymph cannot fully account for the amount of lipid accumulated within the oocytes [63] and may explain a major role of apoCr in this process in conjunction with the BGBP/LP1.

Relations among the Arthropoda subphyla and the major groups of crustaceans are still a matter for debate. Crustacea exhibit extensive variability in body plans, compared to other arthropod groups and monophyly of crustaceans and relationships among the constituent lineages are controversial [64]. While mitochondrial genomes suggest that hexapods and crustaceans are mutually paraphyletic [65], molecular analyses on rRNA and protein-coding nuclear gene sequences indicate that crustaceans and hexapods form a clade (Pancrustacea or Tetraconata) and that the sister group of Hexapoda is Branchiopoda (fairy shrimps, tadpole shrimps, etc.), rather than Malacostraca (lobsters, crabs, true shrimps, isopods, etc.) [66,67], thereby making hexapods terrestrial crustaceans and the traditionally defined Crustacea, paraphyletic. Additional Branchiopoda and non Decapoda apoCr and Vtg/CP sequences, together with their role as a main egg yolk precursor protein, may help to clarify the Pancrustacea-major splits, e.g. provide support for the branchiopods being closest to hexapods and reject the alternative, e.g. malacostracan-hexapod association. Additional data will also clarify the relationships among the many malacostracan subgroups [68].

## Conclusion

Observation of modular architecture and phylogenetic analyses demonstrated that the main egg yolk precursor protein of decapod crustaceans is a member of the LLTP superfamily, is homologous to insect apoLp-II/I and vertebrate apoB, and would be more appropriately called apolipocrustacein (apoCr) rather than Vtg. The presence of apoCr and a clotting protein structurally related to Vtg from the same shrimp species (e.g. *Penaeus monodon*) and both apoLp-II/I and Vtg from insect species (e.g. *Apis mellifera*), suggest that, in addition to their involvement in the lipid metabolism, extracellular LLTP may have acquired other functions during metazoan evolution, e.g. involvement in clotting cascade and immune response. The findings of this study may be also a starting point for using LLTP phylogeny and their functional role to clarify the controversial relationships among Pancrustacean constituent lineages.

## Methods

### Data sets

Accession numbers for LLTP sequences used from GenBank™/EBI or UniProt databases are GenBank:AY051318 (*Penaeus semisulcatus*), GenBank:AB033719 (*Marsupenaeus japonicus*), GenBank:AY103478 (Vg1) and GenBank:AY530205 (Vg2) (*Metapenaeus ensis*), GenBank:AY321153 (*Litopenaeus vannamei*), GenBank:AY499620 (*Fenneropenaeus merguiensis*), and GenBank:DQ288843 (*Penaeus monodon*) for *Crustacea Decapoda Penaeidae *apoCr sequences; GenBank:AB117524 (*Pandalus hypsinotus*), GenBank:AF306784 (*Cherax quadricarinatus*), GenBank:AB056458 (*Macrobrachium rosenbergii*), GenBank:AY724676 (*Charybdis feriatus*), and GenBank:AAX94762 (*Portunus trituberculatus*) for *Crustacea Decapoda Pleocyemata *apoCr sequences; UniProt:Q9U943 for locust (*Locusta migratoria*), UniProt:Q25490 for tobacco hornworm (*Manduca sexta*), UniProt:Q9V496 for fruit fly (*Drosophila melanogaster*), GenBank:XP_392490 for honey bee (*Apis mellifera*), GenBank:XP_321226 for African malaria mosquito (*Anopheles gambiae*), and GenBank:BAB32641 for silk moth (*Samia cynthia ricini*) apoLp-II/I sequences; GenBank:XP_694827 for zebrafish (*Danio rerio*), GenBank:XP_419979 for chicken (*Gallus gallus*), and UniProt:X04714 for human (*Homo sapiens*) apoB sequences; GenBank:AAD16454 for freshwater crayfish (*Pacifastacus leniusculus*), and GenBank:AAF19002 for shrimp (*Penaeus monodon*) CP sequences; GenBank:NP_610075 for fruit fly (*Drosophila melanogaster*), GenBank:XP_319421 for African malaria mosquito (*Anopheles gambiae*), GenBank:AAR27937 for nematode (*Caenorhabditis elegans*), GenBank:XP_788526 for sea urchin (*Strongylocentrotus purpuratusthe*), UniProt:Q8AXV7 for zebrafish (*Danio rerio*), UniProt:X78567 for bovine (*Bos taurus*), and UniProt:P55157 for human (*Homo sapiens*) large subunit of MTP sequences; GenBank:BAC22716 for oyster (*Crassostrea gigas*), GenBank:BAB63260 (partial sequence) for Yesso scallop (*Mizuhopecten yessoensis*), UniProt:P55155 (Vtg2), UniProt:P06125 (Vtg5), UniProt:P18948 (Vtg6) for nematode (*Caenorhabditis elegans*), UniProt:T18561 (Vtg6) for nematode (*Oscheius *sp., CW1 strain), UniProt:Q91062 for lamprey (*Ichthyomyzon unicuspis*), UniProt:Q90243 for sturgeon (*Acipenser transmontanus*), UniProt:U07055 for mummichog (*Fundulus heteroclitus*), UniProt:Q92093 for rainbow trout (*Oncorhynchus mykiss*), UniProt:P18709 for *Xenopus *(*Xenopus laevis*), and UniProt:P87498 for chicken (*Gallus gallus*) (Vtg1) Vtg sequences. As previously demonstrated, using the NJ method [69], insect Vtg sequences available in databases clustered with high confidence in a group separated from other Vtg sequences, including those of crustaceans apoCr. Vtg sequences representative of five insect orders (Lepidoptera, Diptera, Coleoptera, Hemiptera, and Hymenoptera) were therefore selected for analysis and were: UniProt:Q27309 for silkworm (*Bombyx mori*), UniProt:U60186 for gypsy moth (*Lymantria dispar*), UniProt:U02548 for yellow fever mosquito (*Aedes aegypti*), UniProt:Q05808 for boll weevil (*Anthonomus grandis*), UniProt:U97277 for bean bug (*Riptortus clavatus*), GenBank:NP_001011578 for honey bee (*Apis mellifera*), and GenBank:AF026789 for parasitoid wasp (*Pimpla nipponica*).

### Sequence analyses

The deduced amino acid sequence of *Penaeus semisulcatus *[23] was subjected to a BLASTP search using the Blosum 62 matrix [70]. Amino acid sequences corresponding to the best hits were then aligned using Clustal X software, version 1.8 [71]. Sequence alignments were edited and manually corrected with GeneDoc software [72]. The twenty-two N-terminal conserved amino acid sequence motifs (N1 to N22) of the LLT module were extracted from selected LLTP and aligned as previously described [15] resulting in a concatenated sequence 351 amino acid long. Domain architecture of LLTP was examined and compared using CDART [73].

The phylogenetic tree and branch support values were estimated using three different methodologies of phylogenetic reconstruction: 1) NJ, 2) ML and 3) BI. NJ algorithm was based on the number of amino acid substitutions per site with the Poisson-correction distance method and pairwise-deletion option for gap sites, and bootstrap support values were obtained with 5,000 pseudoreplicates. The distance analysis was carried out with MEGA 3.1 [74]. ML analyses was carried out with PHYML v2.4.4 [75] starting from the BIONJ tree, and the gamma distribution for rate heterogeneity across sites (Γ) was modeled with a four-category Γ distribution and a shape parameter equal to 2. The WAG substitution model [76] was selected by ProtTest v1.3 [77], following the Akaike information criterion, as best-fitting model among the models tested that could be used in PHYML. Bootstrap values were based on 500 pseudoreplicates to estimate support for the nodes of the ML tree. A graphical representation of the ML phylogeny was generated with MEGA 3.1 tree explorer. In the non-parametric-bootstrap trees, bootstrap confidence level values ≥75 were accepted as significant. BI was performed using MrBayes v3.1.2 [78] with the WAG model of amino acid substitution provided in the package. Two simultaneous runs each with four simultaneous Markov Chain Monte Carlo (MCMC) chains run initially for 1,000,000 generations, after which the average standard deviation of split frequencies was 0.004614, saving the current tree to file every 100 generations for a total of 10,000 trees in the initial sample. Default cold and heated chain parameters were used. MCMC runs were summarized and further investigated for convergence of all parameters, using the *sump *and *sumt *commands in MrBayes and the computer program Tracer version 3.1 [79]. Stationarity was determined to have occurred by the 100,000th generation. Accordingly, the first 1000 trees prior to log likelihood stabilization were discarded (as burn-in), and the following 9,000 tree samples were used to estimate topology and tree parameters. The percentage of times a node occurred within those 9,000 trees was interpreted as the posterior probability of the node. A graphical representation of the majority rule consensus tree was generated with TreeView v1.6.6 program [80]. In the Bayesian tree, clades were accepted as significant at ≥0.80 posterior probability.

## Abbreviations

apoB, apolipoprotein B; apoCr, apolipocrustacein; apoLp-II/I, apolipophorin II/I; APO, apolipocrustacein/apolipophorin/apolipoprotein monophyletic group; BI, Bayesian inference; CP, clotting protein; LLT, large lipid transfer; LLTP, large lipid transfer proteins; MCMC, Markov Chain Monte Carlo; ML, maximum likelihood; MTP, large subunit of microsomal triglyceride transfer protein; neighbor-joining, NJ; very high density lipoprotein, (VHDL); Vtg, vitellogenin; VWD, von Willebrand factor type D; vWF, von Willebrand factor.

## Authors' contributions

J-C.A, E.L and P.J.B. designed research; J-C.A. and P.J.B performed research; J-C.A., E.L. and P.J.B analyzed data; and J-C.A., E. L. and P.J.B wrote the paper. All authors read and approved the final manuscript.

## Supplementary Material

Additional File 1 **Alignment of deduced amino acid sequences from crustacean apolipocrustaceins (apoCr) and insect apolipophorins II/I (apoLp-II/I)**. The conservative substitutions allowed were colored and defined as follows: A, G; S, T; E, D; R, K, H; Q, N; V, I, L, M; Y, F, W; P; and C. Gaps inserted to optimize alignments are indicated by dashes. Sites of identical or conserved amino acids in all sequences are highlighted in red and gray, respectively. The conserved functional subtilisin-like endoprotease cleavage site is highlighted in yellow. The name of each domain and of each conserved motif, from N1 to N22, of the LLT module [15] is indicated above and below the alignments, respectively. Click here for file

Additional File 2 **Alignment of LLT module conserved protein sequence motifs extracted from LLTP superfamily members**. The conservative substitutions allowed were colored and defined as in Additional file 1. Gaps inserted to optimize alignments are indicated by dashes. Single dots indicate missing data. The site of identical conserved amino acids in all sequences is highlighted in red. The name of each conserved motif, from N1 to N22, of the LLT module [15] is indicated below the alignments. Click here for file
