# Supplementary material for: Apolipocrustacein, formerly vitellogenin, is the major egg yolk precursor protein in decapod crustaceans and is homologous to insect apolipophorin II/I and vertebrate apolipoprotein B
Source: BMC Evol Biol. 2007 Jan 22;7:3. doi: 10.1186/1471-2148-7-3 (PMC1783640; doi:10.1186/1471-2148-7-3)
Supplement: Additional File 1 — Alignment of deduced amino acid sequences from crustacean apolipocrustaceins (apoCr) and insect apolipophorins II/I (apoLp-II/I). The conservative substitutions allowed were colored and defined as follows: A, G; S, T; E, D; R, K, H; Q, N; V, I, L, M; Y, F, W; P; and C. Gaps inserted to optimize alignments are indicated by dashes. Sites of identical or conserved amino acids in all sequences are highlighted in red and gray, respectively. The conserved functional subtilisin-like endoprotease cleavage site is highlighted in yellow. The name of each domain and of each conserved motif, from N1 to N22, of the LLT module [15] is indicated above and below the alignments, respectively. [file 1471-2148-7-3-S1.pdf]

```
P. semisulcatus apoCr        -MTTSNILFV LAFVAG---- ---------- ---------- ---------G LAAPWGADLP RCSTECPITG SPKLAYQPDK  46
F. merguiensis apoCr         -MTTSTLLFV LAFVAG---- ---------- ---------- ---------G LAAPWGADVP RCSTECPVTG SPKLAYQPDK  46
L. vannamei apoCr            ---------- ---------- ---------- ---------- ---------- ---------- -FSTECPITG SPKLAYQPDK  19
P. monodon apoCr             -MTTSTLLFI LAFVTG---- ---------- ---------- ---------G LAAPWGADLP RCSTECPITG SPKLAYQPDK  46
M. japonicus apoCr           -MTTSSLLFV LALVAG---- ---------- ---------- ---------G LAAPWGADLP RCSTECPISG SPKLAYQPEK  46
M. ensis apoCr1              -MNSSSLVLV LALVAG---- ---------- ---------- ---------G LAAPREDEAP RCSTECPVTG SPKLAYEPGK  46
M. ensis apoCr2              -MNSSSLVLV LALVAG---- ---------- ---------- ---------G LAAPWGAEVP RCSTECPVTG SPKLAYEPGK  46
C. quadricarinatus apoCr     -MTTSAALIV LPLVAG---- ---------- ---------- ---------A GAPPFGGNTP VCSTECPIAG SPKLFYQPGK  46
M. rosenbergii apoCr         --MTSSVLLA FVILAT---- ---------- ---------- ---------A SAAPWPSGTN LCSKECPVAG SPKLFYAPEK  45
P. hypsinotus apoCr          -MTSSTALFV LGFLAA---- ---------- ---------- ---------A SAAPWPSNLP RCSTECPIAG SPKLDYAPEK  46
P. trituberculatus apoCr     -MTTHT-VLL LALTAA---- ---------- ---------- ---------A VAAPYGGTTQ LCSTECPLAA A-KLSFIPGK  45
C. feriatus apoCr            -MTTHT-VLL LALAAA---- ---------- ---------- ---------A AAAPYGSTIQ LCSTECPVAA A-KLAFTPGK  44
D. melanogaster apoLpII/I    MARMKYNIAL IGILAS---- ---------- ---------- ---------V LLTIAVNAEN ACNLGCPKSD NGLLKYIPGN  47
A. mellifera apoLpII/I       -MTINTISPK FSIPSSNHFH CVLERAPQGA PQGVLHLIRY RFPRHSSSWS FQSAFHNPRP TCVTGCRGMH Q-SKAYQEGR  78
L. migratoria apoLpII/I      -MGTPPHIWF LLILAIS--- ---------- ---------- ---------- -SGGLSAAVG GCNHQCTPQS S-VFQYQKGQ  44
A. gambiae apoLpII/I         ---------- ---------- ---------- ---------- ---------- ---------- --------NK SSKYTFKPGF  12
M. sexta apoLpII/I           -MGKSNRLLS VLFVIS---- ---------- ---------- ---------- VLWKAAYGNG KCQIACKGSS S--PSFAAGQ  43
```

LPD-N domain

```
P. semisulcatus apoCr        TYAYAYSGKS TVQLKGVNNG ---DAETEWT AGVDLTWISP CDMAISFRNT KIDGARG--P IVARALERHP LVVAVVDGRV  121
F. merguiensis apoCr         TYAYAYSGKS TVQLKGVDNG ---DTETEWT AGVDLTWISP CDMAISFRNT KMDGARG--P TAARTLERYP LVVAVVDGRV  121
L. vannamei apoCr            TYAYQYSGKS KVQLKGVDNG ---DSETEWT AQVDLTWISP CDMAISFKNT KVDGTPG--P IVARTLERHP LVVAVVDGRV  94
P. monodon apoCr             TYAPPYSGKS RVHLKGVDNG ---DSEIEWT AGVDLTWISP CDMAISFRNT KMDGARG--P IAASTLERHP LVVAVVDGRV  121
M. japonicus apoCr           TYTYQYSGKS RVQLKGVDDG ---VSETEWA ARVDLTWISP CDVAISFNNM KMDGARG--P IAARTLERHP LVVAVVDGRV  121
M. ensis apoCr1              TYTYAYSGKS KVQLKGVQDG ---DSDIEWA AGIDLTWITP CDVAISFTNI KMDGAPGK-P DAARTLQRFP MVVAVAEGRV  122
M. ensis apoCr2              TYTYAYSGKS EVQLKGVQDG ---VTDLEWN KHVELTWITP CDVGISVKNT EVDGVTG--P DMKKYLERYP LVVAVVDGRV  121
C. quadricarinatus apoCr     TYTYEYSGKS RIQLKGVEGG ---LTETDWS AQVELSWITP CDMVITMKDS KTDGATV--P GASRFLERYP LVVAVTDGKV  121
M. rosenbergii apoCr         TYVYSYTGKS RTHLRDVEGA ---TAEMEWN SQVELSWLSP CDMAITMKNP SIGGGSG--S AEARFLERYP LVVSIIDGRV  120
P. hypsinotus apoCr          TYVYAYSGKS RIQMKDVEGG ---NADMEWT SQVELTWLSR CDMAISIKNP SIGGGAG--S PEAKLLEKYP LVVAMIDGRV  121
P. trituberculatus apoCr     TYSYTYSGKS IVQLKGVDGG ---LVETQWE KQMLLTVLGP CDVAISFKGT KVDGKTG--L PGSDKLERYP LVVAMTDGRV  120
C. feriatus apoCr            TYSYTYTGKS QVQLKGVDGG ---VVDTRWE EQVLLTVLGP CDLAISFKGS KVDGKTG--L SGSDKLERYP LVVAMTDGRV  119
D. melanogaster apoLpII/I    YYDYSFDSIL TIGASSDVPN DSDDTSLKVS GSAKIFAKGN CGYTLQLSSV KVTNTKESVE KKILNSIQKP VQFTLVSGIL  127
A. mellifera apoLpII/I       TYVYNLEGLS VTSITDAQG- ---DASLKLS ATVELSVKPD CINQLRLKNV KINGAPP--- -LIPEIEQYA VQFNYHDGHI  150
L. migratoria apoLpII/I      TYTYSFEGTT LTSLPGTQGE ---PVRLKLK ATADLSVADD CNKVLRLRGV TVSGPDSKNY ANLKDLEAHP VLANFKGSSI  121
A. gambiae apoLpII/I         VYQYDVDSYV QLQQSDKENK ---QTTLKVD GKVEVYAGDN CQYTLKVVSL TSYAPDGK-K TAFGADISKP VQFTLSNDEL  88
M. sexta apoLpII/I           KYNYGVEGTV SVYLTGADNQ ---ETSLKML GQASVSAISN CELELSVHNM VLSGPDGK-K YPCPQGIEKP VRFSYQDGRV  119
```

N1 N2

LPD-N domain

```
P. semisulcatus apoCr        QH-VCAHPED EPWAINLKKG VASAFQNSIP SLSAVSSGIT VTETDVVGKC PTKYEIESEG EKVIVVKEKN HRHCQERYPT  200
F. merguiensis apoCr         QH-VCAHPED EAWAINLKKG VASAFQNSIP SLSAVSSGIT VTETDVVGKC PTKYEIETEG EKVIVVKEKN HRHCQERYPT  200
L. vannamei apoCr            QH-VCAHPED EPWAINLKKG VASAFQNSIP SLSAVSSGIT VTETDVVGKC PTKYEIETEG EKVIVVKEKN HRHCQQRYLT  173
P. monodon apoCr             QH-VCAHPED EPWAINMKKG VASAFQNSIP SLSTVSSGIT VTETDVVGKC PTKYEIKTEG EKVIVVKEKN HRHCQERYPT  200
M. japonicus apoCr           QH-VCAHPDD EPWAINLKKG VASAFQNSIP SLSTVSSGMT VTETDVVGKC PTTYQIETEG EKVIVVKEKN HRHCQQRYPT  200
M. ensis apoCr1              QH-VCAHPED QPWAINLKKG VASALQNSIP SLSPVASGIT VTETDVIGKC PPNTKVKTEG RELIVTKEKE HRHCTEHFPT  201
M. ensis apoCr2              EH-VCAHPDD APWSVNMKKG IASIIQNSLP SLSPLSSGLT LTETDVVGKC PTKYEVEPEG EKLIVTKEKN HRHCHERFPT  200
C. quadricarinatus apoCr     HH-VCSHPDD DTWSINLKKG VASAFQNSLP SNSTINSGQN ITETDVVGKC PTRYEVQDHG DTVIMIKEKN HRQCKERYHT  200
M. rosenbergii apoCr         KA-ACSHPDD DTWSINLKKG IASAFQNTLP SNSTINSGLN FTETDIIGNC STMYEVQHEG EKVVVTKLKN HRFCQDLYVN  199
P. hypsinotus apoCr          QQ-ACSHPDD DVWSINMKKG IASAFQNSLP SNSSMNSGLN FTETDIIGNC STRYEVENQG EKVIVTKMKN HRFCQDHIVS  200
P. trituberculatus apoCr     QR-VCSHPDD DTWAINMKKG VVSALQISLP SLSISNSGLN FTETDVLGTC PTYYEVQGEG ARVLVKKEKN HRLCKEHYPT  199
C. feriatus apoCr            QR-VCSHPDD DTWAINMKKG VVSALQISLP SLSISNSGLN FTETDVLGTC PTYYEVQAEG AKVLVKKEKN HRLCQEHYPT  198
D. melanogaster apoLpII/I    EPQICSDSSD LDYSLNIKRA VVSLLQSGIE AEH------- --EVDVFGMC PTHTSTSKVG NANIITKARN LNSCSHREQI  198
A. mellifera apoLpII/I       DTQLCTEPGD SQASLNIKRA VVSMFQSAIM QDS--GS-TI HHETDVMGTC PTEFNFRKEG DSLIVNKNRN LASCAFRENV  227
L. migratoria apoLpII/I      NKQLCSEDGV NQSSLNIKQA ILSLLQTPNT KSS------T VSEVDVFGIC PTNVRHSQRG DVTVISKTRN LNRCASRENL  195
A. gambiae apoLpII/I         LPEICTEADD TDFSLNVKRG LISLFQVAQE KST------- --ETDVFGVC QTSFSSYPSG DATVVEKVRD LGNCAYRESL  159
M. sexta apoLpII/I           GPEICAAEDD SRRSLNIKRA IISLLQAEQK PS-------- -VQVDVFGVC PTEVSSSQEG GAVLLHRSRD LSRCAHREQG  190
```

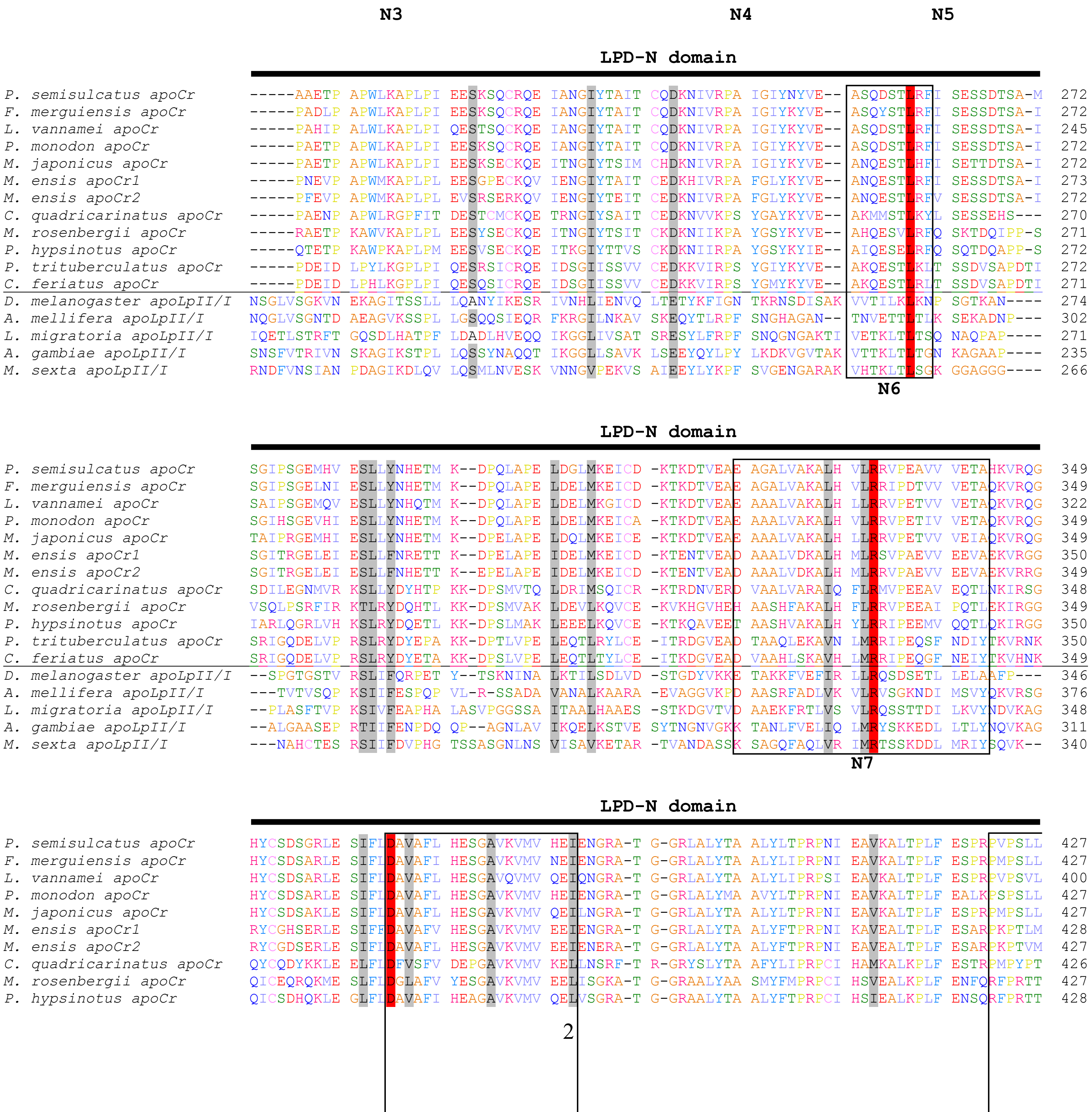

N3
N4
N5
LPD-N domain
P. semisulcatus apoCr -----AAETP APWLKAPLPI EESKSQCRQE IANGIYTAIT CQDKNIVRPA IGIYNYVE-- ASQDSTLRFI SESSDTSA-M 272
F. merguiensis apoCr -----PADLP APWLKAPLPI EESKSQCRQE IANGIYTAIT CQDKNIVRPA IGIYKYVE-- ASQYSTLRFI SESSDTSA-I 272
L. vannamei apoCr -----PAHIP ALWLKAPLPI QESTSQCKQE IANGIYTAIT CQDKNIVRPA IGIYKYVE-- ASQDSTLRFI SESSDTSA-I 245
P. monodon apoCr -----PAETP APWLKAPLPI EESKSQCRQE IANGIYTAIT CQDKNIVRPA IGIYKYVE-- ASQDSTLRFI SESSDTSA-I 272
M. japonicus apoCr -----PAETP APWLKAPLPI EESKSECKQE ITNGIYTSIM CHDKNIVRPA IGIYKYVE-- ANQESTLHFI SETTDTSA-I 272
M. ensis apoCr1 -----PNEVP APWMKAPLPL EESGPECKQV IENGIYTAIT CEDKHIVRPA FGLYKYVE-- ANQESTLRFI SESSDTSA-I 273
M. ensis apoCr2 -----PFEVP APWMKAPLPL EVSRSERKQV IENGIYTEIT CEDKNIVRPA FGLYKYVE-- ANQESTLRFV SESSDTSA-I 272
C. quadricarinatus apoCr -----PAENP APWLRGPFIT DESTCMCKQE TRNGIYSAIT CEDKNVVKPS YGAYKYVE-- AKMMSTLKYL SESSEHS--- 270
M. rosenbergii apoCr -----RAETP KAWVKAPLPL EESYSECKQE ITNGIYTSIT CKDKNIIKPA YGSYKYVE-- AHQESVLRFQ SKTDQIPP-S 271
P. hypsinotus apoCr -----QTETP KAWPKAPLPM EESVSECKQE ITKGIYTTVS CKDKNIIRPA YGSYKYIE-- AIQESELRFQ SQTDQAPP-S 272
P. trituberculatus apoCr -----PDEID LPYLKGPLPI QESRSICRQE IDSGIISSVV CEDKKVIRPS YGIYKYVE-- AKQESTLKLT SSDVSAPDTI 272
C. feriatus apoCr -----PDEID LPHLKGPLPI QESQSICRQE IDSGIISSVV CEDKKVIRPS YGMYKYVE-- AKQESTLRLT SSDVSAPDTI 271
D. melanogaster apoLpII/I NSGLVSGKVN EKAGITSSLL LQANYIKESR IVNHLIENVQ LTETYKFIGN TKRNSDISAK VVTILKLKNP SGTKAN---- 274
A. mellifera apoLpII/I NQGLVSGNTD AEAGVKSSPL LGSQQSIEQR FKRGILNKAV SKEQYTLRPF SNGHAGAN-- TNVETTLTLK SEKADNP--- 302
L. migratoria apoLpII/I IQETLSTRFT GQSDLHATPF LDADLHVEQQ IKGGLIVSAT SRESYLFRPF SNQGNGAKTI VETKLTLTSQ NAQPAP---- 271
A. gambiae apoLpII/I SNSFVTRIVN SKAGIKSTPL LQSSYNAQQT IKGGLLSAVK LSEEYQYLPY LKDKVGVTAK VTTKLTLTGN KAGAAP---- 235
M. sexta apoLpII/I RNDFVNSIAN PDAGIKDLQV LQSMLNVESK VNNGVPEKVS AIEEYLYKPF SVGENGARAK VHTKLTLSGK GGAGGG---- 266
N6
LPD-N domain
P. semisulcatus apoCr SGIPSGEMHV ESLLYNHETM K--DPQLAPE LDGLMKEICD -KTKDTVEAE AGALVAKALH VLRRVPEAVV VETAHKVRQG 349
F. merguiensis apoCr SGIPSGELNI ESLLYNHETM K--DPQLAPE LDELMKEICD -KTKDTVEAE AGALVAKALH VLRRIPDTVV VETAQKVRQG 349
L. vannamei apoCr SAIPSGEMQV ESLLYNHQTM K--DPQLAPE LDELMKGICD -KTKDTVEAE AAALVAKALH LLRRVPETVV VETAQKVRQG 322
P. monodon apoCr SGIHSGEVHI ESLLYNHETM K--DPQLAPE LDELMKEICA -KTKDTVEAE AAALVAKALH VLRRVPETIV VETAQKVRQG 349
M. japonicus apoCr TAIPRGEMHI ESLLYNHETM K--DPELAPE LDQLMKEICE -KTKDTVEAE AAALVAKALH LLRRVPETVV VEIAQKVRQG 349
M. ensis apoCr1 SGITRGELEI ESLLFNRETT K--DPELAPE IDELMKEICE -KTENTVEAD AAALVDKALH MLRSVPAEVV EEVAEKVRGG 350
M. ensis apoCr2 SGITRGELEI ESLLFNHETT K--EPELAPE IDELMKEICD -KTENTVEAD AAALVDKALH MLRRVPAEVV EEVAEKVRRG 349
C. quadricarinatus apoCr SDILEGNMVR KSLLYDYHTP KK-DPSMVTQ LDRIMSQICR -KTRDNVERD VAALVARAIQ FLRMVPEEAV EQTLNKIRSG 348
M. rosenbergii apoCr VSQLPSRFIR KTLRYDQHTL KK-DPSMVAK LDEVLKQVCE -KVKHGVHEH AASHFAKALH FLRRVPEEAI PQTLEKIRGG 349
P. hypsinotus apoCr IARLQGRLVH KSLRYDQETL KK-DPSLMAK LEEELKQVCE -KTKQAVEET AASHVAKALH YLRRIPEEMV QQTLQKIRGG 350
P. trituberculatus apoCr SRIGQDELVP RSLRYDYEPA KK-DPTLVPE LEQTLRYLCE -ITRDGVEAD TAAQLEKAVN LMRRIPEQSF NDIYTKVRNK 350
C. feriatus apoCr SRIGQDELVP RSLRYDYETA KK-DPSLVPE LEQTLTYLCE -ITKDGVEAD VAAHLSKAVH LMRRIPEQGF NEIYTKVHNK 349
D. melanogaster apoLpII/I --SPGTGSTV RSLIFQRPET Y--TSKNINA LKTILSDLVD -STGDYVKKE TAKKFVEFIR LLRQSDSETL LELAAFP--- 346
A. mellifera apoLpII/I ---TVTVSQP KSIIFESPQP VL-R-SSADA VANALKAARA -EVAGGVKPD AASRFADLVK VLRVSGKNDI MSVYQKVRSG 376
L. migratoria apoLpII/I --PLASFTVP KSIVFEAPHA LASVPGGSSA ITAALHAAES -STKDGVTVD AAEKFRTLVS VLRQSSTTDI LKVYNDVKAG 348
A. gambiae apoLpII/I --ALGAASEP RTIIFENPDQ QP--AGNLAV IKQELKSTVE SYTNGNVGKK TANLFVELIQ LMRYSKKEDL LTLYNQVKAG 311
M. sexta apoLpII/I ---NAHCTES RSIIFDVPHG TSSASGNLNS VISAVKETAR -TVANDASSK SAGQFAQLVR IMRTSSKDDL MRIYSQVK-- 340
N7
LPD-N domain
P. semisulcatus apoCr HYCSDSGRLE SIFLDAVAFL HESGAVKVMV HEIENGRA-T G-GRLALYTA ALYLTPRPNI EAVKALTPLF ESPRPVPSLL 427
F. merguiensis apoCr HYCSDSARLE SIFLDAVAFI HESGAVKVMV NEIENGRA-T G-GRLALYTA ALYLIPRPNI EAVKALTPLF ESPRPMPSLL 427
L. vannamei apoCr HYCSDSARLE SIFLDAVAFL HESGAVQVMV QEIQNGRA-T G-GRLALYTA ALYLIPRPSI EAVKALTPLF ESPRPVPSVL 400
P. monodon apoCr HYCSDSARLE SIFLDAVAFL HESGAVKVMV HEIENGRA-T G-GRLALYMA AVYLTPRPNI EAVKALTPLF EALKPSPSLL 427
M. japonicus apoCr HYCSDSAKLE SIFLDAVAFL HESGAVKVMV QEILNGRA-T G-GRLALYTA ALYLTPRPNI EAVKALTPLF ESPRPMPSLL 427
M. ensis apoCr1 RYCGHSERLE SIFFDAVAFV HESGAVKVMV EEIENGRA-T G-GRLALYTA ALYFTPRPNI KAVEALTPLF ESARPKPTLM 428
M. ensis apoCr2 RYCGDSERLE SIFLDAVAFL HESGAVKVMV EEIENERA-T G-GRLALYTA ALYFTPRPNI EAVEALTPLF ESARPKPTVM 427
C. quadricarinatus apoCr QYCQDYKKLE ELFLDFVSFV DEPGAVKVMV KELLNSRF-T R-GRYSLYTA AFYLIPRPCI HAMKALKPLF ESTRPMPYPT 426
M. rosenbergii apoCr QICEQRQKME SLFLDGLAFV YESGAVKVMV EELISGKA-T G-GRAALYAA SMYFMPRPCI HSVEALKPLF ENFQRFPRTT 427
P. hypsinotus apoCr QICSDHQKLE GLFLDAVAFI HEAGAVKVMV QELVSGRA-T G-GRAALYTA ALYFTPRPCI HSIEALKPLF ENSQRFPRTT 428
2

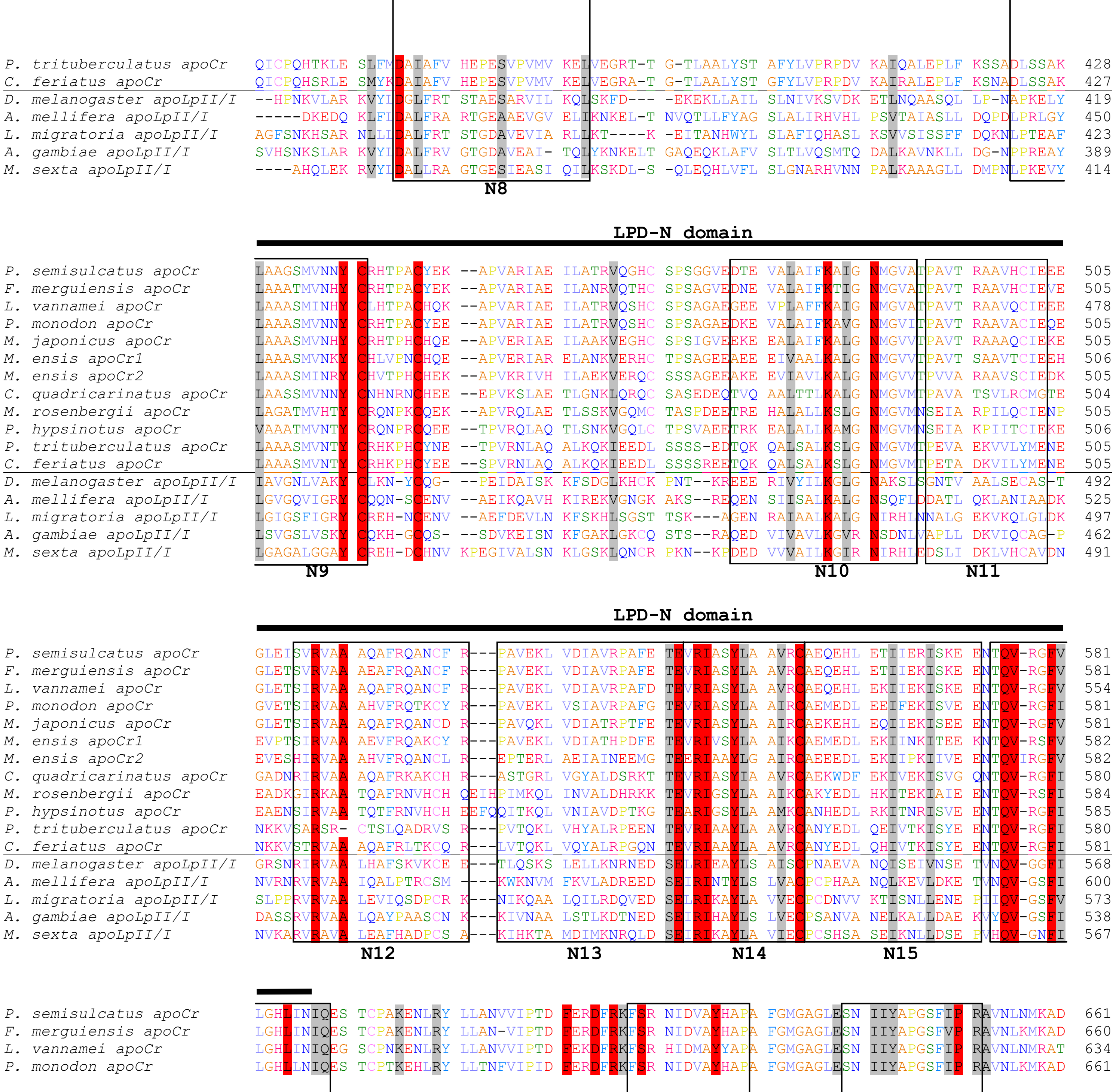

N8
LPD-N domain
N9
N10
N11
LPD-N domain
N12
N13
N14
N15

```
M. japonicus apoCr           LGHLINIQES TCPTKENLKY LLTNVVIPTD FEKDFRKFSR NVEMSYHAPA FGMGADLESN IIYAPGSFIP RAVNLNMKAA  661
M. ensis apoCr1              LGHLINIQES TCPSKEHLRY LLTDFVIPTD FERDFRRVSR NVMMGYHSAA FEMGADVESN IIYAPGAFVP RALTMNLKAD  662
M. ensis apoCr2              LSHLLNIQES ASPDRERLRY LLSNFVIPRD FDGDIRKHSR NVEMSYFAPS LGLGAGIESN IIYTPESFLP RAIDFNLRTI  662
C. quadricarinatus apoCr     LSHLRNVQQS DAPDKENLRN LLTNIVIPRN FKTDIRKYSR NLDLSYFSPS AGVGAGLESN IIYAPGSFIP RSIDFNLTAA  660
M. rosenbergii apoCr         LSHLHNIKES TVPYKANLKS RLETIVLPSN FTKDWRKHSR NIDMSYYAPT FGVGAGVESN VIYAPGSYVP RSVNLNLTAA  664
P. hypsinotus apoCr          LSHLLNIQES TTPHKEHLKY LLTTIVLPSN FTADWRKYSR NIDLSYYAPT FGVGAGMESN VIYAPGSYVP RSVNLNLTAA  665
P. trituberculatus apoCr     LSHLINLQKS DAPEKQSLRY MMTNIVLPQD FEADIRKYSQ NLDLSYFSES AGVGAEVESN LIYAPGSMIP RSLGVNLTAA  660
C. feriatus apoCr            LSHLINLQQS DAPGKQRLRY LLTNILLPQD FETDIRKYSQ NLDLSYFSPS A---AGLESN MIYAPGSMLP RSLGVNLTAA  658
D. melanogaster apoLpII/I    SSNLKAIRDS TDVSRDQQKY HLANIRVTKT FPVDYRRYSF NNEVSYKLES LGVGASTDYQ IIYSQHGFLP RSSRINVTTE  648
A. mellifera apoLpII/I       QTHLRNLRAS TDPDKLNAKN QFGLIKPRVK FPEDFRKYSF NNELSYKIDS LGLGSTLDSN VVYSQNSFVP RSANLNMTVE  680
L. migratoria apoLpII/I      VSHLKNLQAS TDPSKAEAKE KLGQLKPKKI FSSDIRKYSQ NYELSYAIDA INAGASVESN VIFSQSSYLP RSVSLNLTAD  653
A. gambiae apoLpII/I         TSHLASLRAS VDPTRDAARQ HFGKIRTSNK FPFDVRRYSF NREFSYAVES LGVGASAETS VIYSQKSFLP KSVGLNFTAE  618
M. sexta apoLpII/I           TSSLRHIRSS SNPDKQLAKK HYGQIRTPNK FKVDERKYSF YREMSYKLDA LGAGGSVDQT VIYSQTSFLP RSVNFNLTVD  647
                               N16                                 N17                    N18

P. semisulcatus apoCr        VDGT--HMDI AEIGARFVGI DSIIEELLGP QGYLRRATFG KIMEDITGFA GEKGLKVMEH IKHTLRTRRS IDSSVISDFF  739
F. merguiensis apoCr         VDET--HMDI AEIGARFVGI ESIIEEPLGP QGYLRTATFG KIMEDITGFA GEKGYKVMEQ LKHTLRTRRS IDSSVIADFF  738
L. vannamei apoCr            VDET--PMDI AEIGARFEGV DSIIEELLGP QGYLRKATFG KIMEDITGFA GEKGLKIMEH IKHTMRTRRS IDASVISDFF  712
P. monodon apoCr             VDEM--HMDI AEIGARFEGI DSIIEELLGP QGYLHRATFG KIMEDITGFA GEKGLKVMEH IKHTLRTRRS IDSSVISDFF  739
M. japonicus apoCr           VDET--HMDL AEIGARFEGI DSIIEELFGP EGYLRKATFG KIMQDITGFA EEKGLKVMEH IKQTLRTKRS IDSSVISDFF  739
M. ensis apoCr1              IDET--HLDI GEIGARFEGV DSIIEEHFGP EGYLRKASFG KILEDITGFA EEKGYKVMEH LENTLRTRRS IDASTIADFF  740
M. ensis apoCr2              IENI--PINL GEAGIRFEGL DAIVKELAGP EGYLRKTPLG RILKDLTSVA KEKGAHIAEH LEESFRGRRS ISQSAIRGFL  740
C. quadricarinatus apoCr     LEGI--SMNI GEVGARFEGL DPFIEKLFGP ESYFQKASYK QIFSEMTSLF HEKKNKFLEH FHGDFKHKRS IDMSTLSNFF  738
M. rosenbergii apoCr         LGAT--PFNI GEIGARFEGL ETVVEELFGP HSYFKRTPAR QIWEDLK--- -ETLSKVTER LQGSFRQRRS IDLSQISHLF  738
P. hypsinotus apoCr          LGAT--PFNM GEIGARIEGL EPILEDLFGP EGYMSKTPTS QIFQDISSSM GDKFSKIIER LQGSLRQKRS IDFSSLSHLF  743
P. trituberculatus apoCr     LDGTGIPMNL GEIGARLEGL EPILAQLLGP ASYLKTSSYS KMFNDLVSFI QKNWSTIKQE LEVAIRERRS VDYAALESII  740
C. feriatus apoCr            PDGTGIPINI ADVGARVEGL EPIIAQLLGP ASYLKTSSYS KIFNDLVSFI QQNWSTIKQE LEVAIRERRT VDDATIESII  738
D. melanogaster apoLpII/I    FFGT--NYNV FEASVRQENV EDVLEYYLGP KG-LVNKDFD EIVKLIEVG- -------NNG VAAGGRARRS IVDDVSKISK  717
A. mellifera apoLpII/I       LFGK--NFNF LELNTRVENL DRLLEHYLGP KGKIWEKDLE EDLKSGANEV NKLRKYARER FEKVVRGKRE VRQGDLDRFA  758
L. migratoria apoLpII/I      VFGH--SYNV FEIAARTENL DHIIESFLGP KGYIETEDDD KFVDEVEEKT KSLYNRITER FEKTFRQKRS VSKDAVDNIR  731
A. gambiae apoLpII/I         LFGN--GLNV FELEGRQDNL ERLVEHYFGP KGFFSGMDMQ AAYDLLAEQ- -----YQKLS GKAKERFRRG IREDIRALAR  690
M. sexta apoLpII/I           LFGQ--SYNV MELGGRQGNL DRVVEHFLGP KSFLRTEDPQ ALYDNLVKR- --FQESKKKV EDSLSRGRRS IKSEIDVFDK  722
                                          N19

P. semisulcatus apoCr        GKLYGE-S-- --RSHTHAEL FARFMGHEIT YADVAESLKG VTADTLIETF FSIFENSLEH MKDLNLN--- ----TART-A  806
F. merguiensis apoCr         GKLYGK-S-- --RSHTHAEL FARFMGHEIT YADVAGSLKG VTADTLIETF FSFFENSLEH MKDLNLN--- ----TART-A  805
L. vannamei apoCr            GKLYGE-S-- --SSHTHADI FARFMGHEIT FADVAQSLKG VTADTLIETF FSFFENSLEH MKDLNLN--- ----TART-A  779
P. monodon apoCr             GKLYGE-G-- --RSHVHADL FARFMGHEIT YADVAESLKG VTADTLIETF FSFFENSLEH MKDLNLN--- ----TART-A  806
M. japonicus apoCr           GKLYGE-G-- --RSHTHAEV FARIMGHEIT YADVAESLKG VTADTLIETF FSFFEESLEQ MKGLNLN--- ----TART-A  806
M. ensis apoCr1              NKLYGE-R-- --ASDVRAEV FARIMGQEVT YANIAETLKG VTADKIIETF FSLVDDSLEE LKGLNLN--- ----TSKELL  808
M. ensis apoCr2              NKLYERGT-- --KEKSRADV FARIFGHEVT YASIAENLKE VDTDRIIYSI FSIFDDVLPV IERLDVN--- ----TART-G  808
C. quadricarinatus apoCr     HNLYSD-E-- --SRLAKADV FARFMGQEIS FASLAGDLTD ISADRFIEAF FSYFNDIVDQ MKHLNINSAH LNINSART-A  812
M. rosenbergii apoCr         DKLYGNR--- ---HIQKADL YARINNQELA FGSWEGNMKN IKIDELINSL FDKFDNMINR AATSNID--- ----TVRT-A  804
P. hypsinotus apoCr          DKLYGDRR-- --SRMPKADF YARVNDQEMA FASLAGDLRN INVEELITRF FDSVDDMITR AANANLD--- ----SVRT-A  811
P. trituberculatus apoCr     SKLYGPHYG- ----KFQADF FARFLGQEIN YASLSDNLQD INIQHLVEAS VRYLMQMLSS LKNMDLD--- ----MVKA-A  807
C. feriatus apoCr            SKLYGPQYG- ----QFQADF FARFLGQEIN YASLSEHLQD INMHHLVEAS VRCPMQMLSS LKNVNLD--- ----KVKA-V  805
D. melanogaster apoLpII/I    KYKMYGVKNV Q---DLNLDV SLKLFGSELA FLSLGDNIP- SSLDDIINYF STSFEKAKQE LSSFEKQ--- ----FSSHHL  786
A. mellifera apoLpII/I       KNVHLRSNEV --DQDLDIDL SIKLFGVEYA YLSYQGEGSK LSPEAVIDKL LDGLEKGFDV TKNLKSD--- ----LENYLQ  829
L. migratoria apoLpII/I      QQAYKSLLPS QRDRSLDVDL SLKTFGSELA WFNYDGKHEQ KSSERVVDEI FDAIDEGLKK SKKFNYD--- ----FEPHFT  804
A. gambiae apoLpII/I         TVDLHNDALK ----DFNLDV TMKVFGSELF FLSTGENVP- TDPEQFLDKA LECFDKMIEG AKKFEHT--- ----FEHHAL  758
M. sexta apoLpII/I           NLKAESAPYN N---ELDLDI YVKLFGTDAV FLSFGDDKG- FDFNKMLDQI LGGCNSGINK AKHFQQE--- ----IRSHLL  791
                                                N20
```

```
P. semisulcatus apoCr        QLSMDYSLPT IQGTPLTLKL AATAVAGLKM EGNVNIAQIL SD-LGNSQTG IKFFPGLSVQ ATGFVGFECR FTKVGIEMQN  885
F. merguiensis apoCr         QLSMDYSLPT IQGTPLRLRL AGTAVAGLKM EGNVNIAQIL SD-LGNSQTG VKFFPGLSVH ATGFVGFDWL LARVGIEMQN  884
L. vannamei apoCr            QLSMDYSLPT IQGTPLKLNL AATAVAGLKM EGNVNIGQIL SD-LGNSHTG IKVFPGLSVQ ATGFVGFECR FTKVGIEMQN  858
P. monodon apoCr             QLSMDYSLPD IQGTPLRLKL AGTAVAGVKM EGNVNIGQIL SD-LGNSQTG IKFFPGLSVQ ATGFVGFACG FTRVGIEMQN  885
M. japonicus apoCr           QLYMDYSLPT IQGTPLKLKL AGTAVAGLKM EGDFNIAQIL SD-PGNLQTG IKLFPALSVQ ATGFVGFECR LTRVGIEMEN  885
M. ensis apoCr1              KLHLYYSLPT IQGLHSRSPW KGTAVSGLKM EGNFHIAHIL AN-PGNLDTA IKLYPGLSIH TTGFVGFDAF IAKAGIEMKN  887
M. ensis apoCr2              QIMLEHSLPT IQGTPLKLKL HGTAIVGIKL AGDLNIIEFI TS-PASAEKS IKLIPSISAN VHGFVGFDCD IAKAGIELEG  887
C. quadricarinatus apoCr     QLHLDYYFPT IQGTPFKLKM DGTAVLGRQI EGNLNLVNMI AN-WKKGESI LKLVPSLSVE VDGFVGYDCH ISKTGPEMKN  891
M. rosenbergii apoCr         QFYLDYHLPT MQGLPIKMKL EGTAVAGIKV ETQVRGL--- ---MSGTPGA VKFLPSLSTR IDAFIGYDCH IVRAGIKMKN  878
P. hypsinotus apoCr          QIYVDYHLPT MQGVPLKMKL EGTAVVGLKM ETRLS----- -----GSPNI IKFTPSLSSQ VDGFIGFDSH IARVGVQMKN  881
P. trituberculatus apoCr     QLGVDYSLPT IQGTPLKMKL ETVAVAGIKM QTNLN--GLF SG-QGSSGSL FKILPSFSVE THGFIGYDAY ISKSGLKMNT  882
C. feriatus apoCr            QLGVDYSFPT IQGTPLKITS ETMAVAGIKM ETNLN--GLF SG-QGGSESK LKILPSFSVE THGFIGYDAY ISKSGLKMNT  882
D. melanogaster apoLpII/I    FLDTDLAYPT SIGVPLELVA QGFAATKVDL AVSLDINAIL EQNWQKAKYR LKFVPSVDIN ANVQIGFNAQ VLSTGLRVVS  866
A. mellifera apoLpII/I       FLDNELVYPT NLGTALSLGL SGTSALRLKT QGKFDLKSAL KD-PKNTNFR LAVEPSVSIR LAGSMTVQAP GVESGMKIIG  908
L. migratoria apoLpII/I      FLDSELSYPT NLGFPLKLAI DGSIAARLKL NGEVDVRSIL RQ-PENAAFR LEFVPSAAVE LTGKLLVDAY VVEGGLKLDY  883
A. gambiae apoLpII/I         FLDTDLVYTT ALGLPLKLSA QGAGVARVDA ALELDVKSLV KD-YSNAKFN TKFQPSGSFE VTGTMSVDAF NVMTGMQVAV  837
M. sexta apoLpII/I           FMDAELAYPT SVGLPLRLNL IGAATARLDV ATNIDIRQIF QS-PQNAKAD IKFVPSTDFE ISGAFIIDAD AFSTGIKVIT  870
                                           N21                                          N22

P. semisulcatus apoCr        TISSATGVAI NIRTT-ENKK IEMELEIP-E KMELLNIKAE TYLVKAV--- GKKMTKISPS SMRDVRIQHN SCIGALEPVF  960
F. merguiensis apoCr         TISSATGAAI KIRTT-ENKK IEMELEVP-E KMELLNIKAE TYLVKAV--- GKKMTEISPS SMRDVRIQRN SCIGALEPVF  959
L. vannamei apoCr            TISSATGAAI NIRTT-ENKK IELELEIP-D KMELLNIKAE TYLVKAR--- GKKMTKISPS SMRDVRIERK SCIAALEPVF  933
P. monodon apoCr             TISSATGAAI KIRTT-ENKK IEMELEIP-E RMELLNIKAE TYLVKAV--- GKKITKISPS SMRDVRIQRN SCIGALEPVF  960
M. japonicus apoCr           TISSATGASI NIRTT-ENKK IQMELEIP-E KMELLNIKAE TYLVKAV--- GKKLTKITPP TVRDVRVTHA ACLNAVEPVL  960
M. ensis apoCr1              TISSATGAAI KLRTT-DNKK IEMELELP-E KMELINIKAE TYLVKAV--- GKNVRKIAPS SMRDERFAHE ACIDALEPAL  962
M. ensis apoCr2              TIASANGATV HIRKN-GANN FEAELELP-E KMEAINMKAE TYLVKAI--- EKKVTKILPP SVTDVRIKRD SCIEALEPVL  962
C. quadricarinatus apoCr     TISSSNGVSF MVRPK-NNNE LELELDIP-A KMDFIDIESK IHFIKEVK-- GKPKTTVSVS PSSEDEVTHQ SCSNSLESGI  967
M. rosenbergii apoCr         HIATNYGASI NAKYT-PGEG FEVELELP-E KMELVKAQSE TYLMKRVK-- GQQETKVHPS SVQSTRFQTQ SCVTKMEPVL  954
P. hypsinotus apoCr          HLATTSGGSI NVKAT-RSNG YELEVDLP-E KMELLNMRSE TYLVKALK-- GQQETKINPS SVRSTRVQKQ SCMRALEPML  957
P. trituberculatus apoCr     TVSSNNGVAI KVGGQ-SSQE VQIEVDLP-K KMEIIRVQSE TYLKKQTR-N -QPEIKILQP SMQDIRIRHN SCFTALESVF  960
C. feriatus apoCr            TVSSSNGVAI KVGGQ-SSQE LQIEVDIP-D KMEIIRVHSE TFLMKHHE-N TSQTTRSCQP SMQDIRIRRK SCFTALESVF  959
D. melanogaster apoLpII/I    SAHSATGSDI TVAVISDGEG FNVDLELPRE KLELINFNVD TELYVAEQ-- DKQKAIALKG NKKNKNSQPS EICFNQLELV  944
A. mellifera apoLpII/I       TLHTSTSTDV SVSLL-DGTG IDVNIGIPKK KQEVISVSSE VLFS------ -TP--KGDVA PKFGKSKEYA DCFEQFSSYL  978
L. migratoria apoLpII/I      NVHSSTGINV AVHNL-NDLG IDIKVGLPVK KQDIIDVKTD VLTTVKERGH PETSTPLHFN LKGNDYKQYR GCFDQLSPVS  962
A. gambiae apoLpII/I         SGHSSGGAAV KFALH-DATA YDLTVDALQG KQELVSVNFR EVIITRE--- RGNQLITLPA KRQDFGSKFS ECFDNLYSVI  913
M. sexta apoLpII/I           NLHSSTGVHV NAKVLENGRG IDLQIGLPVD KQELIAASSD LVFVTAE--- KGQKEKQKVI KMEKGENEYS ACFDQLSGPL  947

                                                   DUF1081 domain

P. semisulcatus apoCr        GLKVCYDMSI P--------- ---DVFRANA LPLGEPAIAK LYVEKADPSM RGYLVTAAIK NKRGNKVIKM NVEAAGASTP  1028
F. merguiensis apoCr         GLKVCYDMNI P--------- ---DVFRANA LPLGEPAIAK LYVEKADPSM RGYLVTAAIK NKRGNKVIKM NVEAAGASTP  1027
L. vannamei apoCr            GLKVCYDMNF P--------- ---DVFRANA LPLGEPAIAK LYVEKADPSM RGYLVTAAIK NKRGNKLIKM NVEAAGASTP  1001
P. monodon apoCr             GLKVCYDMNI P--------- ---DVFRASA VPLGEPAIAK LYVEKADPSM RGYLVTAAIK NKRGNKVIKM NVEHLGALTP  1028
M. japonicus apoCr           GIKVCYNINM P--------- ---DVFRANG LPLGEPAIAK LYIEKADPSM RGYLMTAAIK NKKGNKFIKL NVEAAGATTP  1028
M. ensis apoCr1              GLKVCYNVNI P--------- ---DIFRANA LPLGQPAIAK VYIEKADPSM KGYLVSATIK NKSGNKMIKV GVEAVGASTP  1030
M. ensis apoCr2              GLKMCYEMNI P--------- ---DVFRCKA LPLGEPRVAK SFVEKADPSM RGYLMLLPSR GGQVTRSSRL TLDTHGASSP  1030
C. quadricarinatus apoCr     GIKLCYKFNF F--------- ---DILRSNS FLQGTPFVAK LFLEKVDPSV KGYIVKTAIQ GDSNRKLIKM KLEVPGATTA  1035
M. rosenbergii apoCr         GLKLCYDVNM P--------- ---NIFRSQG LPLGPPAIVK VVLEKADSGM RGLRVKGREE HAGGKKVIKV ELEAPGSSSP  1022
P. hypsinotus apoCr          GLKLCYDINM P--------- ---NVFRSEG LPLGPPASAK VFIEKSEPSM KGYRMRANVE GAEGKKEIKV EVEALGSSTP  1025
P. trituberculatus apoCr     GIKMCYDVNV P--------- ---DIFRANA LPLGSPALVI LSLNKTESTI TGYKIAVNAH TDTQDKKYAA KVSAVGSSSP  1028
C. feriatus apoCr            GIKMCYDVNV P--------- ---DIFRANA STTGIPSTGY THLNKTESTI TGYKIAVRAD TETQDKKYAA KVSVAGSSSP  1027
D. melanogaster apoLpII/I    GLNICIKSST SLSEV----Q AGNGNVAERG LSVSEKFHLS RPFNFAVYLT TERKFTFKGI HTQEAFSQKW KLDYSTPGSK  1020
A. mellifera apoLpII/I       GLTVCGKISY PY-------D DSSSIDQKPL FPLNGPAEFA VKVENNDVNK YHFKIFANNN AEANKRSFEI LLDTPNSKMN  1051
L. migratoria apoLpII/I      GLTFCGNVSV P--------- WVSPTQAAAF YPLNGPSHLS VSIEADDVSE YHFRAEIK-K DESAFKSAAV LFDTPGSSAD  1032
A. gambiae apoLpII/I         GVTVCAAHKV D--------- --QEELFEVY LEVEPKFHFS GKFDNSNPQH LQLSLSFDTP GSQTKRLTNL RLEGVTAG--  980
M. sexta apoLpII/I           GLTMCYDMVL PFPIVNRNDK LDSIAKAMGK WPLSGSAKFK LFLEKNDLRG YHIKAVVKED KDAGRRSFEL LLDTEGAKTR  1027
```

DUF1081 domain

```
P. semisulcatus apoCr        RRAEMTLSYT K-EEGSHIVS AKLDSSSIAA GVWTTLTNEQ GHKAVETYVN FNYGQIA--- --ISRG---- IKLDVIAREG  1098
F. merguiensis apoCr         RRAEMTLSYT K-EEGSHIVS AKLDSSSIAA GVWTTLTNEQ GHKAMETYVN FNYGQIA--- --ISRG---- IKLEAIAREA  1097
L. vannamei apoCr            RRAEMTLSYT K-EEGSHIVS AKLDSSSIAA GVWTTLINEQ GHKAVETYVN FKYGQTA--- --ISRG---- IKLEAIAREG  1071
P. monodon apoCr             RRSEMTLSYP K-EGGTHFVS AKLDSSSIAA GVWTTLPNEQ GPKAVETYVN SNYGQIA--- --IFSC---- IKLDVIAREG  1098
M. japonicus apoCr           RRAEMTLSYT K-EEGSHIVS AKLDSSSIAA GVWATLTNEE GHKAMETYVK FDYGQIA--- --ISRG---- IKLDMIVKEE  1098
M. ensis apoCr1              READMTIAYT M-EEDSHIIS AKIDATSITA GFWATLTNTE EYKAIETFMK IKAGAMD--- --ISRG---- IKVDLSAKEA  1100
M. ensis apoCr2              RQAEMTLSYA K-EQESHIVS AKLDGTGVGA GIWVTLTNEA EYKAFETFVK YKAGRTN--- --IAQG---- VKVDLSAKRE  1100
C. quadricarinatus apoCr     RDVETVVSYT K-EEDSYKIS AELGFSDVSS KFGAHFTNKV NQKGVQAFAK YKSSDTE--- --IFHA---- IKADIMARST  1105
M. rosenbergii apoCr         KKATAIITSA N-EGEEKKIS ATLESERLGG ---------- ---------- ---------- ---------- ISIHMIRKWT  1061
P. hypsinotus apoCr          RKANGIISYS R-NQDMTKIM CTLESQTSGG ---------- ---------- ---------- ---------- IKVEIKKKWT  1064
P. trituberculatus apoCr     KESNVEVNLK R-HGESYLAE VKLMSSLTHG KIKIGLVNRP ELKTFETEVS LTTSGME--- --FLQG---- FIVETKITPI  1098
C. feriatus apoCr            KEADVEVNLK R-DRESYLAE VKLLSSVTHG KVRISLVNRP EQKSLQTDFS FMKDGVE--- --FLQA---- LRIEIKKTHT  1097
D. melanogaster apoLpII/I    VSHDTTVVYE LGNKPKTFSR LSFDNSQCHF AVEGGINNDK NELVVYGQYE QDKEIKKSKI GFSKNG---- NEYKPLIEIQ  1096
A. mellifera apoLpII/I       RRMALLLESS M-QHPNMYVK GSLDTPFKKA SAEAVLKDTA QERTLTVTVK HDQMEYYGRV GVLASG---- SKYKPIMEYK  1126
L. migratoria apoLpII/I      RKVLLLVEK- K-EKPHQGIT AHLKSGWKEI VAEGLLIDDN NEKSVSAKLV IESDEYPIKG GVKISGNPSR QVSKPILEYK  1110
A. gambiae apoLpII/I         ---ELYVKAT L-ESPIRNVD FKLGVNNNDK EVALYAVAHN GVEEYLAKIG FQKGASGGR- ---------- DEYVPIFTIR  1045
M. sexta apoLpII/I           R--SQLTGEA VYNENEVGVK LGLEAVGKVI YGHIWAHKKP NELVASVKGK LDDIEYSGKL GFSVQGNEHR AVYKPIFEYS  1105


P. semisulcatus apoCr        SVGEEFHVHV FSSGTRSFPS ESRIVEAKFI KNASGPEFNV DVICRTKN-- ------ALAE IFDLNIEVGV DFMKFSPRDL  1170
F. merguiensis apoCr         SVGEEFQVNV FSSGTRSFPS ESHIVEARFI KKTSGPEFNV DVICRTKN-- ------ALAE LFDLNIEVGA DFMKFSPKNL  1169
L. vannamei apoCr            SVGEEFQVNV FSSGTRSFPL DSHIVEAKFI KKTSGPEFNV DVICMTKN-- ------ALAD YFDLNIEVGA DFMRFSPKAL  1143
P. monodon apoCr             SVGEEFQVNV FSSGTRRFPS ESHIVEAKFI KKTSGPVVNV DVICRTKN-- ------VLAQ YFNLNIEVGA DLMEFSSEDT  1170
M. japonicus apoCr           SAGKEFEVNV FSGRSRRFTP ESHIVEAKFI KKTNGPEVNV DVICRTRN-- ------ALAQ YFDLNIEVGA DFMEFSPEGV  1170
M. ensis apoCr1              GAEEVYAMNV FSGRNKKFTA EAHIFEAQ-I KKENAPEMKV DIFCKTKN-- ------ALAE YIGLNIEVGA DLMMSHPEVL  1171
M. ensis apoCr2              GNENTYEVNV FSGHNRRFTD ETHIVETKFI KRTNGPETHV EVVCNTKN-- ------ELAE YLELKLDVAS DFAWMS----  1168
C. quadricarinatus apoCr     TEDMEYHVTM YVSRSEDFSP QSQVIEWKSS KKNNGPEISL DILAKTKN-- ------AWKS YIDLNLEGGV DLRYAS----  1173
M. rosenbergii apoCr         QSEKQVQMIA YFSESRQYNP STKGIEAKFL WNGEGEEVKV DVALQTLA-- ------AVRE WAQFNFEVSG DLRYHE----  1129
P. hypsinotus apoCr          QSEKRIELDT FASRSRQYSP DSKCIETKFM MTDDGQEIKV DTVLRTMD-- ------TLQR YIDINFEVSG DLKYSE----  1132
P. trituberculatus apoCr     QNGIKYDMDV YCSPSGSISE QSKIFTGMLK LEHMLPNMLM EIKGETKN-- ------ILSQ YFPFSFDAVV FFKYYND---  1167
C. feriatus apoCr            SYGMKYDMDV YWSPSNKISQ ESQIFTGLLK LDYMLPNMVM EISAETKN-- ------ILKQ YILFSLQAVV DFKYYDG---  1166
D. melanogaster apoLpII/I    DNNGISNSIN GYHADGKIVV KKNSNNIERY NFENFQVSNS NNAHVAVN-- ------GWSD VGTNSLTSEL RISLDHQTFL  1168
A. mellifera apoLpII/I       VPEHIEKLAS SKTGLPSGQQ YYLDGSVDVT DNQDGKKFNL EKVAFVLNGQ KLIVIDGPVT WTSNSVNVDT NIGYGDKNLA  1206
L. migratoria apoLpII/I      APAKDSGAKV KKSHKTSEGI TVDGAVVVER TSDKGKYTFQ DLSLKTPKG- -TFVINGQLD IVPRNYAFDL KLSVDKNELL  1188
A. gambiae apoLpII/I         SPNGDAQVSK FVQTTGKIVV ESLDGGKRKY NLENIEWTSP YAPKTTING- ------HVVS NGERSFDANV DVNVGDVKNN  1118
M. sexta apoLpII/I           LPDGSSPGSK KYEVKIDGQV IRECDGRVTK YTFDGVHVNL QNAEKPLEIC GSVSTVAQPR EVEFDVEVKH YASLKGSWKG  1185


P. semisulcatus apoCr        YPAR------ ---------- -YMPKTRIVL PVSVRKMEIN AAAAAWKLTS YIRAGSQSGE SREFISALKL AKG-------  1226
F. merguiensis apoCr         YPAR------ ---------- -YIPKTRIVL PVNLRKMEIN AATAAWKLIS YIRAGSQSGE SREFISALKL AKG-------  1225
L. vannamei apoCr            YSTR------ ---------- -YIPKTRIFL PVNLRKLEIN AATAAWKVTS YIREGSQSGE SREFSSAFKL AKG-------  1199
P. monodon apoCr             YRTR------ ---------- -YIPKFRILF PITLRKMEVH AETGAWKLAS YIREGSHSGE ICEHISALRL TKG-------  1226
M. japonicus apoCr           YPAR------ ---------- -YIPKVSILL PVALRKMEVH ANTVAWKLAS YIREGSQSGE SRELISAFKL SKG-------  1226
M. ensis apoCr1              YVTK------ ---------- -YIPRVPITL PVKIRKMEVT TDIGGWKLES FIREQRESPE TCEHISALKL TKG-------  1227
M. ensis apoCr2              ---------- ---------- ----FAPVPI PVKLHKAEFQ TGALGFKINS FIRKTTETNN EAMHNAAFKL EKD-------  1217
C. quadricarinatus apoCr     ---------- ---------- ----HSRVPL PRKLRKFEFH TGLGGWKVIS FVRQTSESGD KVEFSSAFKI ARR-------  1222
M. rosenbergii apoCr         ---------- ---------- ----WCRIPL PQRLRKFETN IALRNFHIVS FVRKEGES-- --QYRSALKL GQR-------  1174
P. hypsinotus apoCr          ---------- ---------- ----MTGIPL PRRLRKFEMA LASERWHVVS FVRQAGGS-- --QYNSAFKF GQK-------  1177
P. trituberculatus apoCr     ---------- ---------- -------VPI PLWLQHFELT VGINSWMMKS FFRNSGESSQ SSLILRVSPT VPR-------  1208
C. feriatus apoCr            ---------- ---------- -------VPI PVQLRQFELT VAINSWMVTS FFRNSRASSQ EVIILRVSSL LHQ-------  1212
D. melanogaster apoLpII/I    IKENLKLENG LYEAGFFIND EHSPENIYGS SIHLTIADQS YALKTNGKAA AWSIGSDGSF NFQKLADSNS ARAG------  1242
A. mellifera apoLpII/I       FKLDGQCSKD DHRLVVSAMP SSDPNIGFNL NWQLKKTDNN LENKFFFVHG PDPNSQTNRL SLTQKAVYKL SNKEFFLSVS  1286
L. migratoria apoLpII/I      LNGHLN---- ---------- -YAEPKSIDV ALEVTSPQFP DYGSGFQLIN -KRGDDYSDT KIILACGRDL KSDGSRLILE  1252
A. gambiae apoLpII/I         VVGHLDFDLK HVKLDLEKKT PSDANKNFKV NLEVAYTDNS FKNLFSFASG KDFNNPSNKY ELSQYAEFEL KPEAQGLESL  1198
M. sexta apoLpII/I           SDVVLAFNN- ---------- QLNPKINFDL KGKFENTDSM HNELDIHYGP -NRGDNNARI TFSQILKYHV ENSKNFN---  1250
```

```
P. semisulcatus apoCr         ---------- ---RKDIISV QATHTIEGT- ---------- ---------- --FPQNIIIK NVATAEVADS ----------  1260
F. merguiensis apoCr          ---------- ---RKDFISV QATHTIEGT- ---------- ---------- --FPQNIIIK NVATAEVGRS ----------  1259
L. vannamei apoCr             ---------- ---RTDVIFV QATHTIEGR- ---------- ---------- --FPQNVIIK NVATAKVGRS ----------  1233
P. monodon apoCr              ---------- ---RKAIIIV GATHRIEGA- ---------- ---------- --FPENIIIK NEATVEIGRS ----------  1260
M. japonicus apoCr            ---------- ---RNDIIYV QATHKIEGT- ---------- ---------- --LPQNIVIE NEATVEVGRS ----------  1260
M. ensis apoCr1               ---------- ---RRDIISV EATHTIEGR- ---------- ---------- --LSENIILK NEATVVLGRS ----------  1261
M. ensis apoCr2               ---------- ---SDEVISL EAAMKVKGR- ---------- ---------- --TVRNMVIE NEFSAKFGQE ----------  1251
C. quadricarinatus apoCr      ---------- ---NEDFISL EATHTTQGR- ---------- ---------- --LYTDFVCK TTAKVKMGRA ----------  1256
M. rosenbergii apoCr          ---------- ---GNEKVAI MGNHVMEGG- ---------- ---------- --SYRDMTLK SNIEAKIGNA ----------  1208
P. hypsinotus apoCr           ---------- ---GSEMVSV EATHTIEGS- ---------- ---------- --SYRDLTLQ SDMKGKIGST ----------  1211
P. trituberculatus apoCr      ---------- ---REMLIDI KADLRLQGT- ---------- ---------- --PAVNFIEQ LTVDAKIGQT ----------  1242
C. feriatus apoCr             ---------- ---REMLINL QADLPLQGT- ---------- ---------- --PAVDFIQQ LFIDAKIGQT ----------  1246
D. melanogaster apoLpII/I     ---------- --SLVENVEI QYKNKQVGG- ---------- -------IKI MSNFDVNKMD VDVEISREQK IGSIIVKYES  1292
A. mellifera apoLpII/I        NELTYPIVNL KLKYEGELTQ KSVSSDLEF- ---------- ---------- --KYKEFNFG TELSAKVGTE K---------  1334
L. migratoria apoLpII/I       HFIKG----- KYETPDTFNL ETKGEVLGTG HKIFGKFDID SKPKHLEYDL KLGFDENEVT SDLVAKRDIK S---------  1318
A. gambiae apoLpII/I          KLENKLQLPK QLIRLDFATN KNKFYLDGEY G--------- ---------- ---YDKYKIA ANVDAKYNEK T---------  1247
M. sexta apoLpII/I            ---------- -VITKNNLEI RAVPFKLVAN ---------- ---------- -ADVDPKKID IDIEGQLQDK SAGFNLDART  1298

P. semisulcatus apoCr         ------SYKA MYDIFYHPEK MGASVEVLQA AGNEKFAHLE AIYEISGEKY CTKFLAEIPG -YIQPVKVHA DIEQEAEG-R  1332
F. merguiensis apoCr          ------SYKA MYDLFYHSEK MGASLEVLQA AGNEKVAHLE AIYELSGSKY CTKFLAEIPG -YIQPVKVEA GIEQGAEG-R  1331
L. vannamei apoCr             ------SYRA MYDVFYHPEK VGASIEVLQA AGNEKVAQIE AIYEISGEKH CAKFLAAIPG -YIQPVKVEA EIEQEAEG-R  1305
P. monodon apoCr              ------SYKA IYDVIYQAEK VGASMEVVRT GDNEKVAKLD AIYQHSGAEH STNFLLKVPK -YMKPLKIQA KIGEETEG-H  1332
M. japonicus apoCr            ------SYRA MYDIFYHPEK MGASVEVFRT AGNEKVAEME AIYENTGEKY YTKFLVEAPG -YIRPVRIEA TAEEETGG-R  1332
M. ensis apoCr1               ------SYKA KYDVFYHSEK VGAAVEVVRA GDNEKIVDVE AIYKRSGETH IGKFAVEIPE -YIRPVKAEV RIAEEED--R  1332
M. ensis apoCr2               ------SYVA SYNVFLKPTK IGTTFEVVKP REGKKIVELE VMYEGSGDTH NSRVLIDAPE -YMRAVKFEG KMMEEERG-K  1323
C. quadricarinatus apoCr      ------EYKA ASSIYYEHGK KGVSLQVIRS EDNVKVADLE ATRVCTIQSC RIYAAMDMPG -YMKVLKFEC KEEEQGPG-R  1328
M. rosenbergii apoCr          ------RYKN NFILYNQESK IGAEWQVASQ -EGGKVIELE MILIRSGETS QVKFLLDIPE -RMKKVMIEA SAAGQGSS-Q  1279
P. hypsinotus apoCr           ------QYKT QFAIYCNDAK MGTKMQVTRE GDSAKMMEVE MLLTPSGENY RLNFLLDIPA -YIKAMKVDA SATHQGSS-Q  1283
P. trituberculatus apoCr      ------EYKM QRRIAYQERR RAISLQLTRP --TDNVKLLE IAAEKDSQEI MFLFWVQMPE -YMAPIKVAA SFVQQGQ--D  1311
C. feriatus apoCr             ------EYKL QQRILYQETR GAISLQLTRE --SDNVKLLQ IHVEKDAHEI KFLFWVQMLE -YMAPIKVVA SAVQQGQ--D  1315
D. melanogaster apoLpII/I     NQRHAQDYSL EASAKINKHS IDVISKCDFN GNVYVVDNSL VTSWGTLLSA KGEIGQRYSA -QDININIQG NVQISGK--D  1369
A. mellifera apoLpII/I        ----PGDYEV EFEAQLMENG IELKAKRKIL DGQKSQFSNS LELKPGGKYT ADAVVLYSIA -SKSNINFEL DGDANLNGKK  1409
L. migratoria apoLpII/I       ----PDDYEL KFSAKILDNS IRIESSREVK PKDDSAFLNT LVVLSGKKYE FQVDVKLAAE -DEYHTSLKA ESNLKIEG-K  1392
A. gambiae apoLpII/I          ----PGDYDV QLGGSLNKHF FKFFSKRIVE ANKSRFSNKL TASTGTKFEL NGAVTNRFTS -QDGELNLEG SLIAVEKASP  1322
M. sexta apoLpII/I            HIKKEGDYSI KVKANLNNAN LEAFSRRDIV NAEKSNVENY IDMKGVGRYE LSGFVLHKTK PNDVNVGFIG HLKINGGGKN  1378

P. semisulcatus apoCr         YTLESVITYG QLTVLEVSGP IMARFSSKIA KLQANMKLRA ---------M ASAPYIIG-- -ANVA--FGS KKQMIALEIK  1398
F. merguiensis apoCr          YTLESAITYG QRTVLEASGP IMARFSSKIA KLQANIKVRA ---------M ASEPYIIG-- -ANVA--FGS KKQMIAMEIK  1397
L. vannamei apoCr             YALESAIKYG PRTVLGVSGP VLARFTSKAN KLQANIKLRA ---------M ASEPYIIG-- -ANVV--FGN KKQMIAMEIK  1371
P. monodon apoCr              YMLEAAIKYG QHMILEANGP VMARLASKIA KLQANIKLSA ---------L ASEPYIIG-- -ANVV--LGN KKQVIALEIK  1398
M. japonicus apoCr            YALESAIKYG ERTVFEVTGP VMARFTSKTA KLQANIKLSA ---------M ASEPYIIG-- -ANFV--FGN KKQMIAMEIK  1398
M. ensis apoCr1               YILEAAIRHG QRTLLEATGP VVARFSSKIA KVQANIKLVA ---------F NTEPYIFA-- -TNLV--FGN KKQVIALEIK  1398
M. ensis apoCr2               YAIEVAFKNG ERTLLQVDGP VTAIMTPRKH KIETDVKISL ---------L DMEPHIIS-- -TTIL--TSE RKQILAFEMK  1389
C. quadricarinatus apoCr      YVVEAAIQHG DPVIFQAEGP VTAKISSDSA KLQTDIRFIT ---------I SRQPFKLS-- -SNFV--FAK NKQVLSLELI  1394
M. rosenbergii apoCr          YQVRAMAKHG ESPIFHVEGP VTAMLSPKNT HLKAELRAVL ---------L RSQPHTVT-- -ASLV--MMH GKQAFVVELK  1345
P. hypsinotus apoCr           YQIGVGVKHG ESVILHVEGP VTAKLSSRLT QLQTQLRISC ---------M NGKPSTVS-- -TSVV--LAP GKQSVAFELV  1349
P. trituberculatus apoCr      YDAEAIIMHG QQTLVQLHGP VTYINSPTLT KVGANIKIN- ---------- --NFYQFV-- -SSFE--YEE GKQIVLLEIK  1373
C. feriatus apoCr             YNAEAIIMYG QQTLVQLHGP VTYINSPTLA KVGANIKIN- ---------- --NVYQFI-- -YSFE--FEE NKQNLLLEIK  1377
D. melanogaster apoLpII/I     KVTQWILKVI GTPDKTNSDF RISRDTSELI KLTSESQHPQ DKISFAKLNL IVKNQLTAKG EFRVAK-NGK GDFTASIDTL  1448
A. mellifera apoLpII/I        VKINTALESN PQSFNALLNA KVNDVTYVEF DLKNKRSPNP S----GVLVL NLKNYLTANG QYSYQ--NGK GNAKLDVDVP  1483
L. migratoria apoLpII/I       TSVRLITDFT TDAQTVNGHV KVSNEGEDFF ELIYKLNRGS G-NPSGNAKL FVKNYLDGAA TFKYN--NGV GSGTLQIDVL  1469
A. gambiae apoLpII/I          YKLSLTVQFS AANVLSNAKV LVDKEEFATY DFKLERGQDP N----GKFTF VVKDFFNGNG ELKSA--KGQ GELFALVTFV  1396
M. sexta apoLpII/I            EDFKINIGHI ETPAVFSSHA TISGSRGDII DYLLKIMRTA N--PNGNFKL VIKDSIAANG QYKVTDADGK GNGLIIIDFK  1456
```

```
P. semisulcatus apoCr      ARS--ESIIG LEWKMVRESS E--------- -KTTVGVVFV LPALIENKLD AEITEGLIHV SFNNLVLPKT SSRR--RVKG  1464
F. merguiensis apoCr       GRS--EAVIG LEWKMVRESS E--------- -KTTVGIVFV LPALIENKID AEITDGLIHV SFNNLVLPKT SSRR--RVKG  1463
L. vannamei apoCr          ERS--EPLIG LEWRMVRESS E--------- -KTTIGVVFV LPALIEQKVD AEITDELVHV SFNNLVLPKT SSRR--RVKG  1437
P. monodon apoCr           ERE--EPLFG LEWKMVQESS E--------- -KTTVGIAFI LPALIENEMD VIITGEFVHV NFNNVVLPKT SSRR--RVKG  1464
M. japonicus apoCr         ERE--EPVFG VEWKMVQESA E--------- -KTTLSIAFV LPALIENKVD AVITEDLVHV SFNNLVLPKT SYRR--RVKG  1464
M. ensis apoCr1            ERQ--EPVFG AEWKMVRENS E--------- -KTSFGVVLL LPALMENKID AVITEELIHV SFNNLLLPKR PSER--RVKG  1464
M. ensis apoCr2            SRQ--ESLFA FKWTLDAEEG PR-------- PKVFSGAKKP VPALVEFQLD ANVMQENVHV SFNTVFLPKS ASPR--RMKA  1457
C. quadricarinatus apoCr   KQR--EPVFV AEWNIKSGSS Q--------- -GTTIGIKFQ LPALVDKKLD AIISNKLIHV SFDTQFLPKS SAPR--RIKA  1460
M. rosenbergii apoCr       DRE--ERLIN VQWNMATRDG Q--------- -ETNLDFKIL IPSMIEKTVN VIVSEKILHL TFNQLIGPKS SSPL--RRKG  1411
P. hypsinotus apoCr        NHP--DRLMA VEWNMVTQNE Q--------- -ETRMDFKLI LPSMIEKSIN VIISEKVLHW SVNMLVMPKS PSPL--RMKG  1415
P. trituberculatus apoCr   KEE--EVLLA LKCNLKTPSP Q--------- -GSSLEAELN VPILFDGTTE ISITENIIHI TTNTLLLPNS PSPR--RIKA  1439
C. feriatus apoCr          KEA--QVLLS FETYLKTPSS Q--------- -GPSLQARIT YQFLLDGSME VFVSENVIHI STNTLWLPHS PSPR--RVKA  1443
---------------------------------------------------------------------------------------------------------------------
D. melanogaster apoLpII/I  KTEPKHKLEI ESKFHIQSPK YDIDAS---- LTLDGKRKVH LKSENTIEKL KFSTKNIGEA NDKIIAFEAN GSLKG-ELRG  1523
A. mellifera apoLpII/I     KID--RKIQA TGSLTVSGSK HVGELVLLYD AAKDPNKRVK LSTTSDITKT SVDTKNILEV VDKRLELNGK GSMQGTLNNG  1561
L. migratoria apoLpII/I    KLH--RKIKA TGDLTLSGSQ RSAAIDLYWD ADRDQSKQLL FKTENDVKEK SIDSKNTLKI LDKLTTLNFK GSLSGAIDDG  1547
A. gambiae apoLpII/I       KQD--RKVKL DSKFKVNAPV YDVAADFYYD FEKDNSKKVR FETKNKVTQT SFDSKNKVEV FSEKYELNVQ SQGNPKPVDG  1474
M. sexta apoLpII/I         KIN--RKIKG DVRFTAKEPV FNADIDLFLN FEKDNSDKVH FSTYNKKTDK VMDTKNKLEY AGKRTEVNIH QDGILAVTGK  1534

P. semisulcatus apoCr      FADVN----- ---------- ---------- ---------- ---------- ---------- ---------- ---------I  1470
F. merguiensis apoCr       FADVH----- ---------- ---------- ---------- ---------- ---------- ---------- ---------I  1469
L. vannamei apoCr          FADVN----- ---------- ---------- ---------- ---------- ---------- ---------- ---------I  1443
P. monodon apoCr           FAEVN----- ---------- ---------- ---------- ---------- ---------- ---------- ---------I  1470
M. japonicus apoCr         FADVN----- ---------- ---------- ---------- ---------- ---------- ---------- ---------I  1470
M. ensis apoCr1            FVDVN----- ---------- ---------- ---------- ---------- ---------- ---------- ---------L  1470
M. ensis apoCr2            FADID----- ---------- ---------- ---------- ---------- ---------- ---------- ---------A  1463
C. quadricarinatus apoCr   FTDID----- ---------- ---------- ---------- ---------- ---------- ---------- ---------F  1466
M. rosenbergii apoCr       FLDVD----- ---------- ---------- ---------- ---------- ---------- ---------- ---------F  1417
P. hypsinotus apoCr        FVDVD----- ---------- ---------- ---------- ---------- ---------- ---------- ---------F  1421
P. trituberculatus apoCr   FLDIN----- ---------- ---------- ---------- ---------- ---------- ---------- ---------W  1445
C. feriatus apoCr          FLDIN----- ---------- ---------- ---------- ---------- ---------- ---------- ---------W  1449
---------------------------------------------------------------------------------------------------------------------
D. melanogaster apoLpII/I  NGEIQGTFIF NAPDGRVIDG SINRKISTNA KSGLSQGNID AQLSDTPFGS NKKRSISLIG KLDRLNTKTK EFSANSNLVY  1603
A. mellifera apoLpII/I     QLEVE----- ---------- ---------- ---------- ---------- ---------- ---------- ---------M  1567
L. migratoria apoLpII/I    EVEGQGELIL PAGTYLGVKF GRALHLTQAD TK-----VGL HLQAEGRESA SSTQPVWKSD FLLESALTRD SFVGEAKLLF  1622
A. gambiae apoLpII/I       KFNVKVSLLL PTGRQFGGEF QRDASTKDEK RS-----GKM AASVYDKQPG GKKRSVEWAG ELKDMDVKTK FFDAVHNVKY  1549
M. sexta apoLpII/I         AHTVAELVLP TERCLSLKID HDGAFKDGLY NG-----HMD MTISDAPKRG SGASTISYKG KVSNSNLDQE IIDYEGQINF  1609

P. semisulcatus apoCr      AEK-KTNVEF SWGADNAPEK K--------- ---------- ---------- ---------- ---------- ----------  1490
F. merguiensis apoCr       AEK-KANVEF SWDADNAPEK K--------- ---------- ---------- ---------- ---------- ----------  1489
L. vannamei apoCr          AEK-RANVEF SWDADNAPEK K--------- ---------- ---------- ---------- ---------- ----------  1463
P. monodon apoCr           AEFPAAIVEF SWDADNAPEK K--------- ---------- ---------- ---------- ---------- ----------  1491
M. japonicus apoCr         GEK-RANVEF SWDADKSPEK K--------- ---------- ---------- ---------- ---------- ----------  1490
M. ensis apoCr1            TEK-NTNVEF AWDADRNPQK K--------- ---------- ---------- ---------- ---------- ----------  1490
M. ensis apoCr2            VFK-KLNAEG AWDADRNPDK K--------- ---------- ---------- ---------- ---------- ----------  1483
C. quadricarinatus apoCr   ENK-KWMADF AWDADRDQSK K--------- ---------- ---------- ---------- ---------- ----------  1486
M. rosenbergii apoCr       EGK-RANAEF AWDADRNPNK K--------- ---------- ---------- ---------- ---------- ----------  1437
P. hypsinotus apoCr        ESN-KAQVEF AWDADRNPNK K--------- ---------- ---------- ---------- ---------- ----------  1441
P. trituberculatus apoCr   TQK-QGQMMV LWNADKEASQ K--------- ---------- ---------- ---------- ---------- ----------  1465
C. feriatus apoCr          SQK-QAQMMV LWNADKDSSQ K--------- ---------- ---------- ---------- ---------- ----------  1469
---------------------------------------------------------------------------------------------------------------------
D. melanogaster apoLpII/I  TAFNGEKSEI SYQIKQQPNG DAKNIDFSLK AYGNPLPQPF EIAFA-LGDY SAQHAVVSIT SKYGEIFSVS ANG-------  1675
A. mellifera apoLpII/I     DVTLPNGRHL VYKGKRNSVK KDTKYDIQVA SKLTDYE--- ---------- ---------- ---------- ----------  1604
L. migratoria apoLpII/I    ETKGKDDLKL FLSGKSLPQG EKKANFWAFS GQGSLIGGRT SVIKL-NSEI DETFIAYNLN SECNQGYRAN IVGKINRG--  1699
A. gambiae apoLpII/I       SDLEGKDVVL DVTLKHAPAG SYKSAAGSLK VSGSLLPQVT ELSVV-VDEY CEHHAKYHVN GKYGADFTAA LVGGYHTGGH  1628
M. sexta apoLpII/I         KLKDGKNLQS TFSLKNNPDG DKFKYEFKSD VNGNLIPKPA NLVATGTYSN SENEIDETYR LKGSYGSDIG FELAGVGTIK  1689
```

```
P. semisulcatus apoCr        ---------- ---------- ---------- ---------- ------LAVD ASLISSP--- ---------- ----------  1501
F. merguiensis apoCr         ---------- ---------- ---------- ---------- ------LVLD ASLISSS--- ---------- ----------  1500
L. vannamei apoCr            ---------- ---------- ---------- ---------- ------LVLD ASLISSP--- ---------- ----------  1474
P. monodon apoCr             ---------- ---------- ---------- ---------- ------LVLD ASLISSP--- ---------- ----------  1502
M. japonicus apoCr           ---------- ---------- ---------- ---------- ------LVVD ASLISSP--- ---------- ----------  1501
M. ensis apoCr1              ---------- ---------- ---------- ---------- ------LAVD AALISSP--- ---------- ----------  1501
M. ensis apoCr2              ---------- ---------- ---------- ---------- ------LIVD ATVIGSS--- ---------- ----------  1494
C. quadricarinatus apoCr     ---------- ---------- ---------- ---------- ------IILD TNVISNP--- ---------- ----------  1497
M. rosenbergii apoCr         ---------- ---------- ---------- ---------- ------IKAE VKMVNPL--- ---------- ----------  1448
P. hypsinotus apoCr          ---------- ---------- ---------- ---------- ------VKAE VAIVSGS--- ---------- ----------  1452
P. trituberculatus apoCr     ---------- ---------- ---------- ---------- ------IALD VIIVPES--- ---------- ----------  1476
C. feriatus apoCr            ---------- ---------- ---------- ---------- ------IALD VIIVPES--- ---------- ----------  1480
D. melanogaster apoLpII/I    ---------- ---------N YNNNQALEYG LQANIEIPKS TLKSLEINSH GKVLKSLIGN ENAAYNVEFF LDSKTSLGQY  1736
A. mellifera apoLpII/I       ---------- ---------- ---------Q KGGPSRSLSY NGNVQDLDVN AITFQGN--- ---------- ----------  1632
L. migratoria apoLpII/I      ----YSPVAV KQIENTLELL LPFDKLKQLK HTIIGTFSSQ PESTPEFTVS NVIIWNNENT LKLTGEAAGD EKEGRTKWDL  1775
A. gambiae apoLpII/I         GKPATHDLKV DVGAPSYKVG VSSSGKYLQP ESDDGVYELD YSGSVDFNGK SASVSTQAKG NYNRGNGKLN LNLPNVDPIA  1708
M. sexta apoLpII/I           FLDAGDKKYL DDYTLTVRLP FEKAHDIKWV STVLFLQPQG QEMTEYTLVE SVQINADVYK IDANGKVGPK NGYGAVKVLV  1769

P. semisulcatus apoCr        ---------- ---------- ---------- ---ANPGHAE IHRDIVIAGE PYHAKLVLTA ANLIEHM--- ----------  1535
F. merguiensis apoCr         ---------- ---------- ---------- ---ANPGHAE IHGNIVIAGE PFHAKLVLTA ANLVEHM--- ----------  1534
L. vannamei apoCr            ---------- ---------- ---------- ---ANPGHAE IHGNVVIAGE PYHAKLVLTA TNLVEHM--- ----------  1508
P. monodon apoCr             ---------- ---------- ---------- ---ANPGPAE IHGNIVIAGE PYHAKLVLTA ANLVEHM--- ----------  1536
M. japonicus apoCr           ---------- ---------- ---------- ---SNPGHAE IHGNIVIAGE PYHMKLILTA TNLLEYM--- ----------  1535
M. ensis apoCr1              ---------- ---------- ---------- ---ETPGHAE IHGNIVFAGE QYHMKLVLTA TNLMRTL--- ----------  1535
M. ensis apoCr2              ---------- ---------- ---------- ---ADLGHAS VHGNIIFAGE QYHTKLNLNA EDILES---- ----------  1527
C. quadricarinatus apoCr     ---------- ---------- ---------- ---SNPGRVS IHGNVKCMNE MYHVKLDIEA ENLRQYR--- ----------  1531
M. rosenbergii apoCr         ---------- ---------- ---------- ---STLRDCV IQGNWAYLER QHQFKAELKL HDIRTWF--- ----------  1482
P. hypsinotus apoCr          ---------- ---------- ---------- ---STLQNTV IHGGWTYLDN TCQFKAELRL ADPRTWF--- ----------  1486
P. trituberculatus apoCr     ---------- ---------- ---------- ---GRPAEAT LHTKLMLLDQ AYHSNMKVVV PFLLQRY--- ----------  1510
C. feriatus apoCr            ---------- ---------- ---------- ---GRPAEAT LHVKLMLLNR AYHSNVKVVV PFLLQRH--- ----------  1514
D. melanogaster apoLpII/I    ARVNTVWNG- -TANDGSYDF EAQTNNMESP LKFNGKYHRK QTGNIKDGDL TGKQTYVLNA QYGAQYVKMD ASLGYGAEKV  1814
A. mellifera apoLpII/I       ---------- ---------- ---------- ---GQLKFVN KNGNDVQISV IGKNLNLPDN KKQRERF--- ----------  1666
L. migratoria apoLpII/I      ILPKEEPRTL ETTWSNAGDN KKAGS---LS FKWGGNKEAK VSTDIEFTSD NQPQILHLKA TSPTEKFGIF DLA-------  1845
A. gambiae apoLpII/I         AEGSYTYDAK EEGPFQTNGA LKVSYGAGKN FEFTGTAKAP SMDDIQVHAT LKSEFENVRS VDLTFKHAK- ----------  1777
M. sexta apoLpII/I           PHVEPFVLDY NYKSSHEGEK NNNYVELKTK YGKGKSASMV VDSSYAPHYS TLKVKANTPN NDKFKKLDVT VHS-------  1842

P. semisulcatus apoCr        ---------- --EGENGFKL ILTTPTQ--- -------KTV VLGASCDVQL AGATTKVIST IEYKNTKDRK YR--------  1585
F. merguiensis apoCr         ---------- --EGENGFKL ILTTPSQ--- -------KTI VLGASCDVQV AGATTKVIST VEYKNMKDRK YK--------  1584
L. vannamei apoCr            ---------- --EGENGFKL ILTTPSQ--- -------KTV VVGASCDVQL AGTTTKVLST VEYKNVRDRK YK--------  1558
P. monodon apoCr             ---------- --EGENGFKL ILTTPAQ--- -------KTI VLG------- --ATTKVIST VEYKNMKDRK YK--------  1577
M. japonicus apoCr           ---------- --EGENGFKL LLTTPSQ--- -------KTI VLGASCDVQL EGTTTKVVSV IEYKNMENKE YK--------  1585
M. ensis apoCr1              ---------- --KGENGFKL FLTTPAQ--- -------KTI AVEATTRVEL EEAIAKIMTA LKYKNSEDEE YK--------  1585
M. ensis apoCr2              ---------- ------GFDI EVTMPSQ--- -------RTI ALEAKYKIEN EGPETKIVTE GRYKNKEEEE HK--------  1573
C. quadricarinatus apoCr     ---------- --YGENGFNL ELTSP-Q--- -------RSL EWKLNTNVE- -SRSAKVDMN LQCKFQDDRE YR--------  1578
M. rosenbergii apoCr         ---------- --VGRNSLML EVTNPSQ--- -------QMY KMNAILMVEK EGSGPKVGAD VTFRTPENRE YK--------  1532
P. hypsinotus apoCr          ---------- --NGRNSMKL DFTTSSH--- -------RTY KMETVITIEQ ESSAPKVEAM ITFKTPENRV YR--------  1536
P. trituberculatus apoCr     ---------- --GERNSIHM EVQTPEQ--- -------KKW MLEVGVQKQ- --GSNTANVD LTFKSVHDNN YH--------  1557
C. feriatus apoCr            ---------- --GERNSIQM EVETPEQ--- -------KKW MLEVGVQQQ- --ESNAAKVD LTFKSVNNNN YR--------  1561
D. melanogaster apoLpII/I    DIAYVIDSSF DSVKDIKVNI RTFKPLDDST YVVTALFKQT DKSYGLDTTF YHSAHKKGVD IRLDLLKEKP IIIS---SIA  1891
A. mellifera apoLpII/I       ---------- --KDENKLII NSQIDADGAK HSLVSEIQYL DSNNLFYVMT VCPTGKTEIL SKFQKLNDKE YKGEWKVDTP  1734
L. migratoria apoLpII/I      -----LSLKK NADPADKIDF ELTVTADQKK TDVKGSLGLA PGVPIIDVVA VQPSGTSKVF VDFLRKSDSE LHGAIELQWV  1920
A. gambiae apoLpII/I         ---------- --SSDSAYNT KLQLTADDKK FSVENAVVVS ETNPSVDFTL GYPGKTVKVS GSYKALGHSA FKADAKVQN-  1844
M. sexta apoLpII/I           ---------- KNPSPDAYSN SVVVDADGRV YKIDSSIVLS KAHPVLDIQY HSPSSDKIRR LYLQGSSLSS TQGKLEVKVD  1912
```

```
P. semisulcatus apoCr        --YASVIGLE KLGGPHNYAV EAKVTYKQPG TPEIRLETAA KHH------- -----WTPE- EHVVAFKVEA DAPVLKTPVA  1650
F. merguiensis apoCr         --YASVIALE KLGGPHNYAV EAKVTYKQPG TREIMLETAA KHH------- -----WTPE- EHVVAVKVEA DAPILKTPVT  1649
L. vannamei apoCr            --YTSVIALE RLGGPLNYAV EAKVTYKQPG TAEIKVETTA KHH------- -----WTPE- EHVVAFKVAA EAPVLKTPAM  1623
P. monodon apoCr             --YASVIALE KLGGPHNYAV EAKVTYKQPG TAEIKLETAA KHH------- -----WTPE- EHVVAFRVEA DAPVLKTPAT  1642
M. japonicus apoCr           --YTSVIALE KLGGPYNYVV KAKVIYKQPE TQEIMLETEV KHQ------- -----WTPE- EHLVAFKVGA KAPVLKMPLM  1650
M. ensis apoCr1              --LTGIMALE KLRGPLGYAV KLKIICRQPE TQEIMIHALV KHM------- -----WTPS- EHLVQFKVEA EAPVLRKPVM  1650
M. ensis apoCr2              --FEAYVAAE KLEGTFGYGL ETKINYEGPE GKEMKLETAL KHE------- -----DTSE- ACEVLFKVNA EGLFLRKPLK  1638
C. quadricarinatus apoCr     --LTSVVDIE KLGSPYSYKL ESEMSFTSPG GQETTVHAEA KHQ------- -----VTSE- EREIYYKASV RTPALRKPLV  1643
M. rosenbergii apoCr         --WDSESALE WREGLQNLRL TTKADLVGPG GRQSRVNLEA MHR------- -----RTPS- QREAELQIEV TCPSLQQPIK  1597
P. hypsinotus apoCr          --LNSESSVE WLGGPYNMKV TTTADMTCPE GRQTKVVIEA KHH------- -----RTSS- QREADVQVHI TNPSLHQPMQ  1601
P. trituberculatus apoCr     --LTSSVQWQ LLDGPLCFEV QTRITFIAPQ NKQSQFVLHV KHH------- -----MSPE- QHIIYLMMEA STPSMQPPLK  1622
C. feriatus apoCr            --LTSDLQWQ RLDGPLCFDA QTRITLISPQ NKRSLFSMNA KHH------- -----YSPQ- QHIIHFTMEA STPSTQPPLK  1626
D. melanogaster apoLpII/I    ELLGDRKGKV LFEILNLADL DIKINSEASY VSIDEFYIIV NWSSKKLKLD GYELEARAQS KNIKIQLKNE NGIIFSGTAT  1971
A. mellifera apoLpII/I       KGFAKADAHV NLESVDNFVI KANFDSDKAK HRNIHAEIAN QPT------- -----AKNG- KSISITVTSD GKNIVTGSTS  1801
L. migratoria apoLpII/I      AFGGGHLTAN GDIKLDIDDF YLKLDVDSPK FNFNKWHLEA GQR------- -----AAKGS KRIVFTAKSA EKVLFSGSTN  1988
A. gambiae apoLpII/I         LANFDMEANV EANFDSYQTF YVKLYGDAPM LNTNKFSVEV NAK------- -----PGSNG KGVNFRASEG GKDILSGFAD  1912
M. sexta apoLpII/I           NINDICLDAV SEANVQKDNV AFKVVANAKE LGWKNYGIDI SSK------- -----DSGSG KRLEFHATND NKNVLSGSTS  1980

P. semisulcatus apoCr        IAFSIHNTPN AFVGFCKIER NAPSTVFEWH VHVT------ --PEGGIEAV EAGVD-MKAI IEVLKIVHAV VPLEEESYQT  1721
F. merguiensis apoCr         IEFSIHNAPN AFVGFCKIER NAPATVFEWN VQIT------ --PEGGIEAV EAGVD-MKAI IEVLKIVHAA ATLQEESYET  1720
L. vannamei apoCr            IAFSIHNAPN AFVGVCKIER TAPFTAFEWN VQVT------ --PEGGIEAV EAGVD-MKAI IEVLKIVRAI ATLEEESYET  1694
P. monodon apoCr             IAFSIHNAPN TFVGFCKIER NAPFTVFEWN VQVIT----- --PEGGIEAV EAGLD-MKAI IEVLKIVHAV ATLEEESYET  1714
M. japonicus apoCr           IAFSIHNTRG SFVGFCKIER NTPSNVFEWK IQMT------ --PEGGIEVV EAGLD-IKAI NEVLKIVHAV VTFEEEGYQA  1721
M. ensis apoCr1              VAFTLRNAEG SFTGICKMER EAPATVFGWD LKVT------ --PEGGIEAV EASLD-TKAI VEILKIVRAV VTFEETALRS  1721
M. ensis apoCr2              VEFSMENEEG SFEGICKMER DHPVTIFDWD VKIE------ --PEGEIKGI EAGLD-LKAL VNLLKVIRSV AVFQKEGRIE  1709
C. quadricarinatus apoCr     LEMSSVSQEL SYSLKALTER DSPATMINWE MKLY------ --PEGGVEKF VSSVD-MNAL RDFLKSALEI VAIEGEEYSS  1714
M. rosenbergii apoCr         TKLQLNNQQG EYTTKWVIEI GSPVNGAMYK LKLS------ --PEHGVQGF EVELN-LEEV LKLLNAIDHL ISSSSSSMVD  1668
P. hypsinotus apoCr          TKLALVNQQG QYSCKWMIEM GSPVDGASYE LRMT------ --PEGGVESF KVELK-LKAV SELMKSIESL VSMPSRGTHE  1672
P. trituberculatus apoCr     IIFSLENKDY SYVTKLQSEV SGPETLFLWQ LETY------ --PEGGVKHL KNAID-LTAI HNVLKSVSTI VGLRGSSPLV  1693
C. feriatus apoCr            ISFSLENKDY SYVTKLQSEV SSPETLFTWQ LEIY------ --PEGGIKHL ENAID-LTAI RNVLKTLSSM VGLRGSSPLV  1697
D. melanogaster apoLpII/I    YALKKELNKT IIDGQGKVQY QGKALSGNFK LTRQHFDFGT DREVGFSYTF MGNLG-SKNG LGTLKITNKE FNTKFSVCEE  2050
A. mellifera apoLpII/I       YKHRDEDGKI VVEGSGNLKV GDNTRSSSFK YTRQRLTHEK DGEAGVAIVL NANLG-PSAI VGELKLSNKE LHLFNSYCEQ  1880
L. migratoria apoLpII/I      FHSKAENNKI SYSGNGQVRI GDKAHAFNFR SSRQNLIQDA NKEIGVEYNL DFKIAGHGSL HNILKVTNKE LHALGKQCSE  2068
A. gambiae apoLpII/I         YSVKEQGKAM VIEGQGNVKL YDKQQTATFK LIREKLS--- --ESGMSATL TASVG-KFTV LHESRVQPND FRVKTSVCDE  1986
M. sexta apoLpII/I           FISKQEGQKT IIEGSGSVKV KEEQKSANFK YIRTVFTD-- SNEKGVETFF NVALG-ERSY VAESRVTNYE YKNSYVYCEE  2057

P. semisulcatus apoCr        Y---G--PQT SQYQYRFTRP S--PTSYTMQ MRTPTRTMEG -------RAK LSPR--ES-G IKFYPNKGKT ESKYEIGYKA  1784
F. merguiensis apoCr         Y---G--PHT SQYQYRFTRP S--PTSYTMQ MRTPTRTMEG -------RAK LSPR--ES-G IKFYPNKGKT ESKYEIGYKA  1783
L. vannamei apoCr            Y---G--PHT AQYQYRFTRP S--PTSYTMQ MRTPTRTMEG -------RAK LSPR--ES-G IKFYPNKGKT ESKYEIGYKV  1757
P. monodon apoCr             Y---G--PQT SQYQYRFTRP S--PTTYTMQ MRTPTRTMEG -------RAK LSPR--ES-G IKFYSNKGKT ESKYEIGYKA  1777
M. japonicus apoCr           Y---G--QET AKYQYRFTRP S--PTTYIMQ MRTPTRTIEG -------RAK LSPR--ES-G IKFYPNKGKA EAKYEVGYKA  1784
M. ensis apoCr1              Y---G--AAS KHYQMQFTRP NSFHHNTVLV KDAIFAPMEG -------EAK FVPQ--ESLA SSPHPNKGKF DTKYEIGYKM  1787
M. ensis apoCr2              R------YEP STYRYQYSKP T--PTSYTML LKTPSRTMEG -------EAH LSGP--KS-G IKFYPNKANS HSSYEVGYTM  1771
C. quadricarinatus apoCr     G---SGKYGK GKYGFHYHKP T--PSSYSMK IESPSRTLEG -------EAE YSPS--RS-S FKFYPDSDKS EAKYEITGES  1779
M. rosenbergii apoCr         S---P--RRL LFIVPTTNIM T--IILIPCC LMSPSRTMEG GC----GCNT CTGPSCADCH CSFIPHKGFS DSKYEIAIRK  1737
P. hypsinotus apoCr          -------RSE ATYLFQYHKQ T--PTSHSIL IRSPSRTMEG -------AVK YSP---NEYL VKFHPHKGVT DSKYELYAKH  1733
P. trituberculatus apoCr     T---SAYRKR NAYGYRYTNS S--TGIHSLI IEQPSRTVEA -------EAT YSPS---KVG IKFYPNRTES EAKYEVSGEY  1756
C. feriatus apoCr            T---SAYQKR NAYGYRYTRS S--SGTHSFI IEQPSRTVEA -------HAT YSPS---KMG IEFYRNRAES EAKYEMSGEY  1762
D. melanogaster apoLpII/I    KRQCTNLIVQ SIVSIDEQKL DAVEHTTLII VDLRDFGYPY EFELK-SQNT RQGLKYQYHL DSFIITGNNF KYQFTANVQP  2129
A. mellifera apoLpII/I       NK--DCAQFK LQSILNVEKK TLLKHQVTVE VDLKKFNVPV EFGLKTNTEF KNPIFDHTTN LYLHSSKDKT EYTYQAYIHP  1958
L. migratoria apoLpII/I      GK-PNCAVVE IKSKVSAADA KETTHDLVFL VDLKSVGVDT GVAFTAETVR RGFWLIDEQA SLTLSHNGET TYKYKGYLKE  2147
A. gambiae apoLpII/I         KK--KCTKLE LLSKLERAAG A-FKHEALVS VEVQQMGYEH EFG-LSAKTS ANGLKFDHTT DVQLKEKNQP KYQYLFYVHP  2062
M. sexta apoLpII/I           KK--QCAHAE IQSKIDMSTP GMIVNVINAG LDLRKLGVAP ELG-LQMRDE VSDRRPPRFT LDLHINKEDR KYHLHAYNTP  2134
```

```
P. semisulcatus apoCr          NHEG------ ---------- ---------- RWGGHASRLE FRMNHPV--- ---------- ---LPKPIMA AVQYTVAEKT  1822
F. merguiensis apoCr           NHEG------ ---------- ---------- RWGGHASKLE VRMNHPV--- ---------- ---LPKPIMA AVQYTVAEET  1821
L. vannamei apoCr              NHEG------ ---------- ---------- RWGQRASKLE VRMNHPV--- ---------- ---LPKPIMA AAQYTVAEGT  1795
P. monodon apoCr               NHEG------ ---------- ---------- RWGRHASKLE VRMNHPV--- ---------- ---LPRPIMA AAQYTVAEET  1815
M. japonicus apoCr             NHQG------ ---------- ---------- SWGQHASNIE VRMNHPT--- ---------- ---LPKPIMV AAHYTAIGET  1822
M. ensis apoCr1                VHEN------ ---------- ---------- RWGERETKWE AKMNHPV--- ---------- ---LSRPIMV AVKYTASEET  1825
M. ensis apoCr2                THEG------ ---------- ---------- RRG----RWE SRINHPV--- ---------- ---LPRPIQV AVQYSGSERT  1805
C. quadricarinatus apoCr       SHN------- ---------- ---------- -YWDQVSKYE GRLSHPG--- ---------- ---MSKDIRV KVEHSYSGQT  1815
M. rosenbergii apoCr           SHS------- ---------- ---------- DWSGE-SRYE GRVSRPG--- ---------- ---LERDRRI DIQLRREEQK  1773
P. hypsinotus apoCr            TPS------- ---------- ---------- EWNQH-SRFE AHVSHPS--- ---------- ---MQRDLQV DAQYTRNEQR  1769
P. trituberculatus apoCr       IDS------- ---------- ---------- LWGGN-SKLQ GKMSHPM--- ---------- ---LSRDMTA VIEYTRSGSG  1794
C. feriatus apoCr              MDS------- ---------- ---------- MWGGN-SRLQ GRMSHPK--- ---------- ---LSRDMTA AVESTRSGSG  1798
D. melanogaster apoLpII/I      TSSTIKLALP KRQILFETTQ KIPADGSLFG RYEQTASFFI DKLQKPDDVA RFSAIVDVTG TERVAFNANG KLKFEHPTIR  2209
A. mellifera apoLpII/I         KEAATILTLP SREVALILIY DVPKT-RQTA AYKLDVSLYL DRKNKPSEKT SLSAIGDINV D-KNSLSLSG ETKFTYPTQH  2036
L. migratoria apoLpII/I        SGSGFTLTLP SRVIAAEVKL SSDVK-PNHS KQQISASVWL DKTRLPNSFS SVSILLEEIE D-KNTDKYVS QLRFTHPNLE  2225
A. gambiae apoLpII/I           TSAGASLILP TRTVAVEGVL NLPKD-KFG- PFDGSVSFYL DKKNEPDNKA TFAVRGETKM LGSTGVSANG ALTFSHPTIK  2140
M. sexta apoLpII/I             ENG------- ---------- ---------- -HY-ASG--V TVRLPS---- ---------- -----RVMAL EYTLTHPTSQ  2164

P. semisulcatus apoCr          MK-GTIELDI FPEAANKITG -TLETQRISE NAIRAEAFLT GRMLQVNPK- --AIITAAYA PETVALDVVF HK----TPSA  1893
F. merguiensis apoCr           TK-GTIELDI FPEEANKITG -SLETQRISE NAIRAEAFLT SRMLKVNPK- --AIITAAYA PETVAFDVVF HK----TPSA  1892
L. vannamei apoCr              MR-GTIELDI FPEEADKITG -TVETQRISE NAIRAEVFLT GRMLKVNPK- --AIITAAYA PETVALDVVF HK----TPSA  1866
P. monodon apoCr               TK-GTIELDI FPEEADKITG -TLETHRISE NAIRAEAFLT GRMLKVNPK- --AIITVAYA PKTVALDVVF PQGLPRSPSA  1890
M. japonicus apoCr             IK-GTIELDI FPEEENKITG -TLETQRISE NAIRVEVFLT GKILQVNPK- --AIVTVAYA PETFALDVVF HK----TPST  1893
M. ensis apoCr1                AK-GTIELDI FPEEENKITG -TVETTRIAE DAIRAEVFLR GRLLKVNPK- --AAITVAYA PNTLGFDVVL HK----TPSE  1896
M. ensis apoCr2                VE-GSLELDI FSASEDKITG -TLRSTRVAE NSVRTEIDIE SRILNASPK- --VILTTAIA SDTLALDVEL KK----APSA  1876
C. quadricarinatus apoCr       MR-GSLELDI FPDTEDKITG -TLKSTMIAN NTVRIEASLT TRILRVHPK- --VTVMAAYS QNTTGIDVQF QK----SPSS  1886
M. rosenbergii apoCr           VT-GTVELDI FLRPEDKITG -RLESLIYAR NTVIVEAKLE GKVLKDHPK- --VVVRGAYG QSNVGFDIKF HK----NHSS  1844
P. hypsinotus apoCr            IA-GSIELDI FPNPEDKITG -KLESMILSK NTIIIDAQLT GKALKVNPK- --VTVAAAYG PRTTGFDIQF HK----TPSS  1840
P. trituberculatus apoCr       QQ-GSFELDV FPDAADKITG -SLTSVLRAN NTIAIEANLS SRVLQVKPR- --VMMEVSWA AHTAAFNFTF HE----TPSS  1865
C. feriatus apoCr              HQ-GSFELDV FPDTADKITG -SLTSILRAN NTIVIEANLF SRVLQVKPK- --VMMEVSWA AHTAAFNFTF QE----TPLS  1869
D. melanogaster apoLpII/I      P--LSISGQL NGDVNQQIAS AEVIFDIFRL PEQKVVGNSE LRNSRSQNG- --FNIAYITT VKSAGLQFQY QIN----SNA  2280
A. mellifera apoLpII/I         KD-MSMKGYL HYGGDKLLDA -NLDIDVFAK RSQKISIVAN VQRQQIPNG- --QNVTSLVE VNSRGQQLKL DLKS-HLAIS  2110
L. migratoria apoLpII/I        KD-LTVKGYV QIGLENKLFD SNLEIDIFKQ KNQKISISST VVEQKQNDV- --VKYLSTLD VKSKGHELDV TGRG-EATVK  2300
A. gambiae apoLpII/I           AL-AIRAKGT LDGDKQTADG -SVEFDVFKK AGDKIVAKVR YANSDQSFKG FNITTEASVS SKGLGLKCGF SG----HSAM  2214
M. sexta apoLpII/I             DLPFPIKGEA CLDLDKNRPG HKTSARFLVD YSNSGSEDKA VAEIGFFHP- ------KIEK EAVIRLNAFM KR-----PEN  2232

P. semisulcatus apoCr          APIFAIAAKY DKTAAHSAAA TFTVKMEERP VFEITAVTEP EDAATCNGIR MKAIAYAATF G-KYNIFSKM CRPAFIEVTA  1972
F. merguiensis apoCr           APIFAIAAKY DKTAAHNAAA TFTVKMEERP VFEITAVTEP EEAATCNGIR MKAIAYAATF G-KYNIISKM CRPAFIEVTA  1971
L. vannamei apoCr              APIFAIAAKY DKTAAHSAAA TLTVKMEERP VFEMSAVTEP EEAATCNGIR MNAVAYAAAF G-KYNVFSKM CRPAFIEVTA  1945
P. monodon apoCr               APIFAIAAKY DKTEAHNAAA TITVKMEERP VFEITAVTEP EEAATCNGIR MKAVAYAATF G-KYNIFSKM CRPAFIEVTA  1969
M. japonicus apoCr             APVFALAAKY DKTSAHNAAA TFTVEMEQRP VFEITAVAEP EEEVPCNGIR IKAIANAPAF G-KYNIFSKM CKPAFIELTT  1972
M. ensis apoCr1                APVFKVSAKY DEIAARTAAI AFTVKMEGRP VFEVSGATEP EEEAFCNGIR MNAIMYAAAL G-KYDVISKM CKPAFAEVTV  1975
M. ensis apoCr2                APVLKAFAKY DKTTPRNAVF AVNVEVKNAP VFELSGVMKP EAVPECGGVA FSAVAYTPAL G-KYDIRSKM CKPAFVEVAV  1955
C. quadricarinatus apoCr       PVSFQVSALY DRIFTGDATM TFRVINEEDA VVDIAGVMGP EKDPECNGVN IKGVAYASPI G-SYDIRSKL CRPFFFELIS  1965
M. rosenbergii apoCr           PTTFRMSGRY DKRSGRNAAA SFIIENENQK AIDISVALHP HQSSLCYGVR AEAKAKSSLI G-TYDIYSEL CKPGFVSLTT  1923
P. hypsinotus apoCr            PVSFLMSGKL DMSYGRVAAT SFIVKTEGRT VMDITGSVQP HQTPECYGIR IGAKTHSSVI G-TYDVSTKI CKPAFIEVIT  1919
P. trituberculatus apoCr       PESLKVYAKY DRISKNYAAV TFHLASEGRP TMDVSGVIEP RYGPHCDGFA LLANARTSLL G-DFAVNSTV CKPVFLEVVM  1944
C. feriatus apoCr              PESLKEYAKY DRISRNHAAV TFQLAGEGSS TMDVSGVIEP RNGPHCAGFA LLANARTSLL G-NFAVNSTV CKPMFLEVVF  1948
D. melanogaster apoLpII/I      AVDIEAHEYN IGLELNNGEI DVKAISFLNK EKFEISLSES NKHIIYIVGD FSKQNHYAKL N--TKVQILD KNPIEITSEV  2358
A. mellifera apoLpII/I         KKQIGFGTFF TYNNVKQKPK TLGALYSADL NHVSLLITLP DKQLIRDNWK FDITRNTQKV HRELSLLDNT PQVLNFEAND  2190
L. migratoria apoLpII/I        PSLIALQSVL KYKKDKRIKE FKNFQFEVST EKLLVHVKVP NHHLLHIDAN TKINDKHASG DASVHIIGLP TSVIHIEGEN  2380
A. gambiae apoLpII/I           SLATRQASAA GSLTLPFEGY TFGSYFFGSP ESFDFLLTRF GDDFVRAHGT YDAKKYRGDL T--STFKFLP NRPVVFESQL  2292
M. sexta apoLpII/I             GCFKIESSAS LCHSALGTDR VAKVMFETTP NSVKFLADTP FVKAIDVEGS FNVNQQQRTQ QCLFRICLLE GKPVQMSALV  2312
```

```
P. semisulcatus apoCr        MRPGGAKEYI AK-------F GLRHPDAAEA GVYVASGRAG ETHGVAVAAV KLASPTMLKV EMAYEPQEAE AIINEMTEEF 2045
F. merguiensis apoCr         MRPGGAKEYI AK-------L GLRYPDAAEA GVYVASGRAG ETHGVAVAAV KLASPTMLKV EMAYEPQEAE AIINEMTEEF 2044
L. vannamei apoCr            MRPGGAKEYT AK-------L GLRYPDAAEA GVYVTSGRAG ESRGVAVAAV KLASPKVLKV EMAYEPQEAE AISIEMTEGF 2018
P. monodon apoCr             MRPGAAKEYI AK-------L GLRYPDTAEA GVYAASGRAG ETRGVAVAAV KLASPTMLKV EMTHEPEEAQ IVMSEVSGNL 2042
M. japonicus apoCr           MRHGGEKEYT AR-------L GLRYPDTAEA GVYVASGRAE EIRGVAVAAV KLASPTMLKV EMAYGPEEAQ VLMNEMTEEY 2045
M. ensis apoCr1              RRHSSEEEYI AK-------I GLRYPDTAEV GLYEASVETG ERRPVAVAAV ELASPTMMKV N--------- ----EVTEAL 2035
M. ensis apoCr2              GREGAVKEYI AR-------I GLQYPDNAEI SMSEGSTDSE EETPLAVARL TLVNPEVINV DLAYE---RE GIFRMKEEVM 2025
C. quadricarinatus apoCr     KKQESQKEFI TK-------L GLQCPNRAEI SLSESNLDQP WRNAIAMARD KLPSPTVAEV HFVYESENMH TVKGALKEDW 2038
M. rosenbergii apoCr         KKHGSDRMYI TK-------L GMQGMKNVEA SILEANPRTM EKRPLGMARM VLTSPTMMKL ETKYEGDKLH EIKSTVGDML 1996
P. hypsinotus apoCr          KKHGSARMYV TK-------L GVQGMKNVEI SIYEADPETE ERQTLGMVRV RLSSPSLMKV ETVYKGDHLR SIKSAVYENW 1992
P. trituberculatus apoCr     KKQNSENIYK AA-------L GLQLPYKAQI SLSESDRSQT WDKYISLVDI RLETPRHIKI DYEYRENEMA ALKDLVLNDV 2017
C. feriatus apoCr            KKQNSEKIYK AA-------I GKLFPYKAEI SLAESDRYQT WYKYISLIGI RLETPRRIKI ASEYRENEMA SLKDLVLNDV 2021
D. melanogaster apoLpII/I    QPNSAKIILK RQ-------D FIDGTAEVKL GKEFKVDVIG SGKQLFNGRV ALDATNFLQT NYFINEDHLN GFWHIVESEI 2431
A. mellifera apoLpII/I       FNRFKLETYL KNNPNNKAVL NGQMVLGQLA EIHAHWFKDG VKKHLFHALV NLDEKQFLKP DFGYNTENIA ELGKVIKNKN 2270
L. migratoria apoLpII/I      KGFPVVKGTI SSEGTPN--K LELIADLSDG LLVEADFISE SGKKELFYTF LSGKKDSRKP EFRWSVENIQ SALEPHKNDI 2458
A. gambiae apoLpII/I         NGLSSAKFAF KQD------Q FFSADGTFGV DKAMVLKVMG EGKPLLNAKV TLDASHFLST EYNVDEANAK AFLVSLKNQL 2366
M. sexta apoLpII/I           KDYQYYEFTT EES-NRKLSY VGHLIPEKRV DISTDIILSG DKKNIAHGAL FLQDN-LVKS DYGLSKENFN YFLNALKKDL 2390
```

```
P. semisulcatus apoCr        EKIAASFTSV VMEVVQFLKE EAA--AKGVH FPSSELVNLL GVAKEEIEEI CRDIVSEARI FDTEILGDIL GSPVVSFISR 2123
F. merguiensis apoCr         EKIAASFTSV EMEVVQFLKE EAA--AKGIQ FPSSQLVNLL GVAKEEIAEI YRDIVSEARI FDTEILGDIL GSPVVSFISR 2122
L. vannamei apoCr            EKIAVSFKSV AIEFVQFLKE EAA--AKGVQ FSSSQLVNLM GVAKEEIVEI YRDILSEARI FHTEILANIL ESPVVSVISR 2096
P. monodon apoCr             GQVAMSLKAV VMEVVQFLKE EAA--AKGVE FPSSQLVSLV DEAKEEIKAI YRDIGREMRI LDTELIADIL DSPAVSFMSR 2120
M. japonicus apoCr           EKAAVLFKSV VMEVVHFLEE EAS--AKGIH FPSSQLVTLL GVAKEEIEEI YRDILSDARI FDTEIIRDIL ASPVVSFVPR 2123
M. ensis apoCr1              ETIIMAIKPV AMNTFRYLEE EAA--EKGVQ FPSAHFIALM SEAKAEAEAI YREILEEVRM LDTEVIEEIL ASPAVSFIWR 2113
M. ensis apoCr2              AQLVETMESV EAEAKDISEE VTE--GRFDG IPATELGALV SEATRELKMM YRDLVEEG-- -VAPMLREIP SFGRYGNGLK 2100
C. quadricarinatus apoCr     QRVMESAHSW ADSVSRYLEE QAQ--QQGTT FPNPEIETLL EEVKHDLREI YHDLIYKEII PHYEAFREFL RRPPASYVIQ 2116
M. rosenbergii apoCr         ERLMSSAGNW MDSMSSEVLQ EG------SG SPSGHMSSVW QEIKNEASRI YSDLENDLVI PKYDRLREWA RGGLLSNVAE 2070
P. hypsinotus apoCr          SSLTSSAGSW LDDMCREVIR EGG-----SS SPSAQMAKLW QEIKAEASRI YEDLEYDCVI PSFEKIKQLA RSDIVRNVAS 2067
P. trituberculatus apoCr     YRITEEVLSW TDRVYAEIER QAS--QAGVP FPTPKIRQLM QEIKQEIVEI YRDLIYNDIL YEWNALVEIL HGPTATFIKK 2095
C. feriatus apoCr            YRITEAVLTW TDRVCNEIER QAR--QKGVP FPTPQIHQLL QEISHDIGEI YLDLIYNDIL YEWEALKQLL QGPTATFVKE 2099
D. melanogaster apoLpII/I    NKDSEYISEN IKERLKKSRQ VTDKIVKLAK EAGPDFSKLQ GKLLDYKNDI VQELEADQSI APIIDGIRTL FKKIAGIVDD 2511
A. mellifera apoLpII/I       LDVIKDAKDV YGYVLDETSA EGSDFVDHLV KAKPSFQSLV EYYEKELNKL KEEMNADET- IQEIQVTLIC GHQMAEKILD 2349
L. migratoria apoLpII/I      QEVLNKLKEI SDEAGNEITK ESSRLADSLK AGLPNFRRFV NTYETQLKAL KEEIANDKVL KEISENWKEV IGDAAEVVST 2538
A. gambiae apoLpII/I         NADFEVTRAD VSQRYAKLAE ELNKLSTNLV SALPEFGKFQ ESYAKQLQKL QEDIMSDPAL AEFVKAATKI FQQVSEVFGQ 2446
M. sexta apoLpII/I           DTLEDRIKNV GEKASKDVEA VTQR------ -AAPYFKKVE DNFRAEWNRF YQEIADDKVF KEISHVFNEI VQYIAKFIDE 2463
```

```
P. semisulcatus apoCr        VYFGVWSEMI RLQHHLSVSL IQTIERFQEE LAGISEIVME VVMTAARMAE TG-------- -EVPEVVFDA FEEIR--TAK 2192
F. merguiensis apoCr         VYFGVWSEII HLQHHLSVSL IQTIERFQEE LGGISEILME VVMTAARMAE TG-------- -EVPGVVFDA LEEIK--ATK 2191
L. vannamei apoCr            VYFGVRSEIV RLQHQLSVTL IQAIEGGQEE LAIVYEIVME VVMTAARMAE TG-------- -EVPVAVLEA LEEIK--ASK 2165
P. monodon apoCr             VYLGVWSHMT HLQHHFSTSL VEVIQKWQEQ LRDVSEVLME YVMEVVQLLE AG-------- -EVPEVVFDA LEEIE--ATK 2189
M. japonicus apoCr           VYFGVWSEIV LLQHQLSVNI IQAIERFQGE FEGITEIIME IVMEATRMAE TG-------- -EVPKVLLDV LEQIR--ASK 2192
M. ensis apoCr1              TYFDVWSQMA RVHHKITAAI IRAVERYEEK LEGVTEMIME IVMATSRMVE TG-------- -ELPEAVLDV VEEIK--ETE 2182
M. ensis apoCr2              ALVRFWAQMQ NSVLEERE-- -RMLSVCQEI VADATEKVFT LMNEAIAIVE TG-------- -EMPDSVRQF MDMVK--ENP 2166
C. quadricarinatus apoCr     FSSSILSGIA KIQRDLRSRL LHEVLAWQEE FKDITERIIE LLVKATRWVE TG-------- -EIPEPVRRL LEQLQ--ETR 2185
M. rosenbergii apoCr         GCSKVWSHYA HVQNSLSSSV SNMIRTIREE FPGLTRVVTE VVMGTARGLQ TG-------- -EMPEVFRRW WNEFL--ESS 2139
P. hypsinotus apoCr          GYFNLWSHYV QIQHRMASSV SNAVRTMQSE FDDIAKIIIE AVMGSARSLQ TG-------- -EMPEGIRSL WSQMR--ETA 2136
P. trituberculatus apoCr     SIFQSLQSMA QLQREWAVS- --IVEQLKQH FKPAINKISE AVKQVDQWMQ TG-------- -EEPEIVRRL VEEVE--RSA 2161
C. feriatus apoCr            SIFQLLQSMA HMEREFAVY- ------IMQQ FKPAINKIIE AVKEVARWVQ TG-------- -EEPEIVRRL VEDLE--RSA 2161
D. melanogaster apoLpII/I    INKAISEILE KAQKSIVDIY DKLQALWKDS LLKAWEDFII TVQKLISTLK TEFIK----- -ICTQSFKDL LSALEKYGPA 2585
A. mellifera apoLpII/I       ILRKYFGTLF EILTETMKRI AHGLEKLKES LNNLISNVKQ AVNSMYPKLK ESYDKIFHQM LEILDAVIKL ANTYL--QAV 2427
L. migratoria apoLpII/I      LVNGILVTID ALLKTLNELA ESVLDALKKS LPALKDSYKQ AVDAIVGIAK SLTQSLVNIL SSAAEILKKH EADIKGYLSV 2618
A. gambiae apoLpII/I         LSQVYVESFR KMSALVNDVV AQLMETFNTK VLPALKELST KVEAIFFNVY EET------V KLVVAVFERT VKALKVFEED 2520
M. sexta apoLpII/I           ILQGTKRSWT PSCRPTLSHP RNR-EMYKKQ IEPQVKQLYD TLGALMKEYL DG-------- --VIDVVAHF AAIVT----- 2527
```

```
P. semisulcatus apoCr        VFRIVKR--- ----EVEAIL EEYPEEYEAV KHIVYNVVAI LKRDVGIIRE RLMEIPAVLK IIDYTMYHFH SER-------  2258
F. merguiensis apoCr         VFRIVKR--- ----EVDAIL EEYPEEYEAV KHIVHNVVAI LKRDVGIVRE RLMEIPAVLK IIDYTMYHFH SER-------  2257
L. vannamei apoCr            AFRVVKR--- ----EVDVIL REYPEEYEAV KHIFGNVVAI LKRDVGIVRE WLMEIPAVQR VIDYTMYHFH SER-------  2231
P. monodon apoCr             VFRVVKR--- ----EVDAIL EEYPEGYDAV KHIVDNVVAM LKRDIGIVRE RLMEIPAVVQ IIDYTMYHFH SER-------  2255
M. japonicus apoCr           VFRIVKR--- ----EVYEIL EEYPEEYEAI THVVGNVMAM LERDVEIVRV GLMKMPAVQR IIDYIMNHFH SKQ-------  2258
M. ensis apoCr1              VFRIVKR--- ----EVNAVL REYPEEYDAI KHVVFKVAEA FARDIVILRE RIMEIPAVER TINWAIRQFT SER-------  2248
M. ensis apoCr2              VFKILRQ--- ----KLDAVM EEYPEEYKTI KYVFFRVKET FERDIDLLFE RAMEIPAVQR FINWIMENLT PGR-------  2232
C. quadricarinatus apoCr     IFRMFKR--- ----DVDAFI RRYPEEYEAI QEMVAKVKDT LQEDFENVLI RISKIKVVEN TIKWILKDLS NEN-------  2251
M. rosenbergii apoCr         FCRAIES--- ----DFDPLW NEYQEEYQGL QQIWRKVKNT LTKDVNRQRR NLMHYKKPRH LVNWIVSDMN IER-------  2205
P. hypsinotus apoCr          IYRTVQR--- ----ELDSML SQYPEEYQGL QQMLEKVKST LATDFAKQRH NLMQHSKPRH MINWINNHLN FDR-------  2202
P. trituberculatus apoCr     IYRLLQTE-- ----IIQPIK ETYPQQYQVT VDVVAKVIYT LRHDLLMTKH KVLASPTLSR VIR-KIADLS QDN-------  2227
C. feriatus apoCr            IYQIIQTH-- ----IIEPIK ETYPQQYQAT KEVVAKVIDT FRHDLLIMQH KVLASPTLHR IIR-KIEDLS QPD-------  2227
D. melanogaster apoLpII/I    LKNYGKA--- -----IGEIV KPINDAAQEV IKIVVNAAEG VTHEFKQYVA SLPSFESIRN EFNDKVKVLK LIEKATELTN  2657
A. mellifera apoLpII/I       LNLINEHQ-- ---KEIKDML NVISGMSQDI VKILFKGLEQ IKLNLDQFCH LLINQLKALP AYETIKERLE ELKNFQIPDN  2502
L. migratoria apoLpII/I      LADLANDVGK FVTKITGVIY EGVVEFSKPI KEKLDGLKFG VAIEFGKVVE QLQNLIVPQE LLAFAQEIVS ELKET-----  2693
A. gambiae apoLpII/I         FNKIATS--- -----VSELF RTFAQTFSKA VQVLEKELKE LYKLVQEYFD TFDEFKAVKE TFKEYFDGFD RYAYQLLKEL  2592
M. sexta apoLpII/I           -DFFEKHKA- ----ELQELT NVFTEIFKDL TRLVVAQLKE LPPKIAQIYN DIVSQITNMP FVVVLQEKWK EFN--FAERA  2599

P. semisulcatus apoCr        ---------- ----ALAAEA EKVVSLILNE LLFVS-MERE GNG---IEVR IPLHRPLYSL TQVAQEAVPN --------PV  2312
F. merguiensis apoCr         ---------- ----AFAAEA EKLVSLMLNE LLFVS-MERE GNG---VAVR IPLHRPLYSL TQVAQEAVPN --------PV  2311
L. vannamei apoCr            ---------- ----AFAAEA EKVVRLILDE LLFVS-MESE GNG---VAVR IPLHRPFYSL TQVAQEAVPS --------PV  2285
P. monodon apoCr             ---------- ----AFAAEA EKIVSLMLNE LLFVS-VERE GNG---IAVQ IPLHRPLYSL MQVAQDAVPN --------PV  2309
M. japonicus apoCr           ---------- ----VFAVEA ERVVSLILSE LLYVS-IERE GNG---IEVQ IPLHRPLYSL TQVAQEAVPN --------PI  2312
M. ensis apoCr1              ---------- ----LIVEQA DKIADFLLRE LLFVS-VEAE SNG---FELQ IPLHRPLYSL TQVAQEAVPF --------PT  2302
M. ensis apoCr2              ---------- ----LAAEEA EVLAETLLQD FHVFT-VKSE GNQ---IKIE IPLQKPLYLV VQFIKETIS- --------PY  2285
C. quadricarinatus apoCr     ---------- ----MIANKV EEYISEIIQE AIWALGVETN ESE---MKFK MHLHKPVYSL IQLLQEVHLT --------PI  2306
M. rosenbergii apoCr         ---------- ----MVFRGV DKVIKNVVQK ALIVP-VQID GSH---FQLQ LPIRRPVQSL PQALSYVSLN --------PT  2259
P. hypsinotus apoCr          ---------- ----MMFQCM DRLVKNVVQN ALFLS-VQME GND---VQVQ LPIRRPVYSL PQAFSYASMS --------PV  2256
P. trituberculatus apoCr     ---------- ----TLRQTL EWLESQLMQS VIIIA-PEPG VNH---VGIQ IPLYRPIYSL SQVVNMTMQM ------QSPL  2283
C. feriatus apoCr            ---------- ----TMRQTL EWWESQVVQS LVTIA-PEPG VYH---VGIQ IPLYRPIYSL TQLVNMTMQS --------PP  2281
D. melanogaster apoLpII/I    -----SLFDQ INILPQTPET SEFLQKLHDY LIAKLKQEHI DNEKYIEELG QLLIKAVRSI WVSIRSTYPG SSDHVIDFQS  2732
A. mellifera apoLpII/I       ILNSLEELCK LGKNILPTEE LRHFVDITCE YIIKLVKRQK IND---MNEL KKIYSSLVAA VQSIVALAQK QSSLENIWGL  2579
L. migratoria apoLpII/I      ---------- ----TLTPEI QDLLQAIEKY LEKVSKKKDA DVE---KELK LIFEKAIDAA ESVINFVVSE ITGGDHTKDL  2756
A. gambiae apoLpII/I         LS-------L VEAVYPMPEV TELTTAINKY ITSKLDNKPV NDV---EELK TLFVSLVKVL NQAVERLIAG -VNVQLSEPT  2661
M. sexta apoLpII/I           VQLVSQAYEA FSKILPTDEL KEFAKALNAY LLKKIKEEKM EES---KELP RAVREAGQRV LLITSIPALA VRR-PRLRRW  2675

P. semisulcatus apoCr        TMLEN-LIFA YVEYIPIPVS DAIWAFYNFV PHYIT---DV LPPYP----- ---------R TATVVGGTEI LTFSGLVVRA  2374
F. merguiensis apoCr         TMLEN-LIFA YVDYIPIPVS DAIWAFYNLV PRYIT---DV LPPYP----- ---------R TATVVGGSEI LTFSGLVVRA  2373
L. vannamei apoCr            TMLEN-LIFA YLEYIPIPVS DAIWAYYNFL PRYIT---DA LPPYP----- ---------R TAMVVGGTEI LSFSGLVVRA  2347
P. monodon apoCr             TMLEN-LIFA YVEYIPIPVS DAIWAYYNFV PRYIT---DV LPPYP----- ---------R TATVVGGTEI LTFSGHVVRA  2371
M. japonicus apoCr           TMLEN-LIFA YLEYIPIPVE HAIWAYYNFI PRYIT---DV LPPYP----- ---------R TAMVVGGSEI LTFDGLVVRA  2374
M. ensis apoCr1              LMLEN-LLWA YTEYIPIPVE DLIWAYYNFV PRHITEMTDM LPPFP----- ---------R VAMVVGGTEI LTFDGLVVRA  2367
M. ensis apoCr2              ANTRD-VMWV IDSSLPYTLE DVIWAYYTFI PRHIT---EL LPPYP----- ---------R TAMVVGGTEI LTFDGLVVRA  2347
C. quadricarinatus apoCr     YYLEK-LALV HDRLIPFPIN NVIWAYYTLL PRHMT---EL LPPYN----- ---------R TAMVVSDTEI LTFDGALLRA  2368
M. rosenbergii apoCr         PVVDR-ALWW LEALMPTPID NILWAHYKFL PRHAR---YL LPPYN----- ---------H TAMVVDGSEI LTFDGVVLRV  2321
P. hypsinotus apoCr          PAIDT-ALWS FEALMPTPVD NLIWAYYSFM PRHAR---YL VPPYN----- ---------R TAMVVDGTEI LTFDGAVLRA  2318
P. trituberculatus apoCr     TLTEK-MLLS AEAFNPISVR KILETYNRWV PRNLS---AL PLSLN----- ---------Q SALVVGDTEI LTFDGVLLRM  2345
C. feriatus apoCr            TLTQK-ILLS AESFRPIPVT KIMETYYDWV PRNLS---TL PLSLN----- ---------R SALGVSDTEI LTFDGVLLRM  2343
D. melanogaster apoLpII/I    WIGSLTHSFD SLAVLPSILS FRSSILNCLL NENWDVVFNK KLLYSWIFFN ------DFEL RGHVVDGKHI FTFDGLNFAY  2806
A. mellifera apoLpII/I       ISIQT-PDLG LLSKLPTISA LKLSVWNLLR NRELPTLEDL YYTYRPTPL- FR-------K SGVVTDGGHF FTFDGRHLTM  2650
L. migratoria apoLpII/I      YDINIPTDLP SFIQLPRVFS VRFSPLIYLV SNGVPCLSDL LASYRPSLR- FDNIIPPYDA TAILLNSHHF FTFDRRHLTF  2835
A. gambiae apoLpII/I         FGSDSFTSFV TFKFLPYVSS IQFSPWNFVR NEKFYSVRDL IHQLRLYAFN PFARVPMFHM HAQLGDGGHF FTFDDKHFTF  2741
M. sexta apoLpII/I           TWHHLKLAVG AGASAPSLGA ASWSALRQLA AGDGP--PAL APRGLPTAQL DPLDEVPNKL RAVVVNGQHI FTFDGRHLTF  2753
```

```
                                                                 VWD domain
P. semisulcatus apoCr        PRSPCKVLLA AHG------- ---------- -----SHRLM MSHPQASAPA QLELKTPA-A TVVIKPDFEV VVNGQPLGGS  2431
F. merguiensis apoCr         PRSPCKVVLA AHG------- ---------- -----SHRLM MSHPQASAPA QFELKTPA-A TVMIKPDFEV VVNGQPLAGS  2430
L. vannamei apoCr            PRSPCKLLLA AHG------- ---------- -----SHRLI MSHPQASAPA QLELKTPA-A TVIIKPDFEV LVNGQALGGS  2404
P. monodon apoCr             PRSPCKVLLA AHG------- ---------- -----SHRLM MSHPQASAPA QLELKTPA-A TVMIKPDFEV VVNGQPLAGS  2428
M. japonicus apoCr           PRSPCKVLLA AHG------- ---------- -----SHRLM MSHPQPSAPP QLELNTPA-A SVVIKPDFEV LVNGRPLTGS  2431
M. ensis apoCr1              PRSPCKVLLA AHGR------ ---------- -----THRLI MSHPQASARP QFELKTPE-A TVVIKPDFEV VVNGRPLSSS  2425
M. ensis apoCr2              PRSPCKVLLA AHG------- ---------- -----SHRLI MSHPEATARP ELELKTPQ-A TVEIKSDLQV FVNGQPMRRS  2404
C. quadricarinatus apoCr     PRSPCHVVLA VYA------- ---------- -----NNKLT MTHPQPSAPP QITFSTGS-T TVSVKPDFRV DVNGHEMNRQ  2425
M. rosenbergii apoCr         PHSPCKVLLA QYK------- ---------- -----THSLV MQNQQPGHLP HFILKAAG-A TVEVKPEFVV TVNGNPVSGP  2378
P. hypsinotus apoCr          PRSSCKVLLA QHK------- ---------- -----SDSLV MQNQVPAQLP HFFLKASG-V TVEVKPDFTV TMNGQPISGP  2375
P. trituberculatus apoCr     PRSSCKVLLA SVP------- ---------- ----DVVSIY MSHPQPSQGP EVILQAGS-T KAIIKPNLEV DVNGQQVHG-  2402
C. feriatus apoCr            PRSPCKVLLG PDGGSRYTWS RMLAHPVRCY CFWADVVPVY MSHPQPSQAP GVPLQGGS-T KAIMKPNMEV NVNGRPVHG-  2421
D. melanogaster apoLpII/I    PGNCKYILAQ DSVD------ ---------- -----NNFTI IGQLTNGKLK SITLIDREGS YFEVADNLAL KLNGNLVEYP  2865
A. mellifera apoLpII/I       AGSCTYILAQ DMQD------ ---------- -----GNFSV VANFNNGILI SVTLTEPK-E SIAIKNNGNI LVNNKPADYP  2708
L. migratoria apoLpII/I      KGICSYILAQ DVQD------ ---------- -----GNFTI IANIEGGSLK SIIVSDQA-T TFELASDKSL LVNGKPTEYP  2893
A. gambiae apoLpII/I         AGSCSYLLAS DLVD------ ---------- -----GNFSI VADMDGGRLK SVTLVDKD-S TVELTAKAVV KYNGKETDLP  2799
M. sexta apoLpII/I           PGTCRYVLIH DHVD------ ---------- -----RNFTV LMQLANGQPK ALVLEDKSGT IIELKDNGQV ILNCQSHGFP  2812

                                                        VWD domain
P. semisulcatus apoCr        QQTIG-NVRI VNT-AKHIEV GCPVMKVVVA KEGEAVAVEA SGWVFGRVAG LLGPNNGEIA HDRFMPSGAA ASNPRDLVAA  2509
F. merguiensis apoCr         QQTIG-NVRI VNT-AEHIEV GCPLMKVVVA KAGEAVAVEA SGWVFGRVAG LLGPNNGEIA HDRLMPSGAA ASNPRDLVAA  2508
L. vannamei apoCr            QQTIG-NVRI VNT-AKHIEV GCPLMRVIVA KAGEAVAVEA SGWIFGRVAG LLGPNTGEIA NDRLMPSGAA ASNPRDLVAA  2482
P. monodon apoCr             QQTFG-NVRI VNT-AKHIEV GCPLMKVVVS KAGEAIAVEA SGWVFGRVAG LLGPNNGEIA DDLLVPSGAA ASNPRDLIAA  2506
M. japonicus apoCr           QQTIG-NIRI VNA-AKHIEV GCPLMKVVVA KTGQVVAVEA SGWTYGRVAG LLGPNTGEIA DDRLMPTGVQ ASSPRELVSA  2509
M. ensis apoCr1              EETVG-KLRI ENI-GEYIEV ASPVMKVIVA KSGEVVAVEA SGWLFGRVAG LLGPNNGEVA DNLLMPNGEA TPNPRELVAA  2503
M. ensis apoCr2              EATIG-QLRI ENR-AGHIEV ACPLMKVIGE KAGEVVAVKA SGWTFGRVAG LLGPNNGEIG DDRLKPTGEA ASSPPELVAA  2482
C. quadricarinatus apoCr     QLTAG-DVFI QKT-SREVNA TSPFMTVRVF RQERVVSVNV SGWTYGRIAG LLGTYDGEVG NDWFTPSGRN ASSLQELVAS  2503
M. rosenbergii apoCr         REVQG-EVVI VKE-NEKIKV RTPFITLRVY KDSRTASVEV SGWTFGRIAG LLGTYDGEKA TDRMTPQGTR ASNLQELVKS  2456
P. hypsinotus apoCr          EKVQG-EVKI FKR-AEKIEV MTPFMTLRVY KMSHTASVEV SGWTFGQLAG LLGTYDGEMG NDWMTPSGSR ASTLQELVRS  2453
P. trituberculatus apoCr     RQTVG-DLVI EVN-PHRVTV VSPMLGVQLM KEQRVVIVNA STWVFNHTRG LLGLYDHERA NDRMMSNGRN ASSLHDLVNS  2480
C. feriatus apoCr            RQTVG-DVVV KVS-PDRVAL VSPILGVQFM KQEHVVMVNA STWVFNHTRG LLGLYDNERA NDRMMSNGRN ASSLHDLVNS  2499
D. melanogaster apoLpII/I    QHLSGLHAWR RFY-TIHLYS EYGVGIVCTS D-LKVCHINI NGFYTSKTRG LLGNGNAEPY DDFLLIDGTL AENSAALGND  2943
A. mellifera apoLpII/I       AHTKNLHAYL FPP-YGNIKS DYGVRVSCTS KAPMICAVHV SGFYHGKLRG ILGDANNEPY DDYTLPSGKI TESGTEFGNA  2787
L. migratoria apoLpII/I      ADEGEFHAWR EYN-RVGIQT KAGVKVTCET S-IELCTFEI NGFYFGKTRG LLGTINNEPW DDFTKPDGQV ASKANEFGNA  2971
A. gambiae apoLpII/I         IHQKDVYVFR KYY-TVTVGT KYGAQVMCTT D-LKICHFFV SGFYFGRLRG LLGNGNYEPY DDLAVPNGKI TEVSTDFANS  2877
M. sexta apoLpII/I           VVEQDVFAFR QTSGRIGLCS KYGLMAFCTS K-FEVCYFEV NGFYLGKLPG LLGDGNNEPY DDFRMPNGKI CSSESEFGNS  2891

P. semisulcatus apoCr        WQEDQQCSTP EVPRAE---T TV---ARLIQ CEALLGIRSR CNPVVHPQPF I-KMCHAAHK ---------- ACDAAQAYRT  2572
F. merguiensis apoCr         WQEDRQCSTP EVPRAE---T TV---ARLIQ CEALLGIRSR CNPVVHPQPF I-KMCHAAHK ---------- ACDAAQAYRT  2571
L. vannamei apoCr            WQEDPQCSTP EVPHAE---T TV---GR--- ---------- ---------- ---------- ---------- ----------  2503
P. monodon apoCr             WQEDQQCSIP EVPRAE---T SV---ARLIQ CEALLGIRSR CNPVVHPQPF I-KMCHAAHK ---------- PCDAAQAYRA  2569
M. japonicus apoCr           WQEDQGCSTP EVPRSE---T TV---ARLIQ CQTLLGIRSR CNPVVQPQPF I-NMCHAARN ---------- ACDAAQAYRT  2572
M. ensis apoCr1              WQEDQAVLHP EVSLVQR--T SV---VRVVE CETFLGIRSR CNPVVPPTPI HGDVSPPPTD ---------- AWDAAKAYRT  2568
M. ensis apoCr2              WKEDRQCSLP EVAEAR---P TV---ARVME CEALLLTRSK CIPMVRAEPF I-KMCHATRD ---------- ACDAAKAYRT  2545
C. quadricarinatus apoCr     WQEDRQCPTP PISPFDHDTV PA---ERIIQ CHSLLELRSK CFPVVNPKPF I-KMCHAAHR ---------- PCDAAKAYRT  2569
M. rosenbergii apoCr         WQENPQCETP PIAPANPMQV PV---VHMLH CQTLFGVRSS CNPIIRTEPF K-KMCFASRN ---------- ACHVAKAYRA  2522
P. hypsinotus apoCr          WQENQQCQTP SVSPASPLQT PV---VHMVK CNALFGVRSR CNPVVRQEPF M-KMCFASRN ---------- ACHVARAYSA  2519
P. trituberculatus apoCr     WQENPNCPTP SITPVDPMHV PV---KESVL CDFLFFQMRP CMPVVSPKPF L-QSCRIYSR ---------- PFEVIRSYQT  2546
C. feriatus apoCr            WQESPSCPTP SISPVDPTRV PL---KERVL CDFLFFQMKP CMPVVSHKPF L-QSCRIHSR ---------- PFEVAGSYHS  2565
D. melanogaster apoLpII/I    YGVGKCTAIE FDNNQFKSSK RE---EMCSE LFGIESTLAF NFITLDSRPY RKACDIALAK VAEKEK-EAT ACTFALAYGS  3019
A. mellifera apoLpII/I       YKLKSECPEA TAVEQHNERT PV---CTDYF TGENSPLKS- CFNIVKPSLY RDACDHAIAA GTPA-----G ACIIAMAYHY  2858
L. migratoria apoLpII/I      WKVDAQCANV DGVDHHEHSI KV---EECEE VFSKASLLSP CSLFLDPAPY LEACSHIAHE ATTKEEKQLA ACRTAAAYVQ  3048
A. gambiae apoLpII/I         YKTSQACAAV ADHGHDSHDH SS---PTCAK FFGSESSLKL CSYLRDQTGY KEACNHAAHD AG--EKADEA ACGIARLYVS  2952
M. sexta apoLpII/I           YRLSRSCPAA NAPAHDHHQM HAPLPKPCER VFSGTSPLRP LSLMLDIAPF RQACIHAVTG -ADADKDLQQ ACDLARGYRR  2970
```

```
P. semisulcatus apoCr        ICSLRGVGEV FPLAC----- ---------- ---------- ---------- ---------- ---------- ----------  2587
F. merguiensis apoCr         VCSLRGVGEV FPLGC----- ---------- ---------- ---------- ---------- ---------- ----------  2586
L. vannamei apoCr            ---------- ---------- ---------- ---------- ---------- ---------- ---------- ----------  2503
P. monodon apoCr             ICSLRGVEEV FPLAC----- ---------- ---------- ---------- ---------- ---------- ----------  2584
M. japonicus apoCr           ICALRGVEEM RPWAC----- ---------- ---------- ---------- ---------- ---------- ----------  2587
M. ensis apoCr1              ICALKGVEEV FPIAC----- ---------- ---------- ---------- ---------- ---------- ----------  2583
M. ensis apoCr2              ICAFKGVEEP TPFPC----- ---------- ---------- ---------- ---------- ---------- ----------  2560
C. quadricarinatus apoCr     MCARQGIRDM FPIPC----- ---------- ---------- ---------- ---------- ---------- ----------  2584
M. rosenbergii apoCr         MCETKGVKET FPLGC----- ---------- ---------- ---------- ---------- ---------- ----------  2537
P. hypsinotus apoCr          MCATKGVKEV FPLGC----- ---------- ---------- ---------- ---------- ---------- ----------  2534
P. trituberculatus apoCr     FCRTQGVMFP LSVF------ ---------- ---------- ---------- ---------- ---------- ----------  2560
C. feriatus apoCr            LCRAQGVMFP LSLF------ ---------- ---------- ---------- ---------- ---------- ----------  2579
D. melanogaster apoLpII/I    AVKQINKWVL LPPRCIKCAG PAG-QHDFGD EFTVKLPNNK VDVVFVVDIN VTPGVLS-NL IAPAINDIRE SLRSRGFSDV  3097
A. mellifera apoLpII/I       ACYAQGVMST YIPSSCTNCK VGGNKIDMGD SFSVKVPKKE ADVIFVIEQQ IPNDKVYKEM ITPLMSELRE ELKQQGVTDV  2938
L. migratoria apoLpII/I      ACSVENVFVS VPPHCVHCSV NGDAAIDIGQ SFSVKVPQKS ADILIVLEQV TGNAETVKDF VSPIVSQLTQ ELSSRGISDV  3128
A. gambiae apoLpII/I         ACTLYGIPTV LPSQCEKCST DEGRSVDLGD WYSVKAPQKK ADVVVVVDTS LGTLLG--EL VQSTINDLRK ELKATGISDV  3030
M. sexta apoLpII/I           SRSRGCCPPR CPTPACAART ATG-PGSWAT PTSTNCPTDS LISSSPLRPL RTTPAHYKNM VVPLVSQLVD MLKGKHCTDI  3049
```

```
P. semisulcatus apoCr        ---------- ---------- ---------- ---------- ---------- ---------- ---------- ----------  2587
F. merguiensis apoCr         ---------- ---------- ---------- ---------- ---------- ---------- ---------- ----------  2586
L. vannamei apoCr            ---------- ---------- ---------- ---------- ---------- ---------- ---------- ----------  2503
P. monodon apoCr             ---------- ---------- ---------- ---------- ---------- ---------- ---------- ----------  2584
M. japonicus apoCr           ---------- ---------- ---------- ---------- ---------- ---------- ---------- ----------  2587
M. ensis apoCr1              ---------- ---------- ---------- ---------- ---------- ---------- ---------- ----------  2583
M. ensis apoCr2              ---------- ---------- ---------- ---------- ---------- ---------- ---------- ----------  2560
C. quadricarinatus apoCr     ---------- ---------- ---------- ---------- ---------- ---------- ---------- ----------  2584
M. rosenbergii apoCr         ---------- ---------- ---------- ---------- ---------- ---------- ---------- ----------  2537
P. hypsinotus apoCr          ---------- ---------- ---------- ---------- ---------- ---------- ---------- ----------  2534
P. trituberculatus apoCr     ---------- ---------- ---------- ---------- ---------- ---------- ---------- ----------  2560
C. feriatus apoCr            ---------- ---------- ---------- ---------- ---------- ---------- ---------- ----------  2579
D. melanogaster apoLpII/I    QVGVIVFEET KRYPALLTSD GGKINYKGNV ADVKLAGIKS FCDNCVEQII TEKRILDIYN SLKEIVKGIA PQADEKAFQL  3177
A. mellifera apoLpII/I       HIGLIGYSEM MKWPQHFTLN GDTNIDGEVK NMKFEEGKPI ISYQEAKEGN TEKKIDYLHQ RMDVELGTF- --KLTDAYEA  3015
L. migratoria apoLpII/I      WISLLGYGAP GQEYPHLYTS SGGKLSYDGK QKNIQFGERK VLGPFPFDNF TES----IDW LDEFTDQAF- --HLITTADT  3201
A. gambiae apoLpII/I         NVAVIGYSKT DKYTSLFSNG GKLDYTGKLG QADVSSGPKQ CRGLVTGIES VDAFLQLLQK MGEQSRENLG ATTEVYALRR  3110
M. sexta apoLpII/I           KVFLVGHTSK HPYPILYDTD LKLKN--AKV SFDDKSRYDR IPFVKTGHEK FDSYSKTVVD FLNYIKIELG ITNIEASQGQ  3127
```

```
P. semisulcatus apoCr        ---------- ---------- ---------- ---------- ---------- ---------- ---------- ----------  2587
F. merguiensis apoCr         ---------- ---------- ---------- ---------- ---------- ---------- ---------- ----------  2586
L. vannamei apoCr            ---------- ---------- ---------- ---------- ---------- ---------- ---------- ----------  2503
P. monodon apoCr             ---------- ---------- ---------- ---------- ---------- ---------- ---------- ----------  2584
M. japonicus apoCr           ---------- ---------- ---------- ---------- ---------- ---------- ---------- ----------  2587
M. ensis apoCr1              ---------- ---------- ---------- ---------- ---------- ---------- ---------- ----------  2583
M. ensis apoCr2              ---------- ---------- ---------- ---------- ---------- ---------- ---------- ----------  2560
C. quadricarinatus apoCr     ---------- ---------- ---------- ---------- ---------- ---------- ---------- ----------  2584
M. rosenbergii apoCr         ---------- ---------- ---------- ---------- ---------- ---------- ---------- ----------  2537
P. hypsinotus apoCr          ---------- ---------- ---------- ---------- ---------- ---------- ---------- ----------  2534
P. trituberculatus apoCr     ---------- ---------- ---------- ---------- ---------- ---------- ---------- ----------  2560
C. feriatus apoCr            ---------- ---------- ---------- ---------- ---------- ---------- ---------- ----------  2579
D. melanogaster apoLpII/I    ALDYPFRAGA AKSIIGVRSD SLEYKNWWKF VRAQLTGSIT KFDGALIHLI APVKG----L SLEGVLSEKL IGFNSRLVAT  3253
A. mellifera apoLpII/I       AIRYPFRPGA ARAVVGVIAN PCEKSPFPIS LQQLR-LLLG LKIYRDLGLT YYHVSYPKEL LVSGKPQKNI VAYDQDNVYT  3094
L. migratoria apoLpII/I      ILDYPFRPGA AKSIIYVLDT SCETTLFLKH LPVKA-LKLK DAIGSPGIVL HLVTN----- -VDSVQSKHI VGFDTNHAYY  3274
A. gambiae apoLpII/I         AFSYPFRASA SKSVLVFRSD SFEIAQPGSA MSAALGMANV KARGIMFNMV APLKSVGLTN TKDQSKVKSI VGFNERVVYH  3190
M. sexta apoLpII/I           IFDLPLRPGA VKHVIFVTGG P--TISQFFL LETVRALRNK VIIDEMAMSA SLVTSTPGLK IGGGKNAAQI VGYEKHGVLL  3205
```

```
P. semisulcatus apoCr         ---------- ---------- ---------- ---------- ---------- ---------- ---------- ----------  2587
F. merguiensis apoCr          ---------- ---------- ---------- ---------- ---------- ---------- ---------- ----------  2586
L. vannamei apoCr             ---------- ---------- ---------- ---------- ---------- ---------- ---------- ----------  2503
P. monodon apoCr              ---------- ---------- ---------- ---------- ---------- ---------- ---------- ----------  2584
M. japonicus apoCr            ---------- ---------- ---------- ---------- ---------- ---------- ---------- ----------  2587
M. ensis apoCr1               ---------- ---------- ---------- ---------- ---------- ---------- ---------- ----------  2583
M. ensis apoCr2               ---------- ---------- ---------- ---------- ---------- ---------- ---------- ----------  2560
C. quadricarinatus apoCr      ---------- ---------- ---------- ---------- ---------- ---------- ---------- ----------  2584
M. rosenbergii apoCr          ---------- ---------- ---------- ---------- ---------- ---------- ---------- ----------  2537
P. hypsinotus apoCr           ---------- ---------- ---------- ---------- ---------- ---------- ---------- ----------  2534
P. trituberculatus apoCr      ---------- ---------- ---------- ---------- ---------- ---------- ---------- ----------  2560
C. feriatus apoCr             ---------- ---------- ---------- ---------- ---------- ---------- ---------- ----------  2579
D. melanogaster apoLpII/I     VDGKDSKK-- ------RTKL QFDNDMGIDF VLNNGGWVFA TQNFEKLKAS DQKKMLNQIT SSLADTLFKT EIVSDCRCLP  3325
A. mellifera apoLpII/I        FADSKKKPLT GSTDMKSNLV PAIKDVCADF AVFSGGAAFS SNNFLDAKSN QKKQFVQVAA KRIADSLVNV EFEKDCSCLY  3174
L. migratoria apoLpII/I       NQEGKKRVVS EVTGNEKAAL KISETACSQI ALATSGTVFN KNN------- -LKQTKKFVA QHIADSLTNV ELTQDCKCLP  3346
A. gambiae apoLpII/I          MNDKKRTLG- -SAEMKKSLK YDDVVAVSAV ERFGGNFFVL QNYAQQKTPK DKKQYISIVA AVLADQLSRT ETTNDCVCYL  3268
M. sexta apoLpII/I            LGEKKQSK-- -DSEAVRATL EVEDDPFSDA VEFANGVVFS ASNYAALPAG QQKQFIQTAA HNIIQRMWRE QIVQQCTCVF  3282


P. semisulcatus apoCr         ---------- ---------- ---------- ---- 2587
F. merguiensis apoCr          ---------- ---------- ---------- ---- 2586
L. vannamei apoCr             ---------- ---------- ---------- ---- 2503
P. monodon apoCr              ---------- ---------- ---------- ---- 2584
M. japonicus apoCr            ---------- ---------- ---------- ---- 2587
M. ensis apoCr1               ---------- ---------- ---------- ---- 2583
M. ensis apoCr2               ---------- ---------- ---------- ---- 2560
C. quadricarinatus apoCr      ---------- ---------- ---------- ---- 2584
M. rosenbergii apoCr          ---------- ---------- ---------- ---- 2537
P. hypsinotus apoCr           ---------- ---------- ---------- ---- 2534
P. trituberculatus apoCr      ---------- ---------- ---------- ---- 2560
C. feriatus apoCr             ---------- ---------- ---------- ---- 2579
D. melanogaster apoLpII/I     IHGLHGQHKC VIKSSTFVAN KKAKSA---- ---- 3351
A. mellifera apoLpII/I        EYGMIGRSKC KIVGRKEVPR SAKGGTKG-- ---- 3202
L. migratoria apoLpII/I       VEGIHTRAVC AVTGAREKEH LSVKGVKGTK GVKG 3380
A. gambiae apoLpII/I          RGGLHPESLC TASDMQVLPP A--------- ---- 3289
M. sexta apoLpII/I            VDPFRVRSVC FNKARTEVAR RRK------- ---- 3305
```
