# Supplementary material for: Apolipocrustacein, formerly vitellogenin, is the major egg yolk precursor protein in decapod crustaceans and is homologous to insect apolipophorin II/I and vertebrate apolipoprotein B
Source: BMC Evol Biol. 2007 Jan 22;7:3. doi: 10.1186/1471-2148-7-3 (PMC1783640; doi:10.1186/1471-2148-7-3)
Supplement: Additional File 2 — Alignment of LLT module conserved protein sequence motifs extracted from LLTP superfamily members. The conservative substitutions allowed were colored and defined as in Additional file 1. Gaps inserted to optimize alignments are indicated by dashes. Single dots indicate missing data. The site of identical conserved amino acids in all sequences is highlighted in red. The name of each conserved motif, from N1 to N22, of the LLT module [15] is indicated below the alignments. [file 1471-2148-7-3-S2.pdf]

Penaeus semisulcatus apoCr	YQPDKTYAYA	YSGKSTVQVA	VVDGRVQHVC	AHPEDEPWAI	NLKKGVASAF	QNETDVVGKC	60
Penaeus monodon apoCr	YQPDKTYAPP	YSGKSRVHVA	VVDGRVQHVC	AHPEDEPWAI	NMKKGVASAF	QNETDVVGKC	60
Metapenaeus ensis apoCr2	YEPGKTYTYA	YSGKSEVQVA	VVDGRVEHVC	AHPDDAPWSV	NMKKGIASII	QNETDVVGKC	60
Fenneropenaeus merguiensis apoCr	YQPDKTYAYA	YSGKSTVQVA	VVDGRVQHVC	AHPEDEAWAI	NLKKGVASAF	QNETDVVGKC	60
Litopenaeus vannamei apoCr	YQPDKTYAYQ	YSGKSKVQVA	VVDGRVQHVC	AHPEDEPWAI	NLKKGVASAF	QNETDVVGKC	60
Marsupenaeus japonicus apoCr	YQPEKTYTYQ	YSGKSRVQVA	VVDGRVQHVC	AHPDDEPWAI	NLKKGVASAF	QNETDVVGKC	60
Metapenaeus ensis apoCr1	YEPGKTYTYA	YSGKSKVQVA	VAEGRVQHVC	AHPEDQPWAI	NLKKGVASAL	QNETDVIGKC	60
Cherax quadricarinatus apoCr	YQPGKTYTYE	YSGKSRIQVA	VTDGKVHHVC	SHPDDDTWSI	NLKKGVASAF	QNETDVVGKC	60
Macrobrachium rosenbergii apoCr	YAPEKTYVYS	YTGKSRTHVS	IIDGRVKAAC	SHPDDDTWSI	NLKKGIASAF	QNETDIIGNC	60
Pandalus hypsinotus apoCr	YAPEKTYVYA	YSGKSRIQVA	MIDGRVQQAC	SHPDDDVWSI	NMKKGIASAF	QNETDIIGNC	60
Portunus trituberculatus apoCr	FIPGKTYSYT	YSGKSIVQVA	MTDGRVQRVC	SHPDDDTWAI	NMKKGVVSAL	QIETDVLGTC	60
Charybdis feriatus apoCr	FTPGKTYSYT	YTGKSQVQVA	MTDGRVQRVC	SHPDDDTWAI	NMKKGVVSAL	QIETDVLGTC	60
Drosophila melanogaster apoLp-II/I	YIPGNYYDYS	FDSILTIGFT	LVSGILEPIC	SDSSDLDYSL	NIKRAVVSLL	QSEVDVFGMC	60
Apis mellifera apoLpII/I	YQEGRTYVYN	LEGLSVTSFN	YHDGHIDTLC	TEPGDSQASL	NIKRAVVSMF	QSETDVMGTC	60
Locusta migratoria apoLp-II/I	YQKGQTYTYS	FEGTTLTSAN	FKGSSINKLC	SEDGVNQSSL	NIKQAILSLL	QTEVDVFGIC	60
Manduca sexta apoLp-II/I	FAAGQKYNYG	VEGTVSVYFS	YQDGRVGPIC	AAEDDSRRSL	NIKRAIISLL	QAQVDVFGVC	60
Anopheles gambiae apoLp-II/I	FKPGFVYQYD	VDSYVQLQFT	LSNDELLPIC	TEADDTDFSL	NVKRGLISLF	QVETDVFGVC	60
Homo sapiens apoB	FKHLRKYTYN	YEAESSSGLA	IPEGKQVFLY	PEKDEPTYIL	NIKRGIISAL	LVLDTVYGNC	60
Danio rerio apoB	FKSFQKYEYT	YETESLNAFT	VEGDNDIKLF	PENDEPANIL	NFKRGLISAL	AAMPTVYGMC	60
Xenopus laevis Vtg	FSESKISVYN	YEAVILNGFE	YSNGRVGDIF	VADDVSDTVA	NIYRGILNLL	QVESSVGGIC	60
Acipenser transmontanus Vtg	FSGSKTYQYK	YEGVILTGFQ	YRNGQVGDIF	ASEDVSDTVL	NIQRGILNML	QLENGIAGIC	60
Gallus gallus Vtg2	FNSRRSYLYN	YEGSMLNGFE	YSSGRIGNIY	APEDCPDLCV	NIVRGILNMF	QMEAGIGGIC	60
Fundulus heteroclitus Vtg	FAAGKTYVYK	YEALILGGLN	TPMVFVGKVF	APEEVSTLVL	NIYRGILNIL	QLEVGTQGVC	60
Oncorhynchus mykiss Vtg	FAASKTYVYK	YEALLLGGFE	YAKGVVGKVL	APTAVSETVL	NVHRGILNIL	QLEAGAQGVC	60
Ichtyomyzon unicuspis Vtg	FQPGKVYRYS	YDAFSISGFD	YSAGRIGDIY	APPQVTDTAV	NIVRGILNLF	QLETGVEGIC	60
Crassostrea gigas Vtg	YESNKEYLYE	YETQALTGFR	YVRGNVKDIH	HEADDPEWSV	NVKKGLLSML	EMEPSIVGEC	60
Mizuhopecten yessoensis Vtg	YSPNKEYWYD	YETQVVTGFS	YIRGHISDLQ	SDPNDPYWSM	DIKRGFISLL	EVEASVAGEC	60
Caenorhabditis elegans Vtg2	FEPKTDYHYK	FDGLVLSGAQ	LRNGLISELQ	FDKEDAEWSK	NMKRAVVNMI	SFEKTLEGDC	60
Caenorhabditis elegans Vtg5	FSPKSEYVYK	FDGLLLSGAQ	IRNGLISEIQ	FSSEDAEWSK	NAKRSILNLF	SLEKTMEGDC	60
Caenorhabditis elegans Vtg6	FRAGREYRYL	FNGQLSAGFD	YEHGLVREIR	FAENDQPWSE	NIKRAVINML	QVERTLEGEC	60
Oscheius sp W Vtg6	YRSGREYRYQ	FNGHLSAGFS	YRHGMVGEIE	FSTEEQTWSS	NIKKAVVNML	QVERTLEGEC	60
Bombyx mori Vtg	WQVGKQYRYE	VTSRTLAHIE	IDGGRIVSLD	FPTSVPVPQE	NLIKGLISAL	QLETDVTGDC	60
Lymantria dispar Vtg	WQNGKQYLYE	VNSHTLSHIS	FDGGRILSLR	LPEVLSVANE	NLLKGLISTL	QVETDVTGDC	60
Riptortus clavatus Vtg	WKAGTKYTYQ	IQGRTLTGIH	FRNGVIEKMV	VNKDIPQWQL	NFYKSIASQF	QVEDSVNGIY	60
Apis mellifera Vtg	WQVGNEYTYL	VRSRTLTSIR	MKHGLIRDLI	VDRDVPTWEV	NILKSIVGQL	QVEDSVGGKC	60
Pimpla nipponica Vtg	WKTDTEYQYA	VRGRTLSAIK	MKHGVVRDLI	VDKDVPTWEV	NVLKSIVSQL	QVEDTVGGRC	60
Daphnia magna Vtg	WMAGYTYEYD	YAGWTSTGVK	FVSGKVETVA	IGKEEPLWIV	NFKRALAAQI	QLEGSVTGEC	60
Aedes aegypti Vtg	WMPNYEYVYN	VTSKTMTAIR	YHKGAIKGLY	VEKTIPNNEV	NILKAWISQL	QVEPLVTGEC	60
Anthonomus grandis Vtg	WKDNTEYVYS	VNGRTLTGLV	LENGLIKRLI	VEKDTLNWEA	NIIKSIVSQF	QMEETVTGKT	60
Penaeus monodon CP	LQPGLEYQYR	YSARVASGVI	MNESHVPSYM	EGPEEPIWIS	NVRKGFVNIF	RVEETLVGRC	60
Pacifastacus leniusculus CP	LHSNLEYQYR	YSGRVASGAI	PGEQRGEEHL	VGPAEPAWIT	NIKKAIVRAF	SIGTFIHGDC	60
Drosophila melanogaster MTP	PNSQQIFKLQ	NQVILQELIS	LVRGQPDKVI	AHTSKDQSLL	NLERGIASLL	QLELDVSGLC	60
Anopheles gambiae MTP	VGTEETFDFS	NVVHVGNGAW	WNLGQIKEVY	FEASDALPVR	NFKKGICALF	QYEVDPSGEC	60
Caenorhabditis elegans MTP	LRKHGPDYYK	NQPKMNENIA	FRQGGNNAIL	KASDESDATW	NFLYAIVNTI	YTVDTIYGRC	60
Danio rerio MTP	NGKLYRYSYG	TEVGLNRPVL	WKMGKIRSLY	AQKAEPATVK	NLKRGVASML	MMEADASGKC	60
Bos taurus MTP	NDRLYKLTYS	TEVFLDRGLH	LIHGKIKEFY	SYQNEPAAIE	NLKRGLASLF	QMEVDISGDC	60
Homo sapiens MTP	NDRLYKLTYS	TEVLLDRGLH	LIHGKVKEFY	SYQNEAVAIE	NIKRGLASLF	QTEVDISGNC	60

N1 **N2** **N3**

```
Penaeus semisulcatus apoCr          PTKYEVVKEK NHRHCQASQD STLRFEAGAL VAKALHVLRR VPEAVVVETA DAV-AFLHES  119
Penaeus monodon apoCr               PTKYEVVKEK NHRHCQASQD STLRFEAAAL VAKALHVLRR VPETIVVETA DAV-AFLHES  119
Metapenaeus ensis apoCr2            PTKYEVTKEK NHRHCHANQE STLRFDAAAL VDKALHMLRR VPAEVVEEVA DAV-AFLHES  119
Fenneropenaeus merguiensis apoCr    PTKYEVVKEK NHRHCQASQY STLRFEAGAL VAKALHVLRR IPDTVVVETA DAV-AFIHES  119
Litopenaeus vannamei apoCr          PTKYEVVKEK NHRHCQASQD STLRFEAAAL VAKALHLLRR VPETVVVETA DAV-AFLHES  119
Marsupenaeus japonicus apoCr        PTTYQVVKEK NHRHCQANQE STLHFEAAAL VAKALHLLRR VPETVVVEIA DAV-AFLHES  119
Metapenaeus ensis apoCr1            PPNTKVTKEK EHRHCTANQE STLRFDAAAL VDKALHMLRS VPAEVVEEVA DAV-AFVHES  119
Cherax quadricarinatus apoCr        PTRYEMIKEK NHRQCKAKMM STLKYDVAAL VARAIQFLRM VPEEAVEQTL DFV-SFVDEP  119
Macrobrachium rosenbergii apoCr     STMYEVTKLK NHRFCQAHQE SVLRFHAASH FAKALHFLRR VPEEAIPQTL DGL-AFVYES  119
Pandalus hypsinotus apoCr           STRYEVTKMK NHRFCQAIQE SELRFTAASH VAKALHYLRR IPEEMVQQTL DAV-AFIHEA  119
Portunus trituberculatus apoCr      PTYYEVKKEK NHRLCKAKQE STLKLDTAAQ LEKAVNLMRR IPEQSFNDIY DAI-AFVHEP  119
Charybdis feriatus apoCr            PTYYEVKKEK NHRLCQAKQE STLRLDVAAH LSKAVHLMRR IPEQGFNEIY DAI-AFVHEP  119
---------------------------------------------------------------------------------------------------
Drosophila melanogaster apoLp-II/I  PTHTSITKAR NLNSCSVVTI LKLKNKKFVE FIRLLRQSDS ETLLELAAFP DGL-FRTSTA  119
Apis mellifera apoLpII/I            PTEFNVNKNR NLASCATNVE TTLTLDAASR FADLVKVLRV SGKNDIMSVY DAL-FRARTG  119
Locusta migratoria apoLp-II/I       PTNVRISKTR NLNRCAVETK LTLTSDAAEK FRTLVSVLRQ SSTTDILKVY DAL-FRTSTG  119
Manduca sexta apoLp-II/I            PTEVSLHRSR DLSRCAVHTK LTLSGKSAGQ FAQLVRIMRT SSKDDLMRIY DAL-LRAGTG  119
Anopheles gambiae apoLp-II/I        QTSFSVEKVR DLGNCAVTTK LTLTGNLFVE LIQLMRYSKK EDLLTLYNQV DAL-FRVGTG  119
---------------------------------------------------------------------------------------------------
Homo sapiens apoB                   STHFTISTER DLGQCDVTQT LKLEDQRANL FNKLVTELRG LSDEAVTSLL QAL-VQCGQP  119
Danio rerio apoB                    RTDYSVVLNR DLSNCDGKQA LTLVGNRAHL AHKLISTIRK MSAESLSAAL QAL-FQCGTP  119
---------------------------------------------------------------------------------------------------
Xenopus laevis Vtg                  HTRYVIIKST DFNNCQMESR QVLAWDAPAK FLQLVQLLRA SNFENLQALW DAL-PMAGTV  119
Acipenser transmontanus Vtg         EASYVVTKSK DLNNCNTEAR QKLVLDAPSK FLQLTQLLRA CTHENIEGIW DAL-PAAATP  119
Gallus gallus Vtg2                  HARYVVTRTV DLNNCQMEAR QQLTLDVSYR FLEVVQLCRI ANADNLESIW SAV-SASGTT  119
Fundulus heteroclitus Vtg           KTLYSLTKTR DLSNCQMRTK QSLEFNAPLK FLELVQLLRI ARYEDLEMYW DTI-PATGTF  119
Oncorhynchus mykiss Vtg             KTHYVLTKSK DLNNCQMEAK QMLTFDAPLK FLQFIQLLRM ASSETINAIW DAV-PSIGSS  119
Ichtyomyzon unicuspis Vtg           QTTYVVVKTK DLNNCDIESR QKLILDAPAK FLRLTAFLRN VDAGVLQSIW DAV-PAMATS  119
Crassostrea gigas Vtg               ETLYRITKVR NYQHCLTYLN QTLNLDRIRK LLKMLEVFMK PTIQPEAGPL DGHPSICGTT  120
Mizuhopecten yessoensis Vtg         ETFYRLNKIR NYNKCVTFIN QTLMLVKIQK LLNSLSAFTE HEIKEEATSL DML-PAAGTP  119
Caenorhabditis elegans Vtg2         QVAYTITKSI NFDKCTTETR SKLVLETSHF LARLVRIFRT TSTSQLKEIH HAL-AIAGTK  119
Caenorhabditis elegans Vtg5         RSRLHYTKSV NFDKCISETR AKVTFETVHL IARAVRMFRM CTIEELKKVH TSI-AVAGTK  119
Caenorhabditis elegans Vtg6         EVLYTWAKSI NLDKCTAFTL NTMELEAPHT VARIVKVLRE CNEEQLEQIY NTL-RLAGTR  119
Oscheius sp W Vtg6                  EVAYTWTKSI NFEKCSARIT NRLELEQPRP RKILLVNYQP CIQARLVKSL DAL-AIAGTK  119
Bombyx mori Vtg                     ETLYTVTKST NYGHCHVYSH VHMELDFLSK FNILVRLIAS MSTEQLSQTS DGV-TQAGTL  119
Lymantria dispar Vtg                ETVYSITKSK NYNRCHVHSY IQMRLEFLTK FNILVRLIST MTTDQLAQFS DAV-IQAGTQ  119
Riptortus clavatus Vtg              ETLYDVVKTR NFSNSNVVSS MNITLNRLPL FSMLTRMVQN ANSQQLEQVT DAV-AQAGTG  119
Apis mellifera Vtg                  EVLYDIAKIK NFDNCDVLSR MNLTLATLEK FMILCNLMRT MNRKQISELE DAI-TQTGTG  119
Pimpla nipponica Vtg                EVLYDIVKTK NYSNCEVSSR VNVTLQTLEK FTLLVRIIRT MSTQQIAEAE DAV-AQAGTA  119
Daphnia magna Vtg                   QTWYHIVKNR DLDNCRGLTV QHLTLEPEIL KKEALRLLSE IISDVESEAY DAL-MLSGTN  119
Aedes aegypti Vtg                   ETHYDVTKTQ NFDRCDVYAQ VNMTLNTMSK FNILTRAIRA MGYEDIYELA DAV-AEAGTP  119
Anthonomus grandis Vtg              ETIYDEGDLI EVIKSKITSM VNVTLHTLDK YTILNTLVRL MDEDDIQFVA DSV-AEAGTG  119
---------------------------------------------------------------------------------------------------
Penaeus monodon CP                  QNWYTVRRTT DFDMCEVDTF TNQTLENLAY IIGRLVEAVA NLDYPYIQTL QLV-IHAGTE  119
Pacifastacus leniusculus CP         MCWYSMSRTV DFDSCEISTF TNQTLDPLVK DMSTLVDAAS ILSRPEIEAL ELL-VTAGTE  119
---------------------------------------------------------------------------------------------------
Drosophila melanogaster MTP          RVSYNTKVEK TKRDCSSSLI LQHVSSLALA YVKLIPLARI TRQEQFEDLL DLL-GAVQTF  119
Anopheles gambiae MTP               ETKYITRYHK SKGNCHYESI MQMNVASATA MVDMVRAARK ATKEDLVRII DIL-GAAQTM  119
Caenorhabditis elegans MTP          FVNFGKRFRR IIEKCDSRTH VEITNKHSLL YSQIAQEARL AKRQDWEAAI SAL-GGVGTA  119
Danio rerio MTP                     LVEYKVIRTK HLETCKVLSR QSLLTEAPRS FLTLVHSLRK SSKSEILTVL DAV-TSAQTP  119
Bos taurus MTP                      KVTYQVTKIK ALDSCKIVSR QKLELEAVRS FLAFIKHLRT AKKEEILQIL DAV-TSAQTP  119
Homo sapiens MTP                    KVTYQVIKIK ALDSCKIVSK QKLELEAVRN FLAFIQHLRT AKKEEILQIL DAV-TSAQTS  119

                                    N4        N5          N6          N7                        N8
```

Penaeus semisulcatus apoCr	GAVKVMVHEI	PVPSLLLAAG	SMVNNYCDTE	VALAIFKAIG	NMGVAPAVTR	AAVHCIESVR	179
Penaeus monodon apoCr	GAVKVMVHEI	PSPSLLLAAA	SMVNNYCDKE	VALAIFKAVG	NMGVIPAVTR	AAVACIESIR	179
Metapenaeus ensis apoCr2	GAVKVMVEEI	PKPTVMLAAA	SMINRYCAKE	EVIAVLKALG	NMGVVPVVAR	AAVSCIEHIR	179
Fenneropenaeus merguiensis apoCr	GAVKVMVNEI	PMPSLLLAAA	TMVNHYCDNE	VALAIFKTIG	NMGVAPAVTR	AAVHCIESVR	179
Litopenaeus vannamei apoCr	GAVQVMVQEI	PVPSVLLAAA	SMINHYCGEE	VPLAFFKAIG	NMGVAPAVTR	AAVQCIESIR	179
Marsupenaeus japonicus apoCr	GAVKVMVQEI	PMPSLLLAAA	SMVNHYCEKE	EALAIFKALG	NMGVVPAVTR	AAAQCIESIR	179
Metapenaeus ensis apoCr1	GAVKVMVEEI	PKPTLMLAAA	SMVNKYCAEE	EIVAALKALG	NMGVVPAVTS	AAVTCIESIR	179
Cherax quadricarinatus apoCr	GAVKVMVKEL	PMPYPTLAAS	SMVNNYCTVQ	AALTTLKALG	NMGVMPAVAT	SVLRCMGRIR	179
Macrobrachium rosenbergii apoCr	GAVKVMVEEL	RFPRTTLAGA	TMVHTYCTRE	HALALLKSLG	NMGVMSEIAR	PILQCIEGIR	179
Pandalus hypsinotus apoCr	GAVKVMVQEL	RFPRTTVAAA	TMVNTYCTRK	EALALLKAMG	NMGVMSEIAK	PIITCIESIR	179
Portunus trituberculatus apoCr	ESVPVMVKEL	DLSSAKLAAA	SMVNTYCTQK	QALSALKSLG	NMGVMPEVAE	KVVLYMESAR	179
Charybdis feriatus apoCr	ESVPVMVKEL	DLSSAKLAAA	SMVNTYCTQK	QALSALKSLG	NMGVMPETAD	KVILYMESTR	179
Drosophila melanogaster apoLp-II/I	ESARVILKQL	APKELYIAVG	NLVAKYCEEE	RIVYILKGLG	NAKSLGNTVA	ALSECASRIR	179
Apis mellifera apoLpII/I	EAAEVGVELI	LPRLGYLGVG	QVIGRYCQEN	SIISALKALG	NSQFLDATLQ	KLANIAARVR	179
Locusta migratoria apoLp-II/I	DAVEVIARLL	LPTEAFLGIG	SFIGRYCGEN	RAIAALKALG	NIRHLNALGE	KVKQLGLRVR	179
Manduca sexta apoLp-II/I	ESIEASIQIL	LPKEVYLGAG	ALGGAYCDED	VVVAILKGIR	NIRHLDSLID	KLVHCAVRVR	179
Anopheles gambiae apoLp-II/I	DAVEAI-TQL	PPREAYLSVG	SLVSKYCQED	VIVAVLKGVR	NSDNLAPLLD	KVIQCAGRVR	178
Homo sapiens apoB	QCSTHILQWL	RSRATLYALS	HAVNNYHDED	YTYLILRVIG	NMGQTEQLTP	ELKSSILMIQ	179
Danio rerio apoB	ECTSAILRVL	SSKPIYYALS	NAVKRLYNQE	HVYLSLRVIG	NMAAAGAASP	ALKSAVIEVQ	179
Xenopus laevis Vtg	DCLKFIKQLI	VHKAAILAYG	TMVRRYCHYE	DIALALKALG	NAGQPESIKR	IQKFLPGRIQ	179
Acipenser transmontanus Vtg	TAFRFITQRI	LRKHAVLAYG	SMVNRYCHEE	ETVLALKALG	NAGQPSSIKR	IQKCLPGKIQ	179
Gallus gallus Vtg2	ETLKFLKNRI	LQQVACLGYS	SVVNRYCRED	KMKLALKCIG	NMGEPASLKR	ILKFLPIHIQ	179
Fundulus heteroclitus Vtg	AGLRFIKEKF	LREVVFLGYG	TMVNKYCEEE	NIILYIKVLG	NAGHPSSFKS	LTKIMPITIH	179
Oncorhynchus mykiss Vtg	VAVRFIKEKF	LRELTMLGYG	TMVSKYCKFE	ELSMVLKALG	NAGHPASIKP	ITKLLPVRVQ	179
Ichtyomyzon unicuspis Vtg	EALLFLKRTL	LRKTAVLGYG	SLVFRYCDEE	EIVLALKALG	NAGQPNSIKK	IQRFLPGRVQ	179
Crassostrea gigas Vtg	SATEVLVDSI	LKRAAFLCLG	SVAGMLRVFD	DKILSFKTIG	NAGLWMIPTI	RTYIQDK-LR	179
Mizuhopecten yessoensis Vtg	AAAYVLATAI	AKIAAWLCMG	SVVNKIN...	....	....	....	146
Caenorhabditis elegans Vtg2	NTIQHILVHM	VRQAAWLAAG	SVVRGIVTTY	EKILALKTIG	NAGLDISVNQ	LNEIIVDPVR	179
Caenorhabditis elegans Vtg5	NTIQHLIHHF	LRQSAWLAAG	SVVRGFASTY	EKVLALKTLG	NAGIDLSVYE	LVQIIQDSIR	179
Caenorhabditis elegans Vtg6	VTIQQFVDKV	LRQSCWLTYG	AIVNGVCTRY	EKVLALKTLA	NAGLDLSVYP	LEKIILNTIR	179
Oscheius sp W Vtg6	VTVSHLLEKI	LRQSCWLSYG	SVLNGVCSRY	EKVLFVKAIA	NAGIDTSVVE	LEKIIRNTVR	179
Bombyx mori Vtg	PAFKQIQSWI	LNSSALMAAT	KLINLGQDST	KAQVYIQAIG	NLGHREILKV	FAPYLEGYLR	179
Lymantria dispar Vtg	PAFQLIETWI	LNSSALIAAA	EFIRLGSDYG	KTLVIVKAIG	TLGHREILKV	FTPYLEGYLR	179
Riptortus clavatus Vtg	PALVTIKQWI	LNTTALFAFT	ELSRKSQDSI	KAQVYIRALG	NTAHPKILAV	FEPYLEGFQR	179
Apis mellifera Vtg	PAFLTIKEWI	LNTAATLSFC	EMIHNAQDSP	RIQTYIMALG	MIGEPKILSV	FEPYLEGFQR	179
Pimpla nipponica Vtg	PALVTITLWI	LNTTALFTFS	ELVRYSQENA	KAHTYIVSLG	NIAHPKILAV	FEPYLEGFQR	179
Daphnia magna Vtg	PSLMLVREYI	LKITTALSLS	RLVYQACDAG	ERMAFLTALG	NIGHEIIVPF	VKPFITSCLV	179
Aedes aegypti Vtg	SAFKLIFDFI	LNATALFAYS	NFVNQAHDSV	KIQVYIRSLG	NLGHPQILSV	FEPYLEGFQR	179
Anthonomus grandis Vtg	PALLNIKKWI	LNQTCILSYT	NLVHKVYDNN	KIHVMIRALG	NIGHKSILNV	FQPYFEGFQR	179
Penaeus monodon CP	PAVNFAVDNL	LMNFATLAHD	ACLSEDRDVW	QRLIYLQALS	NLGTPQTINV	LKPIILGFMR	179
Pacifastacus leniusculus CP	PAIRYLLDKI	LLNLASLAKQ	LCLSSDKSLW	IRLVYIQALA	NLGIPQTIDV	LKHFALGVLR	179
Drosophila melanogaster MTP	DAHNATFGFL	LRESVIQTVA	TLTRQSGTSK	EPTLYIRALQ	NLQDPATIEA	LLEHAQTVAA	179
Anopheles gambiae MTP	ASHQAAKSEL	FYDSLIQCLA	AVTRRYARED	CKLKFIRGLQ	NLKCPRTADR	LIKLAQEVAA	179
Caenorhabditis elegans MTP	ESITTAREVL	EYWKVANTIA	TVLNKRCAGG	VEVRVLEVLE	NIPIFGSYTF	AKKFICEKAA	179
Danio rerio MTP	SSLSAILEFL	IKESVVIIMG	ALLRKLCEES	EVQMYLLALK	NALLPEGIPV	LTKYAESTIA	179
Bos taurus MTP	DSLDAILDFL	IRESVMIIIG	ALVRKLCKKE	DIVMYLLALK	NARLPEGIPL	LLKYTETHLA	179
Homo sapiens MTP	DSLEAILDFL	IRETVMIITG	TLVRKLCKKE	DTRMYLLALK	NALLPEGIPS	LLKYAEAHLA	179

N9 **N10** **N11**

```
Penaeus semisulcatus apoCr           VAAAQAFRQA NCFRPAVEKL VDIAVRPAFE TEVRIASYLA AVRCAEQEHL ETIIERISKE  239
Penaeus monodon apoCr                VAAAHVFRQT KCYRPAVEKL VSIAVRPAFG TEVRIASYLA AIRCAEMEDL EEIFEKISVE  239
Metapenaeus ensis apoCr2             VAAAHVFRQA NCLREPTERL AEIAINEEMG TEERIAAYLG AIRCAEEEDL EKIIPKIIVE  239
Fenneropenaeus merguiensis apoCr     VAAAEAFRQA NCFRPAVEKL VDIAVRPAFE TEVRIASYLA AVRCAEQEHL ETIIEKISKE  239
Litopenaeus vannamei apoCr           VAAAQAFRQA NCFRPAVEKL VDIAVRPAFD TEVRIASYLA AVRCAEQEHL EKIIEKISKE  239
Marsupenaeus japonicus apoCr         VAAAQAFRQA NCDRPAVQKL VDIATRPTFE TEVRIASYLA AIRCAEKEHL EQIIEKISEE  239
Metapenaeus ensis apoCr1             VAAAEVFRQA KCYRPAVEKL VDIATHPDFE TEVRIVSYLA AIKCAEMEDL EKIINKITEE  239
Cherax quadricarinatus apoCr         VAAAQAFRKA KCHRASTGRL VGYALDSRKT TEVRIASYIA AVRCAEKWDF EKIVEKISVG  239
Macrobrachium rosenbergii apoCr      KAATQAFRNV HCHQPIMKQL INVALDHRKK TEVRIGSYLA AIKCAKYEDL HKITEKIAIE  239
Pandalus hypsinotus apoCr            VAATQTFRNV HCHEEFQKQL VNIAVDPTKG TEARIGSYLA AMKCANHEDL RKITNRISVE  239
Portunus trituberculatus apoCr       SRCT-SLQAD RVSRPVTQKL VHYALRPEEN TEVRIAAYLA AVRCANYEDL QEIVTKISYE  238
Charybdis feriatus apoCr             VAAAQAFRLT KCQRLVTQKL VQYALRPGQN TEVRIAAYLA AVRCANYEDL QHIVTKISYE  239
-----------------------------------------------------------------------------------------------------
Drosophila melanogaster apoLp-II/I   VAALHAFSKV KCEETLQSKS LELLKNRNED SELRIEAYLS AISCPNAEVA NQISEIVNSE  239
Apis mellifera apoLpII/I             VAAIQALPTR CSMKWKN-VM FKVLADREED SEIRINTYLS LVACPCPHAA NQLKEVLDKE  238
Locusta migratoria apoLp-II/I        VAALEVIQSD PCRKNIKQAA LQILRDQVED SELRIKAYLA VVECPCDNVV KTISNLLENE  239
Manduca sexta apoLp-II/I             AVALEAFHAD PCSAKIHKTA MDIMKNRQLD SEIRIKAYLA VIECPCSHSA SEIKNLLDSE  239
Anopheles gambiae apoLp-II/I         VAALQAYPAA SCNKKIVNAA LSTLKDTNED SEIRIHAYLS LVECPSANVA NELKALLDAE  238
-----------------------------------------------------------------------------------------------------
Homo sapiens apoB                    KAAIQALRKM EPKDKDQEVL LQTFLDDASP GDKRLAAYLM LMRSPSQADI NKIVQILPWE  239
Danio rerio apoB                     QAAIQVFRLT SVPDEGREVL MKVIFDKAAP IQKRVAAYLI VMKDPQPTEL AQLVAALPNN  239
-----------------------------------------------------------------------------------------------------
Xenopus laevis Vtg                   TDAVMALRNI AKEDKVQEIL LQIFMDRDVR TEVRMMACLA LFETPGLATV TAIANVAARE  239
Acipenser transmontanus Vtg          VDAVMALRNI AKKEKVQELT MQLFMDHQLH SEVRMVASMV LLETPSMALV ATLAEALLKE  239
Gallus gallus Vtg2                   IDAITALKKI AWKDTVQGYL IQILADQSLP PEVRMMACAV IFETPALALI TTIANVAMKE  239
Fundulus heteroclitus Vtg            VEAIMALRNI AKKEMVQELA LQLYMDKALH PELRMLSCIV LFETPSMGLV TTVANSVKTE  239
Oncorhynchus mykiss Vtg              ADAVLALRNI AKREMVQEVA VQLFMDKALH PELRMLACIV LFETPPMGLV ITLASILKTE  239
Ichtyomyzon unicuspis Vtg            AEAIMALRNI AKRDKVQEIV LPIFLNVAIK SELRIRSCIV FFESPSVALV SMVAVRLRRE  239
Crassostrea gigas Vtg                TQAIYSLRKL ARHYDIQTTL LPLYFDQSEK EEVRIGSYLV MTFTPSRQLL EMVAQSLHRE  239
Mizuhopecten yessoensis Vtg          .......... .......... .......... .......... .......... ..........  146
Caenorhabditis elegans Vtg2          KEAIDALRLL KDTMKIQKVL LPIYKNRQYE PEIRMLALWR MMHTPEESLL VQVVSQMEKE  239
Caenorhabditis elegans Vtg5          TEAVDALRLL KDVMKIQKVL LPVYKNRQNK PELRMAALWR MMHTPEEPVL AHIVSQMENE  239
Caenorhabditis elegans Vtg6          TQAIESFRRL RTQMKIQRVL MPVYLNRQQP QHIRMSALHQ IIYTPEWSVL SQIGNQLRQE  239
Oscheius sp W Vtg6                   MQAIDALRRL RLSMKIQNVL MPVYRNHKET PGIRISALHM IMQTPTSGVL DMIVRGLEKE  239
Bombyx mori Vtg                      THIVKNLKTL AKLRHVRAVL FSILRNTAEP YPVRVAAIQS IFISPTGEMM QAMAEMTHND  239
Lymantria dispar Vtg                 VQMILALKPL AEQKYVRSAL FSILMNTAEP YEVRVAAAMI LFLNPTTDML RVMAQLTNDD  239
Riptortus clavatus Vtg               LSIIASLDEM TRTHLARDVL YRIYQNTAEK SEIRAAAVRQ LMRTPPAQML QRMADFTNYD  239
Apis mellifera Vtg                   TLMVGSLGKL TNPKLARSVL YKIYLNTMES HEVRCTAVFL LMKTPPLSML QRMAEFTKLD  239
Pimpla nipponica Vtg                 LLMVISLNKL ATLKLARGVF YRIYKNTGEA HQLRCAAVTA LMSTPPASML QRMAEFTNED  239
Daphnia magna Vtg                    RAKAIFALST LAVKEIVGTL LMPIFFNKAE ETEVRLAALT LLFVSPPQAF WSRVALSTWY  239
Aedes aegypti Vtg                    LAIMVALDNL VIYYLARSVL YRAYQNTADV HEVRCAAVHL LMRTPPADML QRMAEFTHHD  239
Anthonomus grandis Vtg               LMMVACMDRL ADCYIARSVF YKIYQNTAEL PEIRVVAVHQ LIRAPPVEML QRMAQYTNTD  239
-----------------------------------------------------------------------------------------------------
Penaeus monodon CP                   TNAIWSLSAY NMHKQIYEIL MPIIENKGEQ FEIRNIAFLT LATWPGHAWW QQLAVSTWHD  239
Pacifastacus leniusculus CP          TNAILGLSGV YLPESVFGIL MPVFENTGEH NEVRAVAFLA MMTFPSLAWW ERIAISTWRD  239
-----------------------------------------------------------------------------------------------------
Drosophila melanogaster MTP          LQALKAFPLG SFNSSHRLQF ESIFYQRKRR FDARTLALDI ILSLPTQEQL GNFLDYLASN  239
Anopheles gambiae MTP                MKALRSFSVY LWNDEFRAKF EDIFFQVSKR YDARTLALDI LLDLPDQDEL SHLVQFLKSK  239
Caenorhabditis elegans MTP           LNVILAASKN LYETLTHKLI KLFRNTCSQE TPHSQLAIDI LLKCPDHQNV ATLILRTETL  239
Danio rerio MTP                      ITALQRYDPA LITAEVKKAL NRIYHQNQRI YEVRAAAADV IMSSPSYMEV KNLLLSIGHL  239
Bos taurus MTP                       ATTLQRYDVP FITDEVKKTM NRIYHQNRKI HEVRTTAAAI ILKNPSYMEV KNILLSIGEL  239
Homo sapiens MTP                     TTALQRYDLP FITDEVKKTL NRIYHQNRKV HEVRTAAAAI ILNNPSYMDV KNILLSIGEL  239
```

N12 N13 N14 N15

Penaeus semisulcatus apoCr	ETQV-RGFVL	GHLINIQFSR	NIDVAYHAPS	NIIYAPGSFI	PREIGARFVG	IDSIIAELFA	298
Penaeus monodon apoCr	ETQV-RGFIL	GHLLNIQFSR	NIDVAYHAPS	NIIYAPGSFI	PREIGARFEG	IDSIIADLFA	298
Metapenaeus ensis apoCr2	ETQVIRGFVL	SHLLNIQHSR	NVEMSYFAPS	NIIYTPESFL	PREAGIRFEG	LDAIVADVFA	299
Fenneropenaeus merguiensis apoCr	ETQV-RGFVL	GHLINIQFSR	NIDVAYHAPS	NIIYAPGSFV	PREIGARFVG	IESIIAELFA	298
Litopenaeus vannamei apoCr	ETQV-RGFVL	GHLINIQFSR	HIDMAYYAPS	NIIYAPGSFI	PREIGARFEG	VDSIIADIFA	298
Marsupenaeus japonicus apoCr	ETQV-RGFVL	GHLINIQFSR	NVEMSYHAPS	NIIYAPGSFI	PREIGARFEG	IDSIIAEVFA	298
Metapenaeus ensis apoCr1	KTQV-RSFVL	GHLINIQVSR	NVMMGYHSAS	NIIYAPGAFV	PREIGARFEG	VDSIIAEVFA	298
Cherax quadricarinatus apoCr	QTQV-RGFIL	SHLRNVQYSR	NLDLSYFSPS	NIIYAPGSFI	PREVGARFEG	LDPFIADVFA	298
Macrobrachium rosenbergii apoCr	ETQV-RSFIL	SHLHNIKHSR	NIDMSYYAPS	NVIYAPGSYV	PREIGARFEG	LETVVADLYA	298
Pandalus hypsinotus apoCr	ETQV-RGFIL	SHLLNIQYSR	NIDLSYYAPS	NVIYAPGSYV	PREIGARIEG	LEPILADFYA	298
Portunus trituberculatus apoCr	ETQV-RGFIL	SHLINLQYSQ	NLDLSYFSES	NLIYAPGSMI	PREIGARLEG	LEPILADFFA	297
Charybdis feriatus apoCr	ETQV-RGFIL	SHLINLQYSQ	NLDLSYFSPS	NMIYAPGSML	PRDVGARVEG	LEPIIADFFA	298
Drosophila melanogaster apoLp-II/I	TNQV-GGFIS	SNLKAIRYSF	NNEVSYKLEY	QIIYSQHGFL	PREASVRQEN	VEDVLLDVSL	298
Apis mellifera apoLpII/I	TNQV-GSFIQ	THLRNLRYSF	NNELSYKIDS	NVVYSQNSFV	PRELNTRVEN	LDRLLIDLSI	297
Locusta migratoria apoLp-II/I	PIQV-GSFVV	SHLKNLQYSQ	NYELSYAIDS	NVIFSQSSYL	PREIAARTEN	LDHIIVDLSL	298
Manduca sexta apoLp-II/I	PHQV-GNFIT	SSLRHIRYSF	YREMSYKLDQ	TVIYSQTSFL	PRELGGRQGN	LDRVVLDIYV	298
Anopheles gambiae apoLp-II/I	KYQV-GSFIT	SHLASLRYSF	NREFSYAVET	SVIYSQKSFL	PKELEGRQDN	LERLVLDVTM	297
Homo sapiens apoB	QEQV-KNFVA	SHIANILFSR	NYQLYKSVSG	NLIFDPNNYL	PKEIGLEGKG	FEPTLARAYL	298
Danio rerio apoB	KCQA-MSFVN	SHLRNILFSR	NYKI-----G	NVIFESEELL	PNEIGLNGKG	LEPTVAMIYL	293
Xenopus laevis Vtg	SLQL-ASFTF	SQMKALSYSK	VMRVDTFKYV	FIMNSANTMF	PVEIGIRGEG	IEEFLISGYI	298
Acipenser transmontanus Vtg	TLQV-ASFTY	SHMKAITYSK	AMHMDTFKYI	HIINNAASIL	PSEIGLHTEG	LQEVLASAYI	298
Gallus gallus Vtg2	SMQV-ASFVY	SHMKSLSYSK	VIRADTYFDI	FVVNSPRTMF	PSEVGIRVEG	LADVIVSAYL	298
Fundulus heteroclitus Vtg	ELQV-ASFTY	SHMKSLSYSK	AVHVDLYNSA	FYINDAATFM	PKEIGANIEG	LQELIASVYV	298
Oncorhynchus mykiss Vtg	KMQV-ASFTY	SHMMSLTFSQ	AIHLDAYSNA	FYINDAATLF	PREVGVRTEG	IQEALASIYV	298
Ichtyomyzon unicuspis Vtg	PLQV-ASFVY	SQMRSLSYSK	AVHVDTFNAY	FRINSPSGPL	PREFGLRAEG	LQELLASGYV	298
Crassostrea gigas Vtg	RPHV-GTFVY	THLEQMSYSR	FMHLSGFNEL	GLVTTPEEFI	PREIGFNTEG	LQTLVGHMYM	298
Mizuhopecten yessoensis Vtg	....	....	....	....	....	....	146
Caenorhabditis elegans Vtg2	TQQV-AALTH	QMIRHFAASS	YVQLPRFLQF	AAIFEKNSFL	PKQIGFSQQH	MDKYVAMVYL	298
Caenorhabditis elegans Vtg5	SQHV-AAFTY	HVLRQFYLST	YSQLPLFNSF	ATIFEKNAFL	PKQVGFSQQN	FEQVIAVFYL	298
Caenorhabditis elegans Vtg6	RQQV-RAFTL	SLLRSYAESV	YGKWSTYSRF	ASLFTTESVL	PTQIGFTQKN	MEKIIAFVYI	298
Oscheius sp W Vtg6	RQQV-RVYTW	STLKTLSESK	HKTFNWFNMW	ATIFSNDSVL	PKQIGLYQNN	LDSVLAMIYL	298
Bombyx mori Vtg	PVEV-RAVLK	SAILSAAHSF	KFIDDSYDEI	SHIGSEDSLL	PKTIEASFSS	AERFLASFYV	298
Lymantria dispar Vtg	PIHV-RAVLK	SSIETAAYSA	AWFFAKNFDA	NYIGSDGSLI	PNTVGFTSSN	VQDIFSSIYY	298
Riptortus clavatus Vtg	HHQV-NAAVK	SAIESAAYSK	THLKSHNVEC	SSIQSQDSSL	PSKFYFMTSS	SEQAIGNILV	298
Apis mellifera Vtg	TRQV-NSAVK	STIQSLMLSR	GYIDEKILEL	NYVGSEDSVI	PRKVLAMISS	VKSFMGNLMI	298
Pimpla nipponica Vtg	HKHV-NAAVK	SAIESASYSK	LYLTDFIERA	SFISSDDSYV	PESAGAMVSS	AKDLLGAFML	298
Daphnia magna Vtg	EPDQISHFIY	TTIASRVFSE	KTRLGYVTEH	HYSSLSVAMG	PWKPGLRFKS	NKDESLYIYL	299
Aedes aegypti Vtg	PLYV-RAAVK	SAIETAAYSF	NHIRDYALEY	GEIASNDHRY	PSSFYYLVSS	MEAFFGQLLF	298
Anthonomus grandis Vtg	SEEV-NAAVK	SVIESSCQSY	INLRDYVAER	TSHSSAESSF	PKSTGGMISS	IRELLAIIYA	298
Penaeus monodon CP	PPQF-ANFVT	TTIYSISHST	NFFLHEYLFF	AWFASVHGAI	PEKIDGQQRG	LYNLMEELKE	298
Pacifastacus leniusculus CP	PSQV-ANYVS	TTITSLAHSS	MIFLNDYLYF	GWFASNQGLF	PSRVVFGQAE	INKIIDEIVD	298
Drosophila melanogaster MTP	DFEI-KTYVL	QKLRMLAGLT	TVLTRQLSQL	STQEVYQGIL	KRKLGIYTAG	LGSLVGHVWG	298
Anopheles gambiae MTP	DYEL-KQYLL	QKLRMAAGLS	TALSRKFSTT	SLQEMSGGVL	KRTLGLYAGG	MSSFVGHVWS	298
Caenorhabditis elegans MTP	NQEK-WHYLY	KAIEAS-ADS	HVHWQEIADT	ANTEFLQKSF	KRSLSIDTEH	LEQFVSTVWE	297
Danio rerio MTP	PHEM-NKYML	SKIQDVLGSS	SAYSGFMAEL	DILYSGSGVL	RRQVTIEAQG	LESPIMSKMF	298
Bos taurus MTP	PKEM-NKYML	SIVQDILGSS	SAYTGYVERL	DILYSGSGIL	RRQVVIEAQG	LEALIMSKML	298
Homo sapiens MTP	PQEM-NKYML	AIVQDILGSS	SAYTGYIERL	DILYSGSGIL	RRQVVIEAQG	LEALIMSKML	298

N16 **N17** **N18** **N19**

Penaeus semisulcatus apoCr	RFMGHEITYA	DVAESLKLPT	IQGTPLTLKL	PGLSVQATGF	VGFECRFTKV	G	349
Penaeus monodon apoCr	RFMGHEITYA	DVAESLKLPD	IQGTPLRLKL	PGLSVQATGF	VGFACGFTRV	G	349
Metapenaeus ensis apoCr2	RIFGHEVTYA	SIAENLKLPT	IQGTPLKLKL	PSISANVHGF	VGFDCDIAKA	G	350
Fenneropenaeus merguiensis apoCr	RFMGHEITYA	DVAGSLKLPT	IQGTPLRLRL	PGLSVHATGF	VGFDWLLARV	G	349
Litopenaeus vannamei apoCr	RFMGHEITFA	DVAQSLKLPT	IQGTPLKLNL	PGLSVQATGF	VGFECRFTKV	G	349
Marsupenaeus japonicus apoCr	RIMGHEITYA	DVAESLKLPT	IQGTPLKLKL	PALSVQATGF	VGFECRLTRV	G	349
Metapenaeus ensis apoCr1	RIMGQEVTYA	NIAETLKLPT	IQGLHSRSPW	PGLSIHTTGF	VGFDAFIAKA	G	349
Cherax quadricarinatus apoCr	RFMGQEISFA	SLAGDLTFPT	IQGTPFKLKM	PSLSVEVDGF	VGYDCHISKT	G	349
Macrobrachium rosenbergii apoCr	RINNQELAFG	SWEGNMKLPT	MQGLPIKMKL	PSLSTRIDAF	IGYDCHIVRA	G	349
Pandalus hypsinotus apoCr	RVNDQEMAFA	SLAGDLRLPT	MQGVPLKMKL	PSLSSQVDGF	IGFDSHIARV	G	349
Portunus trituberculatus apoCr	RFLGQEINYA	SLSDNLQLPT	IQGTPLKMKL	PSFSVETHGF	IGYDAYISKS	G	348
Charybdis feriatus apoCr	RFLGQEINYA	SLSEHLQFPT	IQGTPLKITS	PSFSVETHGF	IGYDAYISKS	G	349
Drosophila melanogaster apoLp-II/I	KLFGSELAFL	SLGDNIPYPT	SIGVPLELVA	PSVDINANVQ	IGFNAQVLST	G	349
Apis mellifera apoLpII/I	KLFGVEYAYL	SYQGEGSYPT	NLGTALSLGL	PSVSIRLAGS	MTVQAPGVES	G	348
Locusta migratoria apoLp-II/I	KTFGSELAWF	NYDGKHEYPT	NLGFPLKLAI	PSAAVELTGK	LLVDAYVVEG	G	349
Manduca sexta apoLp-II/I	KLFGTDAVFL	SFGDDKGYPT	SVGLPLRLNL	PSTDFEISGA	FIIDADAFST	G	349
Anopheles gambiae apoLp-II/I	KVFGSELFFL	STGENVPYTT	ALGLPLKLSA	PSGSFEVTGT	MSVDAFNVMT	G	348
Homo sapiens apoB	RILGEELGFA	SLHDLQLLPT	GAGLQLQISS	PSVSVEFVTN	MGIIIPDFAR	S	349
Danio rerio apoB	RLLGAEMGYL	KTNDVEDLPT	GTGIPLRVAL	PSAGVEFVTE	VGALLPEYVE	s	344
Xenopus laevis Vtg	KLFGQEIAFT	ELNKEVIIPT	TVGLPAELSL	PSVLVHTVAT	MGINSPLFQA	G	349
Acipenser transmontanus Vtg	KLFGQEISFS	RLDKKTILPT	CIGLPMEMSL	PSIAMHTIAV	MGINTHVIQT	G	349
Gallus gallus Vtg2	KILGQEVAFI	NINKELLVPS	CLGLPLEYGS	PSLYVHTVAT	MGVNTEYFQH	A	349
Fundulus heteroclitus Vtg	KFFGQEIGFA	NIDKPMILPT	VAGIPMELSL	PMVAMDTYAV	MGLNTDIFQA	A	349
Oncorhynchus mykiss Vtg	KFFGQEIAFA	NIDKSIIFPT	AVGLPMELSY	PSIVMHTFAV	MGVNTAFIQA	A	349
Ichtyomyzon unicuspis Vtg	KVHGQEVVFA	ELDKKMMQPV	CIGIPMDLNL	TSMSQHAVAI	MGLTTDLAKA	G	349
Crassostrea gigas Vtg	KMMGNELQYI	TLDGTLVIPS	ECGLPLRLKL	PSVLFQLRGE	MEMDAEYFKM	G	349
Mizuhopecten yessoensis Vtg	....	....	....	....	....	.	146
Caenorhabditis elegans Vtg2	RYKDMDYAFL	PIDRQLVFPT	TLGLPLTISG	PSVAATHVYE	MRMFTPLFEQ	G	349
Caenorhabditis elegans Vtg5	RYKEMDYIVL	PIDMETIIPT	TIGMPLTISG	PTVATTHVTE	MPLLYPVIEQ	G	349
Caenorhabditis elegans Vtg6	RHRDMDYAFL	PIDADSIVPT	PMGLPVQFTS	PRVASTHVLS	LRVICPIAEV	G	349
Oscheius sp W Vtg6	RYKDQDYAFL	PIDIDTLVPS	ALGMPQVMTS	PHVASTHVCK	LELFTPIGGQ	G	349
Bombyx mori Vtg	DFMNNQRLFS	FSESDLQFPV	ASGMPFIFKY	PSKDNKYYEA	NMIKDVQFTY	A	349
Lymantria dispar Vtg	KIFDKEFFWP	LDQPNIKFPI	ATGEPFVFEY	PPTSKDISSN	IQTEIQFTFA	R	349
Riptortus clavatus Vtg	SGLGGKEFFT	FDNHTIEFPS	AMGLPFVYHL	PYLQGENTQA	VYAKAPRWYH	N	349
Apis mellifera Vtg	NNKYALKFFP	FDKHILDFPT	ETGLPFVYTF	PDFAFIVNSN	LRLTFSKNVQ	G	349
Pimpla nipponica Vtg	QSAYNNKFMS	LDKNTLKFPM	ESGFPFVYTM	EDSLDGHVHL	RAVYSVQFQS	K	349
Daphnia magna Vtg	NFLDNYQRFF	TINPNTIIPS	AMGLAYTWAS	PVVVSKMSTR	LMVETPFSRA	Y	350
Aedes aegypti Vtg	KLFNGYFFTA	FDNQTIEWPL	ATGFPFIYTL	PQVYKMPAGH	PETENDDFFY	M	349
Anthonomus grandis Vtg	QVEDVQKLWS	FDNQTLEFPT	EMGLPFLYTY	PAISRNNKLT	KPEQISTEIK	A	349
Penaeus monodon CP	KIGFTSPESA	SDATLWLFPT	EIGIPFSVLY	TSGKMKVNVD	FIFETSQVQF	V	349
Pacifastacus leniusculus CP	QLGVIHTEKE	ADMSFYILPT	DLGLPFMIHY	PTKRKDFADL	TLDFSQDNTL	E	349
Drosophila melanogaster MTP	GSASDSTPAY	QATTLSQQNT	GSAVLGHLAV	LKQTNVRSVF	LQSVDRPYAK	H	349
Anopheles gambiae MTP	GTASEPTPAY	QAITLLQQNT	GISMTSRLSF	LKHRFVGTID	LIGTKHQASR	K	349
Caenorhabditis elegans MTP	ADGRTHKAFE	GHVPVRNYTS	GSLHLTASLL	INQKTVVQDQ	IGKHKKKTLN	R	348
Danio rerio MTP	STSGDPINVV	KGLILLTNRG	ALVIIGNMTV	PFRETVSKQE	KLPTGQMFSR	K	349
Bos taurus MTP	SASSDPMSVV	KGLLLLINRV	SVLITGGITV	PYRQFETKYE	RLSTGRGYIS	R	349
Homo sapiens MTP	SASGDPISVV	KGLILLINRV	TVVITTDITV	PFRQFEKKYE	RLSTGRGYVS	Q	349

N20 **N21** **N22**
